# Microwave-Assisted Stereoselective Heterocyclization to Novel Ring d-fused Arylpyrazolines in the Estrone Series

**DOI:** 10.3390/molecules24030569

**Published:** 2019-02-04

**Authors:** Gergő Mótyán, Barnabás Molnár, János Wölfling, Éva Frank

**Affiliations:** Department of Organic Chemistry, University of Szeged, Dóm tér 8, H-6720 Szeged, Hungary; motyan@chem.u-szeged.hu (G.M.); barnabas.molnar@chem.u-szeged.hu (B.M.); wolfling@chem.u-szeged.hu (J.W.)

**Keywords:** arylpyrazolines, heterocyclization, microwave, stereoselectivity, steroids

## Abstract

Microwave-assisted syntheses of novel ring d-condensed 2-pyrazolines in the estrone series were efficiently carried out from steroidal α,β-enones and hydrazine derivatives. The ring-closure reaction of 16-benzylidene estrone 3-methyl ether with hydrazine in acetic acid resulted in a 2:1 diastereomeric mixture of two 16,17-*cis* fused pyrazolines, which is contrary to the former literature data for both stereoselectivity and product structure. However, the cyclization reactions of a mestranol-derived unsaturated ketone with different arylhydrazines in acidic ethanol furnished the heterocyclic products in good to excellent yields independently of the substituents present on the aromatic ring of the reagents applied. The MW conditions also permitted the ring-closure reaction with *p*-nitrophenylhydrazine which is unfavorable under conventional heating. Moreover, the transformations led to the heterocyclic compounds stereoselectively with a 16α,17α-*cis* ring junction without being susceptible to spontaneous and promoted oxidation to pyrazoles.

## 1. Introduction

Pyrazolines represent an attractive group of five-membered heterocyclic compounds as a consequence of their widespread natural occurrence and diverse pharmacological activities [1,2]. They can be found as structural units in a number of natural products, such as in vitamins, alkaloids and pigments [3]. Moreover, particular attention has been devoted to synthetic derivatives in view of their considerable biological effects and relative simplicity of preparation. Several compounds containing this moiety have been reported to display antifungal [4,5], antibacterial [6,7], antidepressant [8,9], anti-inflammatory [10,11], anticancer [12,13] and anticonvulsant [14,15] properties. They also serve as useful synthons in organic chemistry [16] and precursors for the synthesis of heteroaromatic pyrazoles [17].

**Figure 1:**
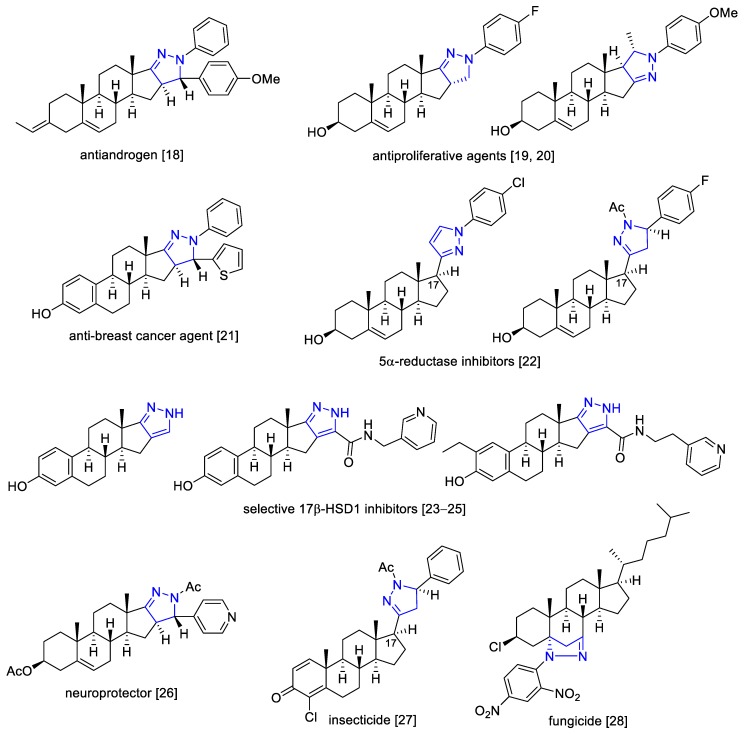
Previously synthesized biologically active steroidal 2-pyrazolines and pyrazoles.

In recent years growing interest has been focused on steroidal 2-pyrazolines and pyrazoles due to their antiandrogenic [18] or direct antiproliferative effect [19,20,21] or their ability to inhibit one of the key regulatory enzymes of steroid biosynthesis [22,23,24,25], which makes them potential candidates for the chemoprevention or treatment of cancerous diseases. Some further derivatives are also known to possess neuroprotective [26], antimicrobial [27] or insecticidal activity [28] (Figure 1).

Although the incorporation of a ring-condensed pyrazoline moiety—most frequently by the reactions of steroidal enones with hydrazine derivatives—is relatively common in the androstane [18,19,20,26,29] and cholestane series [28,30], surprisingly, only a few examples are to be found in the literature for the construction of such heterocycles on estrone-based molecules [21], which may also possess valuable bioactivities. Since the use of MW irradiation can advantageously affect the outcome of various organic syntheses leading to different heterocycles both in product yield and chemoselectivity [31], this technique is often applied for the efficient access to both steroidal and non-steroidal pyrazolines [29,32,33,34]. Considering the high optical purity of chiral steroids, heterocyclization reactions often proceed in a stereoselective manner to afford one of the possible isomers mainly or exclusively, making these reactions more attractive both from a chemical and pharmacological points of view. Steric effect caused by the angular methyl group on C-13 can also exert stereocontrol during the preparation of different ring d-fused analogs [35].

In view of the aforementioned reasons and as we are interested in developing stereoselective synthetic routes on steroid models, we now report a facile access to novel ring d-annelated 2-pyrazolines in the estrone series from a mestranol-derived α,β-enone with various aromatic hydrazines through the application of microwave (MW) irradiation. Our further goal was to investigate the stereoselectivity of the cyclization under acidic conditions, the electronic effect of the substituents of the arylhydrazines on the reaction rate, and the yields of the desired products.

## 2. Results and Discussion

**Scheme 1:**
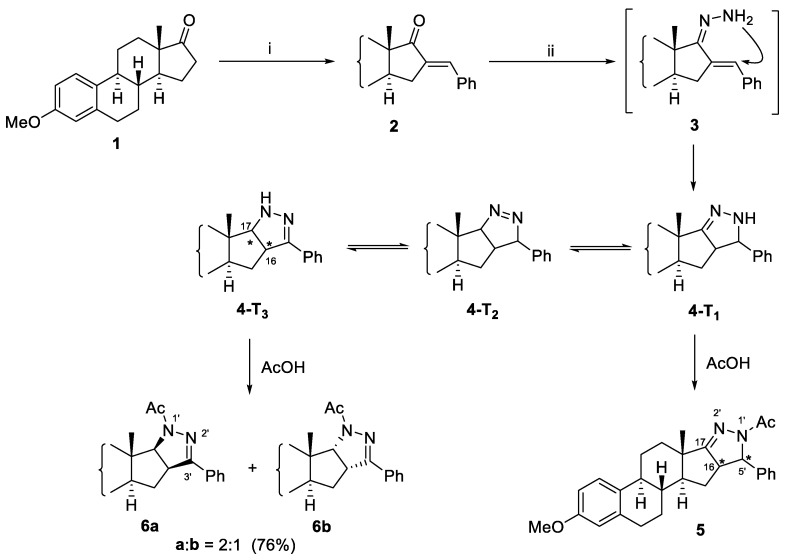
Synthesis of ring d-fused 2-pyrazolines from 16-benzylidene-estrone 3-methyl ether Reagents and conditions: (i) Ph-CHO, KOH, EtOH, MW, 100 °C, 20 min; (ii) N_2_H_4_, AcOH, reflux, 4 h or MW, 120 °C, 20 min.

During preliminary experiments to synthesize novel ring d-fused pyrazolines in the estrone series, 16-benzylidene-estrone 3-methyl ether (**2**) [36], obtained from its precursor (**1**) by MW-assisted Claisen-Schmidt condensation, was reacted with hydrazine hydrate in acetic acid (Scheme 1).

On the basis of relevant references about similar reactions in the androstane series [18,37], a highly diastereoselective ring-closure was expected to occur in this case, leading to a single isomer of **5** despite the formation of two new chiral centers on C-16 and C-5′, with simultaneous acetylation of the heteroring-N(1′) by the solvent. Contrarily, the ^1^H-NMR spectrum of the crude product indicated that a mixture of two acetylated pyrazoline diastereomers was obtained in a ratio of 2:1 under both MW-irradiation at 120 °C for 20 min and conventional heating for 4 h. These findings are in direct contradiction to those by Amr et al. [18] and Romero-López et al. [37], who independently stated that only one of the four possible pyrazoline isomers of **5** was formed in a ca. 70% yield. It is important to note, however, that the results of the abovementioned authors are also in conflict with each other. Although they carried out the cyclization of steroidal 16-benzylidene-17-ones in the androstane series with hydrazine under almost identical conditions, the formation of a 16α-H, 5′β-H isomer of **5** was reported in [18], while that of a 16β-H, 5′β-H was established by the authors of [33]. This inconsistency may arise from the fact that the exact structure and stereo-orientation of the heteroring were elucidated only via determination of the vicinal coupling constant of protons at the newly-formed chirality centers and by the ^1^H-NMR chemical shift of 18-CH_3_ of the isolated compounds. According to our previous results, the cyclization of steroidal α,β-enones with hydrazine hydrate in AcOH proceeds through a hydrazone intermediate, which undergoes intramolecular cyclization before being acetylated by the solvent. This is most likely to occur during the ring-closure of compound **2** (Scheme 1). We also demonstrated earlier that merely the chemical shift of 18-CH_3_ is not indicative of the orientation of a ring d-fused pyrazoline heteroring [29]. Therefore, the mixture of the diastereomers was subjected to column chromatography and both compounds were obtained in almost pure form, sufficient for 1D and 2D NMR studies. Although the formation of two of the four possible stereoisomers of compound **5** could not be excluded on the basis of 1D NMR measurements, 2D NMR spectra clearly demonstrated that instead of **5**, the isomers of pyrazoline **6** were obtained during the cyclization (Supplementary Material). Thus, the major compound **6a** proved to be the 16α-H, 17α-H compound while **6b** was the 16β-H,17β-H diastereomer (Scheme 1). This can be explained by the tendency of 1-unsubstituted Δ^2^-pyrazolines, such as **4**, to tautomerize under heating or in the presence of an acid [38], and the driving force of the tautomeric equilibrium to be shifted toward **4**-**T_3_** is the extended conjugation, especially in product **6** after acetylation. A significant difference (Δ*δ* = 0.37) between the chemical shift values of the equivalent protons of 18-CH_3_ was actually observed by comparing the ^1^H-NMR spectra of **6a** and **6b**. The shielding effect of the pyrazoline ring, which bends toward the angular Me group in **6a**, results in an upfield shift of 18-CH_3_ (*δ* = 0.63 ppm) compared with that of **6b** (*δ* = 1.01 ppm). However, the *J*(16-H,17-H) coupling constants, determined from the doublet of pyrazole 17-H for both compounds, did not differ substantially; 11,4 Hz for **6a** and 9,2 Hz for **6b**, but are in good agreement with the theoretical coupling values calculated according to the Karplus equation for the small (6–7°) dihedral angles *Θ*(H17,C17,C16,H16). The 16,17-*trans* connection of the heteroring is precluded because of ring strain.

**Scheme 2:**
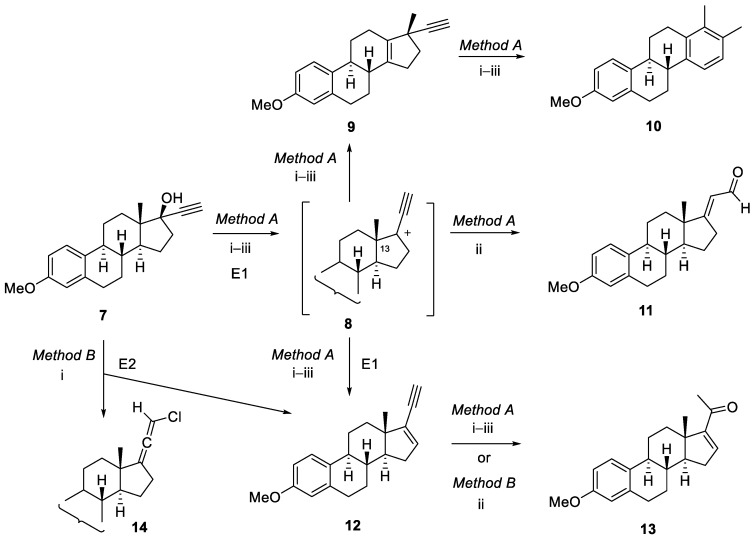
Synthesis of enone **13** from mestranol (**7**): competitive routes under Brønsted acidic conditions (Method A) and a two-step E2-elimination/Markovnikov hydration sequence (Method B). Reagents and conditions: Method A: (i) H_2_SO_4_/SiO_2_, or *p*-toluenesulfonic acid (PTSA)/SiO_2_, toluene, reflux [40]; (ii) HCl, H_2_O, EtOAc, reflux [40]; (iii) HCOOH, reflux [41]; *Method B*: (i) POCl_3_, pyridine, 0 °C → rt, 24 h; (ii) HCOOH, MW, 100 °C, 2 min.

Due to the low stereoselectivity of the above reaction and the difficult separability of the two resulting isomers, a more selective transformation had to be developed for the synthesis of ring d-fused pyrazolines in the estrone series. For this purpose, mestranol (**7**), a clinically applied prodrug for oral contraception and as a component of menopausal hormone therapy, was used as starting material. In principle, compounds containing an α-alkynylcarbinol moiety such as mestranol (**7**), can undergo Rupe rearrangement [39] to provide α,β-unsaturated methyl ketones (**13**) in a single step under acidic conditions (Scheme 2). This reaction involves the protonation of a tertiary alcohol and E1-type dehydration to form a tertiary carbocation (**8**) that expels the α-proton to give an enyne intermediate (**12**). Hydration of the acetylene moiety yields an unsaturated enol, which immediately tautomerizes to the more stable α,β-unsaturated ketone (**13**) [40]. However, in case of Brønsted acid-induced transformation of mestranol (**7**), the Rupe rearrangement is usually accompanied or often replaced by other competitive reactions, such as ring expansion/aromatization reaction leading to a D-homoaromatic compound (**10**) via a Wagner-Meerwein product (**9**) or Meyer-Schuster rearrangement resulting in an α,β-enal (**11**) [41,42]. All of these side-reactions are attributable to the common propargylic cation intermediate (**8**), which can be stabilized in several directions, also taking into account the nearby location of the angular methyl group on C-13.

In order to avoid the formation of by-products in similar or even larger quantities than the desired compound **13**, a two-step protocol had to be applied. Thus, mestranol (**7**) was reacted with phosphorus oxychloride (POCl_3_) in pyridine, acting as both base and solvent, under mild conditions for 24 h to afford enyne **12** via E2 process in good yield [43], together with a small amount of a 21-chloroallenic derivative **14**. The formation of this latter compound can be explained by a OH → Cl interchange at C-17 upon reaction of **7** with POCl_3_, followed by an allylic rearrangement. Similar polarities of **12** and **14** did not allow their complete separation by column chromatography, but the minor impurity did not interfere in the subsequent hydration reaction of **12**, which was carried out in the presence of formic acid (98%) under MW heating at 100 °C for 2 min. The desired α,β-enone **13** was obtained in good yield (75%) referring to **7** in this two-step procedure.

As a continuation, heterocyclization reaction of **13** with phenylhydrazine hydrochloride (**15a**) was carried out in acidic ethanol (Table 1, entry 1). The MW-promoted transformation at 100 °C for 20 min led to the stereoselective formation of a single ring d-condensed pyrazoline isomer (**17a**) in high yield. When the temperature of the MW-induced reaction was elevated to 150 °C, the reaction time was reduced to 5 min to reach complete conversion, but side-reactions became conspicuous in this case. The supposed phenylhydrazone intermediate **16a** of the ring-closure reaction could not be detected because of the use of a closed vessel, therefore its independent synthesis was attempted from **13** with **15a** in the presence of NaOAc by mildly heating the reactants in *^i^*PrOH. Although a less polar intermediate (presumably **16a**) was observed by TLC, it could not be isolated due to its spontaneous cyclization to **17a** under the given conditions.

The ring-closure reaction via 1,4-addition of similar arylhydrazones under conventional heating was earlier observed to be affected significantly by the nature of the substituents: electron-donating groups facilitated, while electron-withdrawing substituents interfered with it by increasing or decreasing the electron density of the intramolecularly attacking internal NH. As a consequence, *p*-nitrophenylhydrazones containing a strong electron-withdrawing NO_2_ group on their aromatic moiety did not cyclize to the corresponding pyrazolines [44]. Therefore, our next goal was to investigate whether the electronic demand of different substituents on the benzene ring of **15a** may have an influence on the yields of the desired products under MW heating. For this reason, cyclization reactions of **13** were also performed with substituted arylhydrazine hydrochlorides **15b**–**j**, and the corresponding ring D-fused pyrazolines **17b**–**j** were obtained in good to excellent yields (Table 1, entries 2–10) even when *p*-nitrophenylhydrazine (**15i**) was used (entry 9).

**Figure 2:**
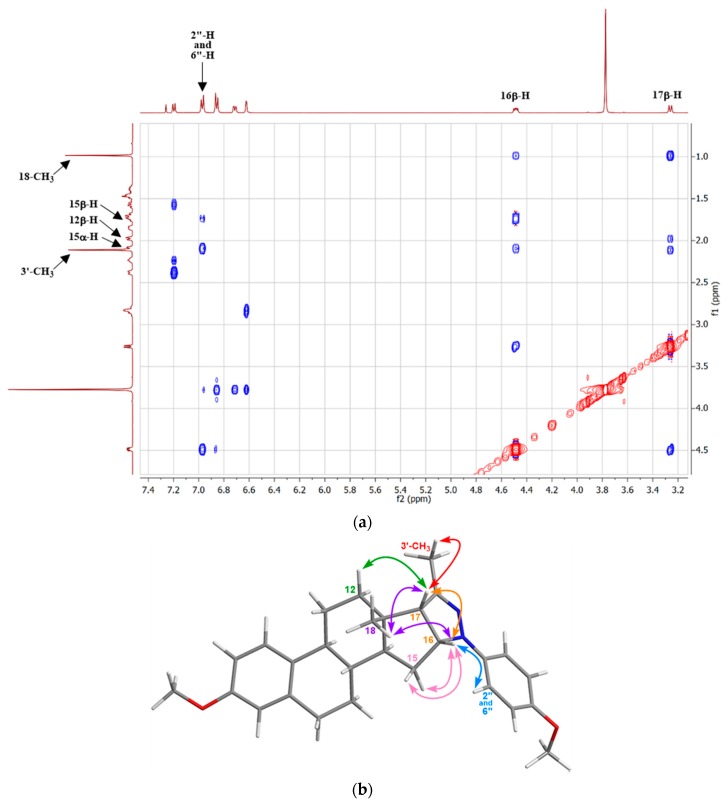
(**a**) Partial NOESY spectrum of compound **17j**; (**b**) NOESY correlations between protons observed for **17j**.

The structures of the synthesized steroidal heterocycles **17a**–**j** were confirmed by NMR and MS measurements. 2D NMR spectra were also recorded for the representative compound **17j**. After identification of the related ^1^H and ^13^C signals with the help of HSQC and HMBC spectra, the 16α,17α-*cis* orientation of the pyrazoline ring was determined on the basis of through-space (NOESY) correlations. Thus, spatial proximity was evidenced by cross-peaks between 16-H–18-H_3_, 17-H–18-H_3_, 16-H–17-H, 17-H–12-H_β_, 17-H–3′-CH_3_, 16-H–2″-H, 16-H–15-H_β_, 16-H–15-H_α_, (Figure 2a,b). The stereoselective ring-closure of the arylhydrazone intermediates can be attributed to the β orientation of the angular Me group on C-13, which permits the intramolecular attack of the NH nitrogen only from the opposite (α) direction.

The synthesized ring d-fused pyrazolines were proved to be resistant against autooxidation to pyrazoles. In addition, dehydrogenation leading to a single heteroaromatic product by oxidizing agents (DDQ, I_2_, Jones reagent, manganese dioxide or iodobenzene diacetate) also failed.

## 3. Materials and Methods

### 3.1. General Information

Reagents and materials were obtained from commercial suppliers (Sigma-Aldrich Corporation, St. Louis, MO, USA; Alfa Aesar, Haverhill, MA, USA or TCI, Tokyo, Japan) and used without purification. All solvents were distilled shortly prior to use. Reactions under MW-irradiation were carried out with a CEM Discover SP, instrument (CEM Corporation, Matthews, NC, USA) using dynamic control program with a maximum power of 200 W. Reactions were monitored by TLC on Kieselgel-G (Si 254 F, Merck KGaA, Darmstadt, Germany) layers (0.25 mm thick); solvent systems (ss): (A) hexane/CH_2_Cl_2_ (10:90 *v*/*v*), (B) hexane/CH_2_Cl_2_ (20:80 *v*/*v*), (C) hexane/CH_2_Cl_2_ (80:20 *v*/*v*), (D) hexane/CH_2_Cl_2_ = 90:10, (E) EtOAc/CH_2_Cl_2_ (2:98 *v*/*v*) and (F) EtOAc/CH_2_Cl_2_ (4:96 *v*/*v*). The spots were detected by spraying with 5% phosphomolybdic acid in 50% aqueous phosphoric acid. Flash chromatography: Merck silica gel 60, 40–63 μm (Merck KGaA, Darmstadt, Germany). Melting points (Mps) were determined on an SRS Optimelt digital apparatus (Stanford Research Systems Inc, Sunnyvale, CA, USA) and are uncorrected. Elementary analysis data were determined with a PerkinElmer CHN analyzer model 2400 (PerkinElmer Inc, Waltham, MA, USA). NMR spectra were obtained at room temperature with a Bruker DRX 500 instrument (Bruker, Billerica, MA, USA). Chemical shifts are reported in ppm (*δ* scale), and coupling constants (*J*) in Hz. The multiplicities of the ^1^H resonance peaks are indicated as a singlet (s), a broad singlet (bs), a doublet (d), a double doublet (dd), a triplet (t), a triplet of doublets (td) or a multiplet (m). ^13^C NMR spectra are ^1^H-decoupled. For the determination of multiplicities, the J-MOD pulse sequence was used. Automated flow injection analyses were performed by using an HPLC/MSD system. The system comprised an Agilent 1100 micro vacuum degasser (Agilent Technologies, Santa Clara, CA, USA) a quaternary pump, a micro-well plate autoinjector and a 1946A MSD equipped with an electrospray ion source (ESI) operated in positive ion mode. The ESI parameters were as follows: nebulizing gas N_2_, at 35 psi; drying gas N_2_, at 350 °C and 12 L/min; capillary voltage 3000 V; fragmentor voltage 70 V. The MSD was operated in scan mode with a mass range of *m*/*z* 60−620. Samples (0.2 μL) with automated needle wash were injected directly into the solvent flow (0.3 mL/min) of CH_3_CN/H_2_O 70:30 (*v*/*v*) supplemented with 0.1% formic acid. The system was controlled by Agilent LC/MSD Chemstation software (C.01.08, Agilent Technologies Inc., Santa Clara, CA, USA).

### 3.2. Synthetic Procedures

#### 3.2.1. MW-assisted Synthesis of 3-methoxy-16-benzylidene-estra-1,3,5(10)-triene-17-one (**2**)

Estrone 3-methyl ether (427 mg, 1.50 mmol) was dissolved in EtOH (6 mL), then KOH (28 mg, 0.5 mmol) and benzaldehyde (0.19 mL, 1.85 mmol) were added to the solution. The mixture was irradiated in a closed vessel at 100 °C for 20 min. After completion of the reaction, the mixture was poured into water (20 mL), and extracted with CH_2_Cl_2_ (2 × 25 mL). The combined organic phases were dried with anhydrous Na_2_SO_4_ and concentrated in vacuo. The crude product was purified by column chromatography with hexane/CH_2_Cl_2_ = 15:85. Yield: 497 mg (white solid); Mp 169–171 °C; *R*_f_ = 0.52 (ss B). Anal. Calcd. for C_26_H_28_O_2_ (372.51): C, 83.83; H, 7.58. Found: C, 83.70; H, 7.68. ^1^H-NMR (CDCl_3_, 500 MHz): *δ* 1.01 (s, 3H, 18-H_3_), 1.48 (m, 1H), 1.55–1.77 (overlapping m, 4H), 2.11 (m, 2H), 2.32 (m, 1H), 2,45 (m, 1H), 2.55 (m, 1H), 2.91–3.03 (m, 3H, 6-H_2_ and 1H), 3.79 (s, 3H, 3-OMe), 6.67 (m, 1H, 4-H), 6.75 (m, 1H, 2-H), 7.23 (m, 1H, 1-H), 7.39 (m, 1H, 4″-H), 7.43 (m, 2H, 3″-H and 5″-H), 7.49 (bs, 1H, CH=), 7.58 (m, 2H, 2″-H and 6″-H); ^13^C-NMR (CDCl_3_, 125 MHz): *δ* 14.5 (C-18), 26.0 (CH_2_), 26.8 (CH_2_), 29.1 (CH_2_), 29.6 (C-6), 31.7 (CH_2_), 38.0 (C-8), 44.0 (C-9), 47.8 (C-13), 48.6 (C-14), 55.2 (3-OMe), 111.6 (C-2), 113.9 (C-4), 126.2 (C-1), 128.6 (2C, C-3″ and C-5″), 129.2 (C-4″), 130.3 (2C, C-2″ and C-6″), 132.0 (C-10), 133.1 (CH=), 135.6 and 136.0 (C-1″ and C-16), 137.6 (C-5), 157.6 (C-3), 209.5 (C-17); ESI-MS 373 [M + H]^+^.

#### 3.2.2. Cyclization of 3-methoxy-16-benzylidene-estra-1,3,5(10)-triene-17-one (**2**) with hydrazine hydrate

To a solution of **2** (343 mg, 0.8 mmol) in acetic acid (5 mL), hydrazine hydrate (0.4 mL, 8.0 mmol) was added and the mixture was irradiated in a closed vessel at 120 °C for 20 min, or stirred at reflux temperature for 4 h. After completion of the reaction, the mixture was poured into water (20 mL), neutralized by the addition of NaHCO_3_ and extracted with EtOAc (2 × 15 mL). The combined organic phases were dried with anhydrous Na_2_SO_4_ and concentrated in vacuo. The crude product was purified by column chromatography with EtOAc/CH_2_Cl_2_ = 2:98, the minor product (**6b**) was eluated first. The ratio of the two 16,17-*cis* fused pyrazolines (**6a** and **6b**) was determined by ^1^H-NMR measurement of the crude product. Overall yield of **6a** and **6b**: 261 mg (white solid).

(16*S*,17*R*)-3-Methoxy-1′-acetyl-3′-phenyl-2′-pyrazolino[4′,5′:16,17]-estra-1,3,5(10)-triene (**6a**): *R*_f_ = 0.46 (ss F). Anal. Calcd. for C_28_H_32_N_2_O_2_ (428.58): C, 78.47; H, 7.53. Found: C, 78.35; H, 7.41. ^1^H-NMR (CDCl_3_, 500 MHz): *δ* 0.63 (s, 3H, 18-H_3_), 1.42–1.57 (overlapping m, 5H, 7α-H, 11β-H, 8β-H, 15β-H and 14α-H), 1.73–1.65 (td, *J* = 13.2, *J* = 3.4 Hz, 1H, 12α-H), 1.92 (m, 1H, 7β-H), 2.27 (m, 1H, 9α-H), 2.33 (m, 1H, 11α-H), 2.39 (m, 1H, 15α-H), 2.40 (s, 3H, Ac-CH_3_), 2.43 (m, 1H, 12β-H), 2.83 (m, 2H, 6-H_2_), 3.76 (s, 3H, 3-OMe), 4.03 (m, 1H, 16α-H), 4.59 (d, 1H, *J* = 11.4 Hz, 17α-H), 6.61 (d, 1H, *J* = 2.4 Hz, 4-H), 6.71 (dd, 1H, *J* = 8.5 Hz, *J* = 2.4 Hz, 2-H), 7.21 (d, 1H, *J* = 8.5 Hz, 1-H), 7.41 (m, 3H, 3″-H, 4″-H and 5″-H), 7.76 (m, 2H, 2″-H and 6″-H); ^13^C-NMR (CDCl_3_, 125 MHz): δ 12.4 (C-18), 21.6 (Ac-CH_3_), 26.6 (C-11), 28.0 (C-7), 29.7 (C-6), 30.7 (C-15), 38.6 (C-8), 39.0 (C-12), 43.4 (C-9), 46.3 (C-13), 49.4 (C-16), 53.4 (C-14), 55.2 (3-OMe), 72.2 (C-17), 111.4 (C-2), 113.8 (C-4), 126.4 (C-1), 126.9 (2C, C-2″ and C-6″), 128.6 (2C, C-3″ and C-5″), 129.9 (C-4″), 131.1 (C-1″), 132.3 (C-10), 137.5 (C-5), 157.5 (2C, C-3 and C-3′), 169.4 (Ac-C); ESI-MS 429 [M + H]^+^.

(16*R*,17*S*)-3-Methoxy-1′-acetyl-3′-phenyl-2′-pyrazolino[4′,5′:16,17]-estra-1,3,5(10)-triene (**6b**) *R*_f_ = 0.46 (ss F). Anal. Calcd. for C_28_H_32_N_2_O_2_ (428.58): C, 78.47; H, 7.53. Found: C, 78.60; H, 7.62. ^1^H-NMR (CDCl_3_, 500 MHz): *δ* 1.00 (s, 3H, 18-H_3_), 1.22 (m, 1H, 14α-H), 1.30 (m, 1H, 7α-H), 1.44 (m, 1H, 8β-H), 1.47–1.55 (overlapping m, 2H, 12α-H and 11β-H), 1.76 (m, 1H, 7β-H), 1.83 (m, 1H, 15β-H), 1.93 (m, 1H, 15α-H), 2.08 (m, 1H, 12β-H), 2.16 (m, 1H, 9α-H), 2.33 (m, 1H, 11α-H), 2.43 (s, 3H, Ac-CH_3_), 2.75–2.87 (overlapping m, 2H, 6-H_2_), 3.76 (s, 3H, 3-OMe), 4.12 (t, 1H, *J* = 9.1 Hz, 16β-H), 4.56 (d, 1H, *J* = 9.1 Hz, 17β-H), 6.59 (d, 1H, *J* = 2.4 Hz, 4-H), 6.71 (dd, 1H, *J* = 8.6 Hz, *J* = 2.4 Hz, 2-H), 7.20 (d, 1H, *J* = 8.6 Hz, 1-H), 7.43 (m, 3H, 3″-H, 4″-H and 5″-H), 7.77 (m, 2H, 2″-H and 6″-H); ^13^C NMR (CDCl_3_, 125 MHz): *δ* 18.5 (C-18), 22.3 (Ac-CH_3_), 26.3 (C-11), 28.1 (C-7), 29.6 (C-6), 31.2 (C-15), 34.0 (C-12), 38.4 (C-8), 43.0 (C-9), 48.2 (C-14), 48.3 (C-13), 48.4 (C-16), 55.2 (3-OMe), 71.6 (C-17), 111.4 (C-2), 113.7 (C-4), 126.4 (C-1), 126.9 (2C, C-2″ and C-6″), 128.7 (2C,C-3″ and C-5″), 129.9 (C-4″), 131.0 (C-1″), 132.4 (C-10), 137.6 (C-5), 157.3 and 157.4 (C-3 and C-3′), 171.3 (Ac-C); ESI-MS 429 [M + H]^+^.

#### 3.2.3. E2-type dehydration of mestranol to 17-ethinyl-3-methoxyestra-1,3,5(10),16-tetraene intermediate **12** by *Method B*

Mestranol (2.48 g, 8.0 mmol) was dissolved in pyridine (30 mL), and POCl_3_ (7 mL) was added dropwise at 0 °C under vigorous stirring. After addition of POCl_3_ (approx. 10 min), the reaction mixture was allowed to warm to room temperature and after 24 h it was poured into a mixture of ice and concentrated H_2_SO_4_ (10 mL), and extracted with EtOAc (3 × 20 mL). The combined organic phases were washed with water (30 mL) and saturated NaHCO_3_ solution (2 × 30 mL), then dried with anhydrous Na_2_SO_4_ and evaporated in vacuo. The resulting crude product was purified by flash chromatography with CH_2_Cl_2_/hexane = 20:80 to give enyne 12 [43] with compound 14 as minor impurity.

*17-(2-Chloroethenylidene)-3-methoxyestra-1,3,5(10)-triene (14) R*_f_ = 0.31 (ss C). Anal. Calcd. for C_21_H_25_ClO (328.88): C, 76.69; H, 7.66. Found: C, 76.60; H, 7.77. ^1^H-NMR (CDCl_3_, 500 MHz): *δ* 0.93 (s, 3H, 18-H_3_), 1.27–1.71 (overlapping m, 7H), 1.93 (m, 2H), 2.14 (m, 1H), 2.28 (m, 1H), 2.39 (m, 1H), 2.62 (m, 1H), 2.89 (m, 2H, 6-H_2_), 3.79 (s, 3H, 3-OMe), 6.03 (s, 1H, 20-H), 6.65 (m, 1H, 4-H), 6.73 (m, 1H, 2-H), 7.23 (m, 1H, 1-H); ^13^C-NMR (CDCl_3_, 125 MHz): *δ* 18.1 (C-18), 24.3 (CH_2_), 26.5 (CH_2_), 27.6 (CH_2_), 27.9 (CH_2_), 29.8 (CH_2_), 35.6 (CH_2_), 38.7 (C-8), 43.7 (C-9), 46.5 (C-13), 53.8 (C-14), 55.2 (3-OMe), 89.2 (C-20), 111.5 (C-2), 113.8 (C-4), 125.4 (C-17), 126.3 (C-1), 132.4 (C-10), 137.8 (C-5), 157.5 (C-3), 193.3 (C-19); ESI-MS 329 [M + H]^+^.

#### 3.2.4. MW-Assisted Syntheses of 3-methoxy-19-norpregna-1,3,5(10),16-tetraene-20-one (**13**)

Enyne 12 (2.45 g, contaminated with a small amount of 14) was dissolved in formic acid (25 mL), and the solution was irradiated in a closed vessel at 100 °C for 2 min. After completion of the reaction, the mixture was poured into water (100 mL), and extracted with EtOAc (3 × 20 mL). The combined organic phases were washed with water (30 mL), followed by saturated NaHCO_3_ solution (30 mL), then dried with anhydrous Na_2_SO_4_ and evaporated in vacuo. The crude product was purified by column chromatography with hexane/CH_2_Cl_2_ = 70:30. Mp 185–187 °C; *R*_f_ = 0.35 (ss D). Anal. Calcd. for C_21_H_26_O_2_ (310.44): C, 81.25; H, 8.44. Found: C, 81.36; H, 8.52. ^1^H-NMR (CDCl_3_, 500 MHz): *δ* 0.92 (s, 3H, 18-H_3_), 1.46 (m, 1H), 1.50–1.69 (overlapping m, 4H), 1.92 (m, 1H), 2.14 (m, 1H), 2.23–2.28 (overlapping m, 2H), 2.29 (s, 3H, 21-CH_3_), 2.34 (m, 1H), 2.41 (m, 1H), 2.52 (m, 1H), 2.89 (m, 2H, 6-H_2_), 3.78 (s, 3H, 3-OMe), 6.64 (d, 1H, *J* = 2.3 Hz, 4-H), 6.72 (dd, 1H, *J* = 8.6 Hz, *J* = 2.3 Hz, 2-H), 6.74 (m, 1H, 16-H), 7.21 (d, 1H, *J* = 8.6 Hz, 1-H); ^13^C-NMR (CDCl_3_, 125 MHz): *δ* 15.9 (C-18), 26.4 (CH_2_), 27.1 (C-20), 27.7 (CH_2_), 29.6 (CH_2_), 31.9 (CH_2_), 34.7 (CH_2_), 36.9 (CH), 44.2 (CH), 46.5 (C-13), 55.2 (3-OMe), 55.5 (CH), 111.3 (C-2), 113.8 (C-4), 126.1 (C-1), 132.7 (C-10), 137.7 (C-5), 144.3 (C-16), 155.5 (C-17), 157.4 (C-3), 196.8 (C-19); ESI-MS 311 [M + H]^+^.

#### 3.2.5. General Procedure for the Synthesis of Ring d-condensed Pyrazolines **17a**–**j** under MW Irradiation

To a solution of 13 (248 mg, 0.80 mmol) in EtOH (5 mL), PTSA monohydrate (152 mg, 0.80 mmol) and (substituted) phenylhydrazine hydrochloride (**15a**–**j**, 1.10 mmol) were added, and the mixture was irradiated in a closed vessel at 100 °C for 20 min. After completion of the reaction, the mixture was poured into water (20 mL), neutralized by the addition of NaHCO_3_ and extracted with CH_2_Cl_2_ (2 × 15 mL). The combined organic phases were dried with anhydrous Na_2_SO_4_ and concentrated in vacuo. The crude product was purified by column chromatography with hexane/CH_2_Cl_2_ = 50:50.

*(16R,17S)-3-Methoxy-3′-methyl-1′-phenyl-2′-pyrazolino[4′,5′:17,16]estra-1,3,5(10)-triene* (**17a**). According to the general procedure, phenylhydrazine hydrochloride (**15a**, 159 mg) was used. Yield: 285 mg (white solid); Mp 221–223 °C; *R*_f_ = 0.58 (ss A). Anal. Calcd. for C_27_H_32_N_2_O (400.57): C, 80.96; H, 8.05. Found: C, 80.81; H, 8.16. ^1^H-NMR (CDCl_3_, 500 MHz): *δ* 1.00 (s, 3H, 18-H_3_), 1.34–1.52 (overlapping m, 3H, 7α-H, 8β-H and 14α-H), 1.58 (m, 1H, 11β-H), 1.68–1.78 (overlapping m, 2H, 12α-H and 15β-H), 1.85 (m, 1H, 7β-H), 1.99 (m, 1H, 12β-H), 2.12 (s + m, 4H, 3′-CH_3_ and 15α-H), 2.23 (m, 1H, 9α-H), 2.39 (m, 1H, 11α-H), 2.84 (m, 2H, 6-H_2_), 3.28 (d, 1H, *J* = 10.0 Hz, 17β-H), 3.78 (s, 3H, 3-OMe), 4.55 (dd, 1H, *J* = 10.0 Hz, *J* = 5.9 Hz, 16β-H), 6.62 (d, 1H, *J* = 2.3 Hz, 4-H), 6.72 (dd, 1H, *J* = 8.6 Hz, *J* = 2.3 Hz, 2-H), 6.78 (t-like m, 1H, 4″-H), 7.01 (d, 2H, *J* = 8.4 Hz, 2″-H and 6″-H), 7.20 (d, 1H, *J* = 8.6 Hz, 1-H), 7.25 (t-like m, 2H, 3″-H and 5″-H); ^13^C-NMR (CDCl_3_, 125 MHz): *δ* 17.1 (3′-CH_3_), 21.5 (C-18), 26.5 (C-11), 28.1 (C-7), 29.7 (C-6), 33.5 (C-15), 36.0 (C-12), 38.5 (C-8), 43.3 (C-9), 46.9 (C-13), 49.6 (C-14), 55.2 (3-OMe), 64.0 (C-16), 66.4 (C-17), 111.5 (C-2), 111.7 (2C, C-2″ and C-6″), 113.7 (C-4), 117.7 (C-4″), 126.2 (C-1), 129.0 (2C, C-3″ and C-5″), 132.2 (C-10), 137.9 (C-5), 144.9 (C-1″), 149.3 (C-3′), 157.5 (C-3); ESI-MS 401 [M + H]^+^.αβ

*(16R,17S)-3-Methoxy-3′-methyl-1′-(4″-tolyl)-2′-pyrazolino[4′,5′:17,16]estra-1,3,5(10)-triene* (**17b**). According to the general procedure, 4-tolylhydrazine hydrochloride (**15b**, 174 mg) was used. Yield: 315 mg (white solid); Mp 246–248 °C; *R*_f_ = 0.49 (ss B). Anal. Calcd. for C_28_H_34_N_2_O (414.59): C, 81.12; H, 8.27. Found: C, 81.03; H, 8.18. ^1^H-NMR (CDCl_3_, 500 MHz): *δ* 0.99 (s, 3H, 18-H_3_), 1.36 (m, 1H, 7α-H), 1.42–1.51 (overlapping m, 2H, 8β-H and 14α-H), 1.57 (m, 1H, 11β-H), 1.68–1.76 (overlapping m, 2H, 12α-H and 15β-H), 1.84 (m, 1H, 7β-H), 1.98 (m, 1H, 12β-H), 2.11 (s + m, 4H, 3′-CH_3_ and 15α-H), 2.23 (m, 1H, 9α-H), 2.28 (s, 3H, 4″-CH_3_), 2.38 (m, 1H, 11α-H), 2.83 (m, 2H, 6-H_2_), 3.26 (d, 1H, *J* = 10.0 Hz, 17β-H), 3.78 (s, 3H, 3-OMe), 4.53 (dd, 1H, *J* = 10.0 Hz, *J* = 5.8 Hz, 16β-H), 6.63 (d, 1H, *J* = 2.6 Hz, 4-H), 6.72 (dd, 1H, *J* = 8.3 Hz, *J* = 2.6 Hz, 2-H), 6.92 (d, 2H, *J* = 8.2 Hz, 3″-H and 5″-H), 7.08 (d, 2H, *J* = 8.2 Hz, 2″-H and 6″-H), 7.20 (d, 1H, *J* = 8.3 Hz, 1-H); ^13^C-NMR (CDCl_3_, 125 MHz): *δ* 17.1 (3′-CH_3_), 20.4 (4″-CH_3_), 21.5 (C-18), 26.5 (C-11), 28.1 (C-7), 29.8 (C-6), 33.6 (C-15), 36.0 (C-12), 38.5 (C-8), 43.3 (C-9), 46.9 (C-13), 49.6 (C-14), 55.2 (3-OMe), 64.4 (C-16), 66.4 (C-17), 111.5 (C-2), 111.8 (2C, C-2″ and C-6″), 113.8 (C-4), 126.2 (C-1), 126.9 (C-4″), 129.6 (2C, C-3″ and C-5″), 132.3 (C-10), 137.9 (C-5), 142.9 (C-1″), 148.8 (C-3′), 157.5 (C-3); ESI-MS 415 [M + H]^+^.

*(16R,17S)-3-Methoxy-3′-methyl-1′-(2″-tolyl)-2′-pyrazolino[4′,5′:17,16]estra-1,3,5(10)-triene* (**17c**). According to the general procedure, 2-tolylhydrazine hydrochloride (**15c**, 174 mg) was used. Yield: 249 mg (white solid); Mp 73–76 °C; *R*_f_ = 0.61 (ss E). Anal. Calcd. for C_28_H_34_N_2_O (414.59): C, 81.12; H, 8.27. Found: C, 81.26; H, 8.39. ^1^H-NMR (CDCl_3_, 500 MHz): *δ* 0.96 (s, 3H, 18-H_3_), 1.20–1.42 (overlapping m, 3H), 1.47 (m, 1H), 1.56 (m, 1H), 1.67–1.77 (overlapping m, 3H), 1.96 (m, 1H), 2.11 (s, 3H, 3′-CH_3_), 2.22 (m, 1H), 2.32 (s, 3H, 2″-CH_3_), 2.38 (m, 1H), 2.78 (m, 2H, 6-H_2_), 3.24 (d, 1H, *J* = 9.9 Hz, 17β-H), 3.77 (s, 3H, 3-OMe), 4.77 (dd, 1H, *J* = 9.9 Hz, *J* = 6.3 Hz, 16β-H), 6.61 (d, 1H, *J* = 2.6 Hz, 4-H), 6.72 (dd, 1H, *J* = 8.6 Hz, *J* = 2.6 Hz, 2-H), 6.95 (m, 1H), 7.09 (m, 1H), 7.14 (overlapping m, 2H), 7.19 (d, 1H, *J* = 8.6 Hz, 1-H); ^13^C-NMR (CDCl_3_, 125 MHz): *δ* 17.0 (3′-CH_3_), 20.2 (2″-CH_3_), 21.5 (C-18), 26.4 (C-11), 27.9 (C-7), 29.6 (C-6), 32.3 (C-15), 35.7 (C-12), 38.5 (C-8), 43.3 (C-9), 46.7 (C-13), 49.9 (C-14), 55.2 (3-OMe), 65.5 (C-16), 66.4 (C-17), 111.5 (C-2), 113.7 (C-4), 120.0 (CH), 122.5 (C-H), 126.1 (C-H), 126.2 (C-1), 129.3 (C-2″), 131.1 (C-H), 132.4 (C-10), 137.8 (C-5), 144.6 (C-1″), 149.8 (C-3′), 157.5 (C-3); ESI-MS 415 [M + H]^+^.

*(16R,17S)-3-Methoxy-3′-methyl-1′-(2″,4″-dimethylphenyl)-2′-pyrazolino[4′,5′:17,16]estra-1,3,5(10)-triene* (**17d**). According to the general procedure, 2,4-dimethylphenylhydrazine hydrochloride (**15d**, 190 mg) was used. Yield: 274 mg (white solid); Mp 214–216 °C; *R*_f_ = 0.55 (ss E). Anal. Calcd. for C_29_H_36_N_2_O (428.62): C, 81.27; H, 8.47. Found: C, 81.37; H, 8.38. ^1^H-NMR (CDCl_3_, 500 MHz): *δ* 0.95 (s, 3H, 18-H_3_), 1.23–1.47 (overlapping m, 4H), 1.56 (m, 1H), 1.68–1.78 (overlapping m, 3H), 1.96 (m, 1H), 2.11 (s, 3H, 3′-CH_3_), 2.22 (m, 1H), 2.28 (s, 6H, 2″-CH_3_ and 4″-CH_3_), 2.38 (m, 1H), 2.79 (m, 2H, 6-H_2_), 3.23 (d, 1H, *J* = 9.7 Hz, 17β-H), 3.77 (s, 3H, 3-OMe), 4.70 (dd, 1H, *J* = 9.7 Hz, *J* = 6.3 Hz, 16β-H), 6.61 (d, 1H, *J* = 2.2 Hz, 4-H), 6.72 (dd, 1H, *J* = 8.5 Hz, *J* = 2.2 Hz, 2-H), 6.90–7.02 (overlapping m, 3H, 3″-H, 5″-H and 6″-H), 7.20 (d, 1H, *J* = 8.5 Hz, 1-H); ^13^C-NMR (CDCl_3_, 125 MHz): *δ* 17.0 (3′-CH_3_), 19.8 (2″-CH_3_), 20.7 (4″-CH_3_), 21.5 (C-18), 26.4 (C-11), 28.0 (C-7), 29.6 (C-6), 32.3 (C-15), 35.7 (C-12), 38.5 (C-8), 43.4 (C-9), 46.7 (C-13), 49.9 (C-14), 55.2 (3-OMe), 66.0 (C-16), 66.5 (C-17), 111.4 (C-2), 113.7 (C-4), 120.7 (C-6″), 126.1 (C-1), 126.8 (C-5″), 129.8 (C-2″), 131.7 (C-3″), 132.3 (C-4″), 132.4 (C-10), 137.9 (C-5), 142.4 (C-1″), 149.6 (C-3′), 157.5 (C-3); ESI-MS 429 [M + H]^+^.

*(16R,17S)-3-Methoxy-3′-methyl-1′-(4″-fluorophenyl)-2′-pyrazolino[4′,5′:17,16]estra-1,3,5(10)-triene* (**17e**). According to the general procedure, 4-fluorophenylhydrazine hydrochloride (**15e**, 179 mg) was used. Yield: 295 mg (white solid); Mp 193–195 °C; *R*_f_ = 0.57 (ss B). Anal. Calcd. for C_27_H_31_FN_2_O (418.56): C, 77.48; H, 7.47. Found: C, 77.39; H, 7.59. ^1^H-NMR (CDCl_3_, 500 MHz): *δ* 0.99 (s, 3H, 18-H_3_), 1.35–1.51 (overlapping m, 3H, 7α-H, 8β-H and 14α-H), 1.57 (m, 11β-H), 1.67–1.77 (overlapping m, 2H, 12α-H and 15β-H), 1.85 (m, 1H, 7β-H), 1.98 (m, 1H, 12β-H), 2.07 (dd, 1H, *J* = 12.4 Hz, *J* = 5.3 Hz, 15α-H), 2.10 (s, 3H, 3′-CH_3_), 2.23 (m, 1H, 9α-H), 2.38 (m, 1H, 11α-H), 2.84 (m, 2H, 6-H_2_), 3.27 (d, 1H, *J* = 10.1 Hz, 17β-H), 3.78 (s, 3H, 3-OMe), 4.49 (dd, 1H, *J* = 10.1 Hz, *J* = 5.9 Hz, 16β-H), 6.63 (d, 1H, *J* = 2.5 Hz, 4-H), 6.72 (dd, 1H, *J* = 8.6 Hz, *J* = 2.5 Hz, 2-H), 6.91–6.98 (m, 4H, 2″-H, 6″-H and 3″-H, 5″-H), 7.20 (d, 1H, *J* = 8.6 Hz, 1-H); ^13^C-NMR (CDCl_3_, 125 MHz): *δ* 17.0 (3′-CH_3_), 21.5 (C-18), 26.5 (C-11), 28.1 (C-7), 29.7 (C-6), 33.6 (C-15), 36.1 (C-12), 38.5 (C-8), 43.3 (C-9), 46.8 (C-13), 49.7 (C-14), 55.2 (3-OMe), 64.7 (C-16), 66.7 (C-17), 111.5 (C-2), 112.6 (2C, *J* = 7.1 Hz, C-2″ and C-6″), 113.8 (C-4), 115.5 (2C, *J* = 22.2 Hz, C-3″ and C-5″), 126.2 (C-1), 132.2 (C-10), 137.8 (C-5), 141.8 (C-1″), 149.4 (C-3′), 156.1 (*J* = 235.7 Hz, C-4″), 157.5 (C-3); ESI-MS 419 [M + H]^+^.

*(16R,17S)-3-Methoxy-3′-methyl-1′-(4″-chlorophenyl)-2′-pyrazolino[4′,5′:17,16]estra-1,3,5(10)-triene* (**17f**). According to the general procedure, 4-chlorophenylhydrazine hydrochloride (**15f**, 197 mg) was used. Yield: 282 mg (white solid); Mp 245–248 °C; *R*_f_ = 0.64 (ss B). Anal. Calcd. for C_27_H_31_ClN_2_O (435.01): C, 74.55; H, 7.18. Found: C, 74.69; H, 7.28. ^1^H-NMR (CDCl_3_, 500 MHz): *δ* 0.99 (s, 3H, 18-H_3_), 1.34–1.51 (overlapping m, 3H, 7**α**-H, 8**β**-H, 14**α**-H), 1.57 (m, 1H, 11**β**-H), 1.66–1.77 (overlapping m, 2H, 12**α**-H, 15**β**-H), 1.84 (m, 1H, 7**β**-H), 1.99 (m, 1H, 12**β**-H), 2.06 (dd, 1H, *J* = 12.5 Hz, *J* = 5.6 Hz, 15**β**-H), 2.11 (s, 3H, 3′-CH_3_), 2.23 (m, 1H, 9**α**-H), 2.38 (m, 1H, 11**α**-H), 2.84 (m, 2H, 6-H_2_), 3.28 (d, 1H, *J* = 10.0 Hz, 17**β**-H), 3.78 (s, 3H, 3-OMe), 4.50 (dd, 1H, *J* = 10.0 Hz, *J* = 5.9 Hz, 16**β**-H), 6.63 (d, 1H, *J* = 2.6 Hz, 4-H), 6.72 (dd, 1H, *J* = 8.6 Hz, *J* = 2.6 Hz, 2-H), 6.92 (d, 2H, *J* = 8.8 Hz, 2″-H and 6″-H), 7.19 (d, 1H, *J* = 8.6 Hz, 1-H and d, 2H, *J* = 8.8 Hz, 3″-H and 5″-H); ^13^C NMR (CDCl_3_, 125 MHz): *δ* 17.0 (3′-CH_3_), 21.4 (C-18), 26.4 (C-11), 28.0 (C-7), 29.7 (C-6), 33.4 (C-15), 36.0 (C-12), 38.5 (C-8), 43.3 (C-9), 46.9 (C-13), 49.6 (C-14), 55.2 (3-OMe), 64.0 (C-16), 66.6 (C-17), 111.5 (C-2), 112.9 (2C, C-2″ and C-6″), 113.7 (C-4), 122.4 (C-4″), 126.2 (C-1), 128.8 (2C, C-3″ and C-5″), 132.2 (C-10), 137.8 (C-5), 143.4 (C-1″), 149.9 (C-3′), 157.5 (C-3); ESI-MS 436 [M + H]^+^.

*(16R,17S)-3-Methoxy-3′-methyl-1′-(4″-bromophenyl)-2′-pyrazolino[4′,5′:17,16]estra-1,3,5(10)-triene* (**17g**). According to the general procedure, 4-bromophenylhydrazine hydrochloride (**15g**, 246 mg) was used. Yield: 318 mg (white solid); Mp 231–232 °C; *R*_f_ = 0.64 (ss B). Anal. Calcd. for C_27_H_31_BrN_2_O (479.46): C, 67.64; H, 6.52. Found: C, 67.75; H, 6.39. ^1^H-NMR (CDCl_3_, 500 MHz): *δ* 0.99 (s, 3H, 18-H_3_), 1.33–1.50 (overlapping m, 3H, 7α-H, 8β-H, 14α-H), 1.57 (m, 1H, 11β-H), 1.67–1.77 (overlapping m, 2H, 12α-H, 15β-H), 1.84 (m, 1H), 1.98 (m, 1H, 12β-H), 2.06 (dd, 1H, *J* = 12.6 Hz, *J* = 5.7 Hz, 15α-H), 2.10 (s, 3H, 3′-CH_3_), 2.22 (m, 1H, 9α-H), 2.38 (m, 1H, 11α-H), 2.83 (m, 2H, 6-H_2_), 3.28 (d, 1H, *J* = 10.0 Hz, 17β-H), 3.78 (s, 3H, 3-OMe), 4.49 (dd, 1H, *J* = 10.0 Hz, *J* = 5.9 Hz, 16β-H), 6.63 (d, 1H, *J* = 2.7 Hz, 4-H), 6.72 (dd, 1H, *J* = 8.6 Hz, *J* = 2.7 Hz, 2-H), 6.87 (d, 2H, *J* = 9.0 Hz, 2″-H and 6″-H), 7.19 (d, 1H, *J* = 8.6 Hz, 1-H), 7.32 (d, 2H, *J* = 9.0 Hz, 3″-H and 5″-H); ^13^C-NMR (CDCl_3_, 125 MHz): *δ* 17.0 (3′-CH_3_), 21.5 (C-18), 26.5 (C-11), 28.1 (C-7), 29.7 (C-6), 33.3 (C-15), 36.0 (C-12), 38.5 (C-8), 43.3 (C-9), 46.9 (C-13), 49.6 (C-14), 55.2 (3-OMe), 63.9 (C-16), 66.6 (C-17), 109.6 (C-4″), 111.5 (C-2), 113.4 (2C, C-2″ and C-6″), 113.8 (C-4), 126.2 (C-1), 131.7 (2C, C-3″ and C-5″), 132.2 (C-10), 137.8 (C-5), 143.7 (C-1″), 150.0 (C-3′), 157.6 (C-3); ESI-MS 480 [M + H]^+^.

*(16R,17S)-3-Methoxy-3′-methyl-1′-(4″-cyanophenyl)-2′-pyrazolino[4′,5′:17,16]estra-1,3,5(10)-triene* (**17h**). According to the general procedure, 4-cyanophenylhydrazine hydrochloride (**15h**, 187 mg) was used. Yield: 289 mg (white solid); Mp 214–216 °C; *R*_f_ = 0.64 (ss E). Anal. Calcd. for C_28_H_31_N_3_O (425.58): C, 79.02; H, 7.34. Found: C, 79.12; H, 7.20. ^1^H-NMR (CDCl_3_, 500 MHz): *δ* 1.01 (s, 3H, 18-H_3_), 1.32–1.41 (overlapping m, 2H, 7**α**-H and 14**α**-H), 1.47 (m, 1H, 8**β**-H), 1.58 (m, 1H, 11**β**-H), 1.69 (m, 1H, 12**α**-H), 1.77 (m, 1H, 15**β**-H), 1.83 (m, 1H, 7**β**-H), 1.98–2.08 (overlapping m, 2H, 12**β**-H and 15**α**-H), 2.14 (s, 3H, 3′-CH_3_), 2.22 (m, 1H, 9**α**-H), 2.39 (m, 1H, 11**α**-H), 2.83 (m, 2H, 6-H_2_), 3.33 (d, 1H, *J* = 9.6 Hz, 17**β**-H), 3.77 (s, 3H, 3-OMe), 4.58 (dd, 1H, *J* = 9.6 Hz, *J* = 6.3 Hz, 16**β**-H), 6.62 (d, 1H, *J* = 2.2 Hz, 4-H), 6.72 (dd, 1H, *J* = 8.6 Hz, *J* = 2.2 Hz, 2-H), 6.96 (d, 2H, *J* = 8.5 Hz, 2″-H and 6″-H), 7.19 (d, 1H, *J* = 8.6 Hz, 1-H), 7.48 (d, 2H, *J* = 8.5 Hz, 3″-H and 5″-H); ^13^C-NMR (CDCl_3_, 125 MHz): *δ* 17.1 (3′-CH_3_), 21.4 (C-18), 26.4 (C-11), 28.1 (C-7), 29.6 (C-6), 33.1 (C-15), 35.9 (C-12), 38.5 (C-8), 43.2 (C-9), 46.9 (C-13), 49.5 (C-14), 55.2 (3-OMe), 63.1 (C-16), 66.7 (C-17), 99.0 (C-4″), 111.6 (C-2), 111.6 (2C, C-2″ and C-6″), 113.7 (C-4), 120.5 (4″-CN), 126.1 (C-1), 131.9 (C-10), 133.4 (2C, C-3″ and C-5″), 137.7 (C-5), 146.7 (C-1″), 152.6 (C-3′), 157.6 (C-3); ESI-MS 426 [M + H]^+^.

*(16R,17S)-3-Methoxy-3′-methyl-1′-(4″-nitrophenyl)-2′-pyrazolino[4′,5′:17,16]estra-1,3,5(10)-triene* (**17i**). According to the general procedure, 4-nitrophenylhydrazine hydrochloride (**15i**, 209 mg) was used. Yield: 285 mg (yellow solid); Mp 262–263 °C; *R*_f_ = 0.32 (ss B). Anal. Calcd. for C_27_H_31_N_3_O_3_ (445.56): C, 72.78; H, 7.01. Found: C, 72.64; H, 7.12. ^1^H-NMR (CDCl_3_, 500 MHz): *δ* 1.02 (s, 3H, 18-H_3_), 1.32–1.41 (overlapping m, 2H, 7**α**-H and 14**α**-H), 1.48 (m, 1H, 8**β**-H), 1.58 (m, 1H, 11**β**-H), 1.70 (m, 1H, 12**α**-H), 1.78–1.86 (overlapping m, 2H, 7**β**-H and 15**β**-H), 2.01 (m, 1H, 12**β**-H), 2.08 (dd, 1H, *J* = 12.8, *J* = 5.8 Hz), 2.16 (s, 3H, 3′-CH_3_), 2.22 (m, 1H, 9**α**-H), 2.39 (m, 1H, 11**α**-H), 2.83 (m, 2H, 6-H_2_), 3.37 (d, 1H, *J* = 9.4 Hz, 17**β**-H), 3.77 (s, 3H, 3-OMe), 4.65 (dd, 1H, *J* = 9.4 Hz, *J* = 6.4 Hz, 16**β**-H), 6.62 (d, 1H, *J* = 2.1 Hz, 4-H), 6.72 (dd, 1H, *J* = 8.6 Hz, *J* = 2.1 Hz, 2-H), 6.94 (d, 2H, *J* = 8.9 Hz, 2″-H and 6″-H), 7.19 (d, 1H, *J* = 8.6 Hz, 1-H), 8.14 (d, 2H, *J* = 8.5 Hz, 3″-H and 5″-H); ^13^C-NMR (CDCl_3_, 125 MHz): *δ* 17.1 (3′-CH_3_), 21.3 (C-18), 26.3 (C-11), 28.0 (C-7), 29.6 (C-6), 33.1 (C-15), 35.9 (C-12), 38.5 (C-8), 43.2 (C-9), 46.9 (C-13), 49.5 (C-14), 55.2 (3-OMe), 63.1 (C-16), 66.8 (C-17), 110.7 (2C, C-2″ and C-6″), 111.6 (C-2), 113.8 (C-4), 126.1 (3C, C-1, C-3″ and C-5″), 131.8 (C-10), 137.7 (C-5), 138.2 (C-4″), 148.2 (C-1″), 154.3 (C-3′), 157.6 (C-3); ESI-MS 446 [M + H]^+^.

*(16R,17S)-3-Methoxy-3′-methyl-1′-(4″-methoxyphenyl)-2′-pyrazolino[4′,5′:17,16]estra-1,3,5(10)-triene* (**17j**). According to the general procedure, 4-methoxyphenylhydrazine hydrochloride (**15j**, 192 mg) was used. Yield: 282 mg (white solid); Mp 210–212 °C; *R*_f_ = 0.58 (EtOAc/CH_2_Cl_2_ = 2:98). Anal. Calcd. for C_28_H_34_N_2_O_2_ (430.59): C, 78.10; H, 7.96. Found: C, 78.25; H, 7.82. ^1^H-NMR (CDCl_3_, 500 MHz): *δ* 0.98 (s, 3H, 18-H_3_), 1.38 (m, 1H, 7**α**-H), 1.43–1.51 (overlapping m, 2H, 8**β**-H and 14**α**-H), 1.56 (m, 1H, 11**β**-H), 1.67–1.77 (overlapping m, 2H, 12**α**-H and 15**β**-H), 1.84 (m, 1H, 7**β**-H), 1.97 (m, 1H, 12**β**-H), 2.09 (overlapping m, 1H, 15**α**-H), 2.11 (s, 3H, 3′-CH_3_), 2.23 (m, 1H, 9**α**-H), 2.38 (m, 1H, 11**α**-H), 2.83 (m, 2H, 6-H_2_), 3.26 (d, 1H, *J* = 9.8 Hz, 17**β**-H), 3.77 (s, 6H, 3-OMe and 4″-OMe), 4.49 (dd, 1H, *J* = 9.8 Hz, *J* = 5.5 Hz, 16**β**-H), 6.63 (d, 1H, *J* = 2.6 Hz, 4-H), 6.72 (dd, 1H, *J* = 8.5 Hz, *J* = 2.6 Hz, 2-H), 6.86 (d, 2H, *J* = 8.7 Hz, 3″-H and 5″-H), 6.97 (d, 2H, *J* = 8.7 Hz, 2″-H and 6″-H), 7.20 (d, 1H, *J* = 8.5 Hz, 1-H); ^13^C-NMR (CDCl_3_, 125 MHz): *δ* 17.0 (3′-CH_3_), 21.5 (C-18), 26.5 (C-11), 28.1 (C-7), 29.7 (C-6), 33.6 (C-15), 36.0 (C-12), 38.5 (C-8), 43.3 (C-9), 46.9 (C-13), 49.7 (C-14), 55.2 (3-OMe), 55.8 (4″-OMe), 65.2 (C-16), 66.5 (C-17), 111.5 (C-2), 113.1 (2C, C-2″ and C-6″), 113.7 (C-4), 114.7 (2C, C-3″ and C-5″), 126.2 (C-1), 132.3 (C-10), 137.9 (C-5), 139.8 (C-1″), 149.4 (C-3′), 152.5 (C-4″), 157.5 (C-3); ESI-MS 431 [M + H]^+^.

## 4. Conclusions

In summary, a number of novel ring d-fused five-membered *N*,*N*-heterocycles in the estrone series were efficiently prepared under MW conditions and their structures were completely characterized by 1D and 2D NMR measurements. In contrast to the related literature background, the ring-closure of 16-benzylidene estrone 3-methyl ether with hydrazine hydrate in AcOH led to a 2:1 diastereomeric mixture of 1,3-disubstituted 16β,17β-*cis*- and 16α,17α-*cis*-fused products via tautomerization of the initially formed 1-unsubstituted Δ^2^-pyrazolines and subsequent acetylation under the applied conditions. However, a mestranol-derived α,β-enone was also synthesized by a two-step E2-type elimination/acid-catalyzed Markovnikov hydration protocol instead of performing the process in a single step under Rupe conditions in order to avoid carbocation-mediated side reactions. The conversion of this latter compound with different arylhydrazines proved to be highly diastereoselective to furnish novel 16α,17α-*cis*-annelated heterocycles in good to excellent yields independently of the substituents of the reagents applied. The resulting pyrazolines were found to be extremely resistant to oxidation and may deserve attention from a pharmacological point of view.

## Figures and Tables

**Table 1:** Stereoselective syntheses of ring d-fused 1′-aryl-2′-pyrazolines in the estrone series.

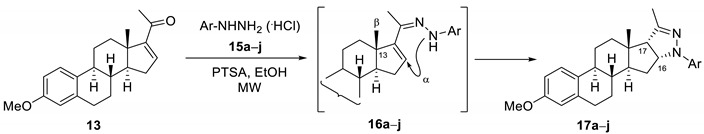				
Entry	Ar-NH-NH_2_	Ar	Product	Yield ^1^ (%)
1	**15a**	Ph	**17a**	89
2	**15b**	4-CH_3_-C_6_H_4_	**17b**	95
3	**15c**	2-CH_3_-C_6_H_4_	**17c**	75
4	**15d**	2,4-diCH_3_-C_6_H_3_	**17d**	80
5	**15e**	4-F-C_6_H_4_	**17e**	88
6	**15f**	4-Cl-C_6_H_4_	**17f**	81
7	**15g**	4-Br-C_6_H_4_	**17g**	83
8	**15h**	4-CN-C_6_H_4_	**17h**	85
9	**15i**	4-NO_2_-C_6_H_4_	**17i**	80
10	**15j**	4-MeO-C_6_H_4_	**17j**	82

^1^ After purification by column chromatography.

## Supplementary Materials

Spectral data of the synthesized compounds are available online: ^1^H-NMR, ^13^C-NMR, and 2D NMR.
