# Supplementary material for: Microwave-Assisted Stereoselective Heterocyclization to Novel Ring d-fused Arylpyrazolines in the Estrone Series
Source: Molecules. 2019 Feb 4;24(3):569. doi: 10.3390/molecules24030569 (PMC6384934; doi:10.3390/molecules24030569)

Supplementary material

for

Microwave-assisted stereoselective heterocyclization to novel ring D-fused arylpyrazolines in the estrone series

Gergő Mótyán, Barnabás Molnár, János Wölfling, Éva Frank

Department of Organic Chemistry, University of Szeged, Dóm tér 8, H-6720 Szeged, Hungary

*Corresponding author. E-mail address: frank@chem.u-szeged.hu

Table of Contents

Spectral data (^{1}H NMR, ^{13}C NMR, and 2D NMR) of the synthesized compounds. S2-S22

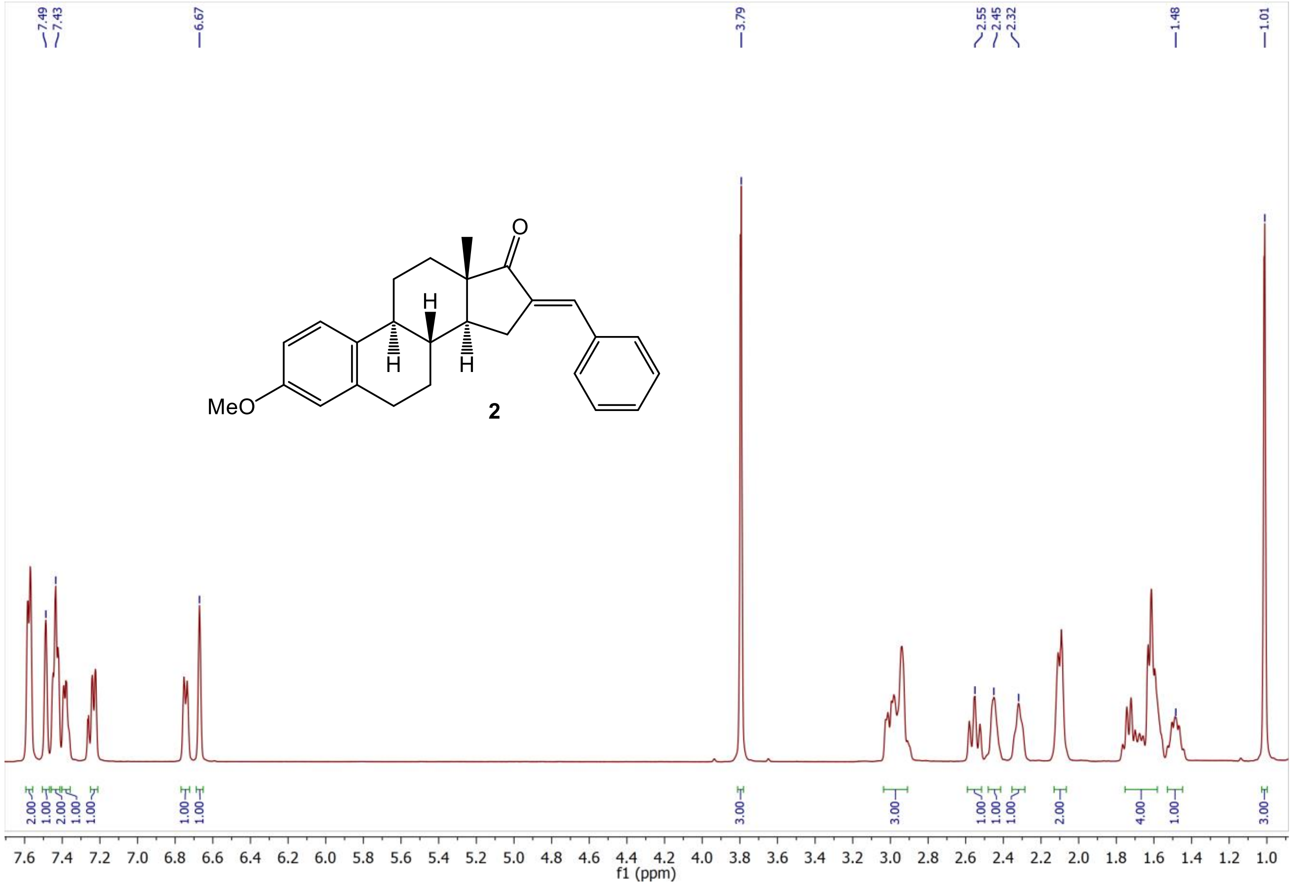
7.49
7.43
6.67
3.79
2.55
2.45
2.32
1.48
1.01
MeO
2
2.00
1.00
2.00
1.00
1.00
1.00
1.00
3.00
3.00
1.00
1.00
1.00
2.00
4.00
1.00
3.00
f1 (ppm)

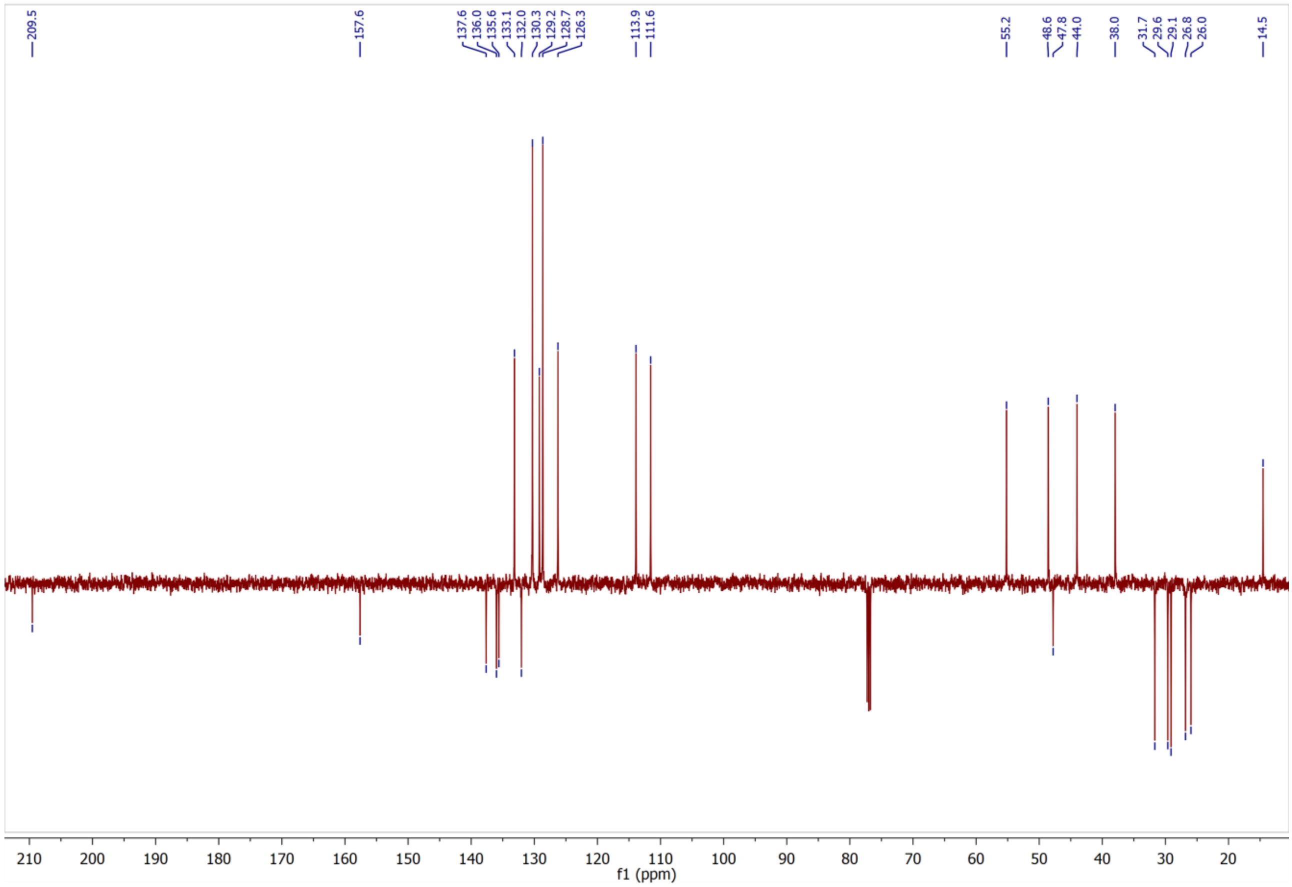
209.5
157.6
137.6
136.0
135.6
133.1
132.0
130.3
129.2
128.7
126.3
113.9
111.6
55.2
48.6
47.8
44.0
38.0
31.7
29.6
29.1
26.8
26.0
14.5
f1 (ppm)

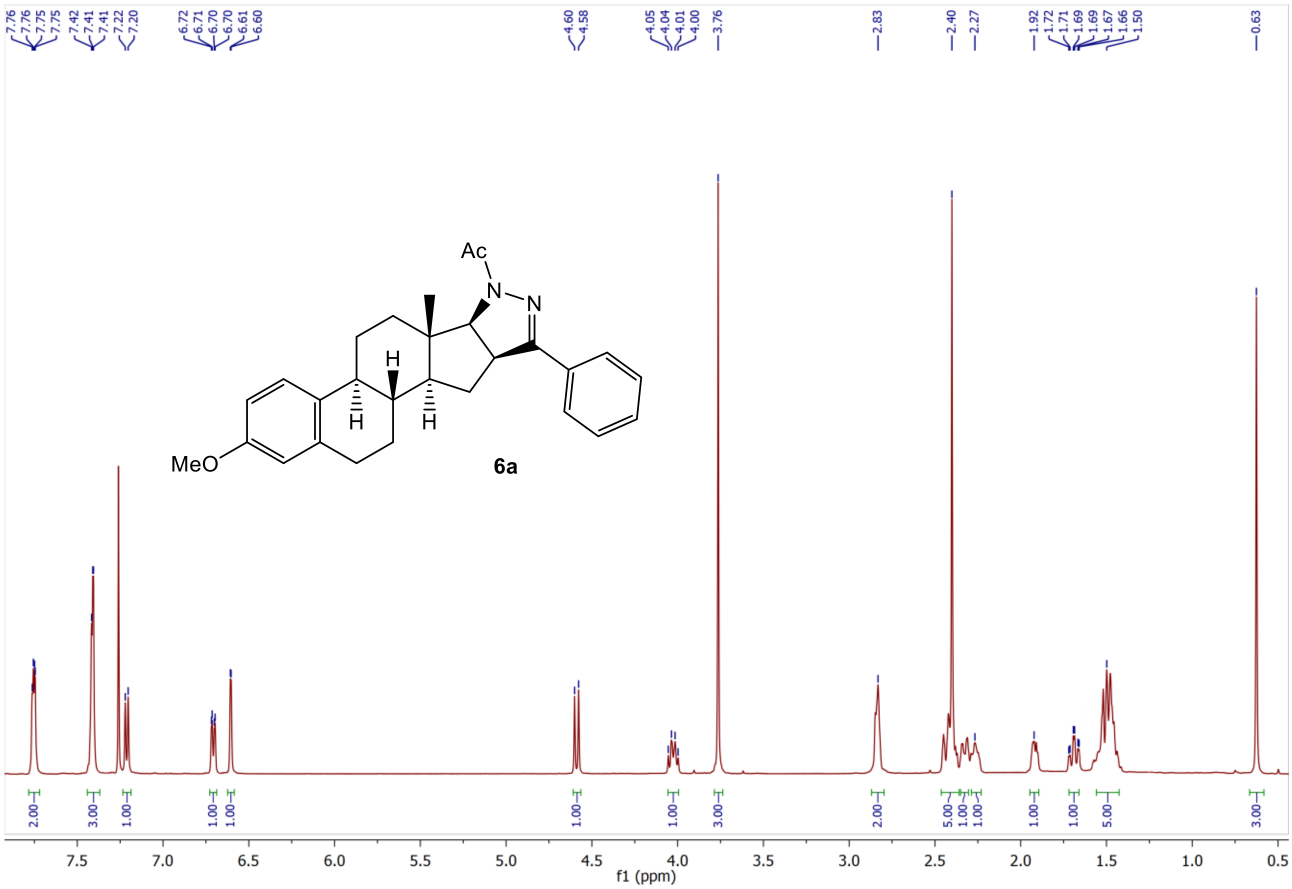
7.76
7.76
7.75
7.75
7.42
7.41
7.41
7.22
7.20
6.72
6.71
6.70
6.70
6.61
6.60
4.60
4.58
4.05
4.04
4.01
4.00
3.76
2.83
2.40
2.27
1.92
1.72
1.71
1.69
1.69
1.67
1.66
1.50
0.63
Ac
N
N
H
H
H
MeO
6a
f1 (ppm)

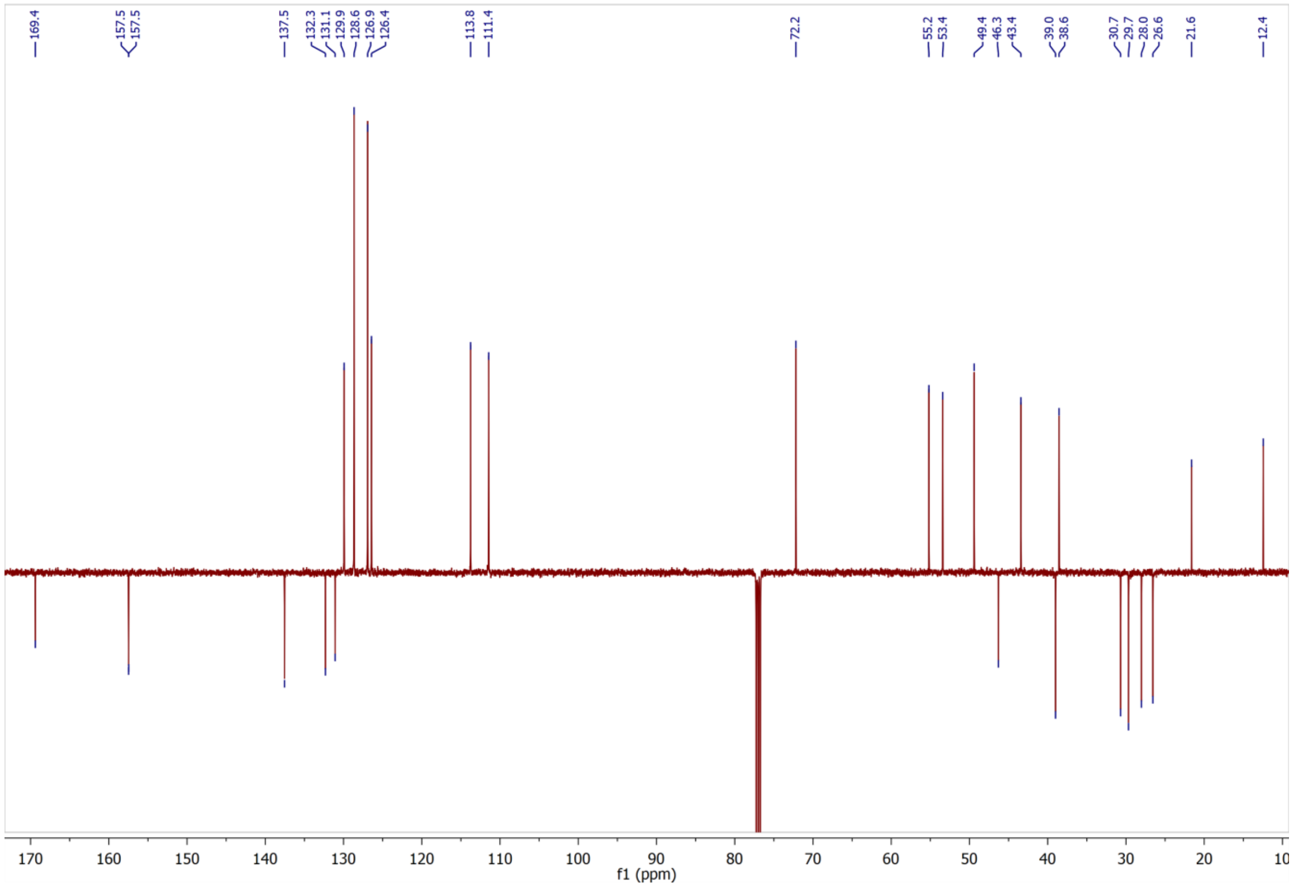
169.4
157.5
157.5
137.5
132.3
131.1
129.9
128.6
126.9
126.4
113.8
111.4
72.2
55.2
53.4
49.4
46.3
43.4
39.0
38.6
30.7
29.7
28.0
26.6
21.6
12.4
f1 (ppm)

HSQC spectrum of **6a**

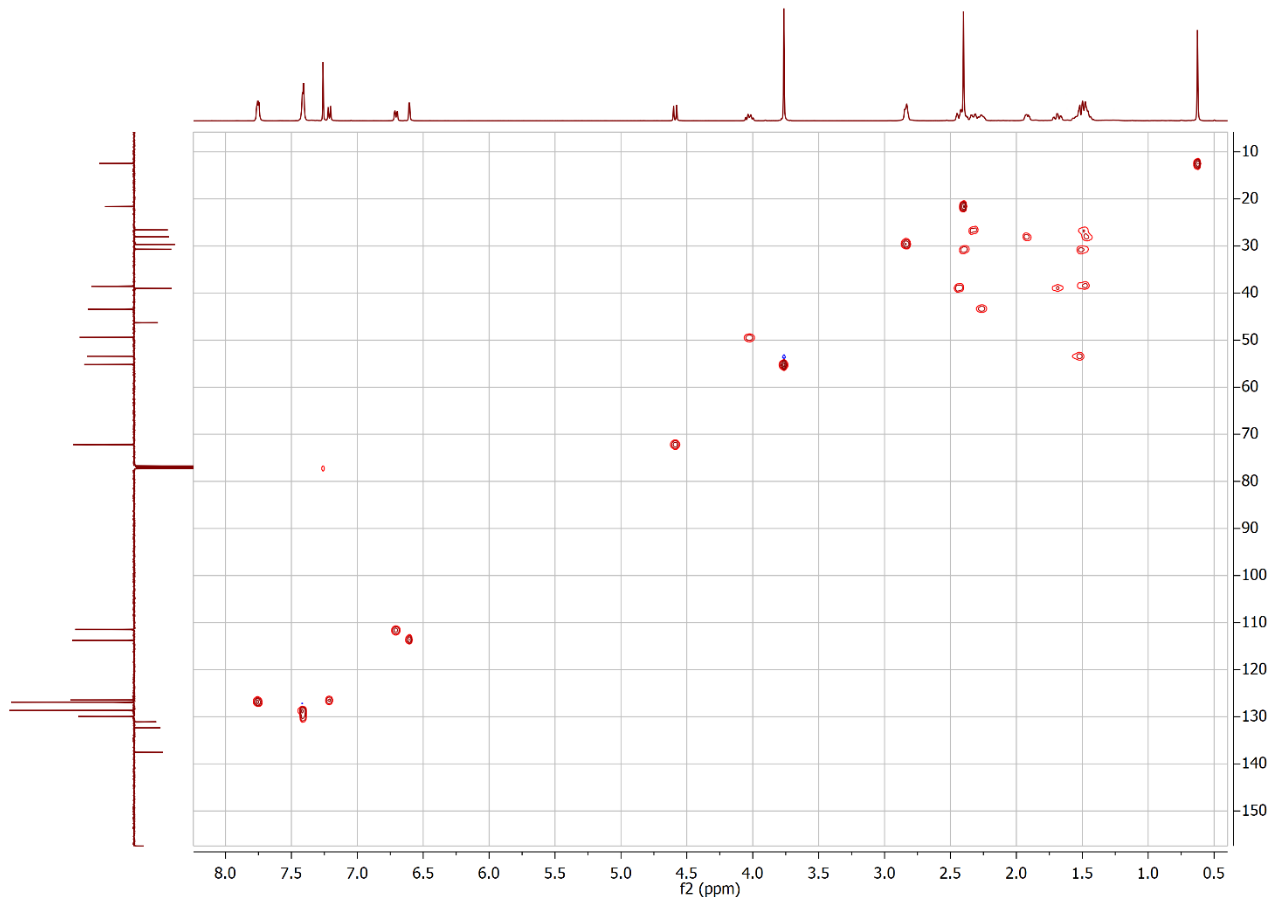

HMBC spectrum of **6a**

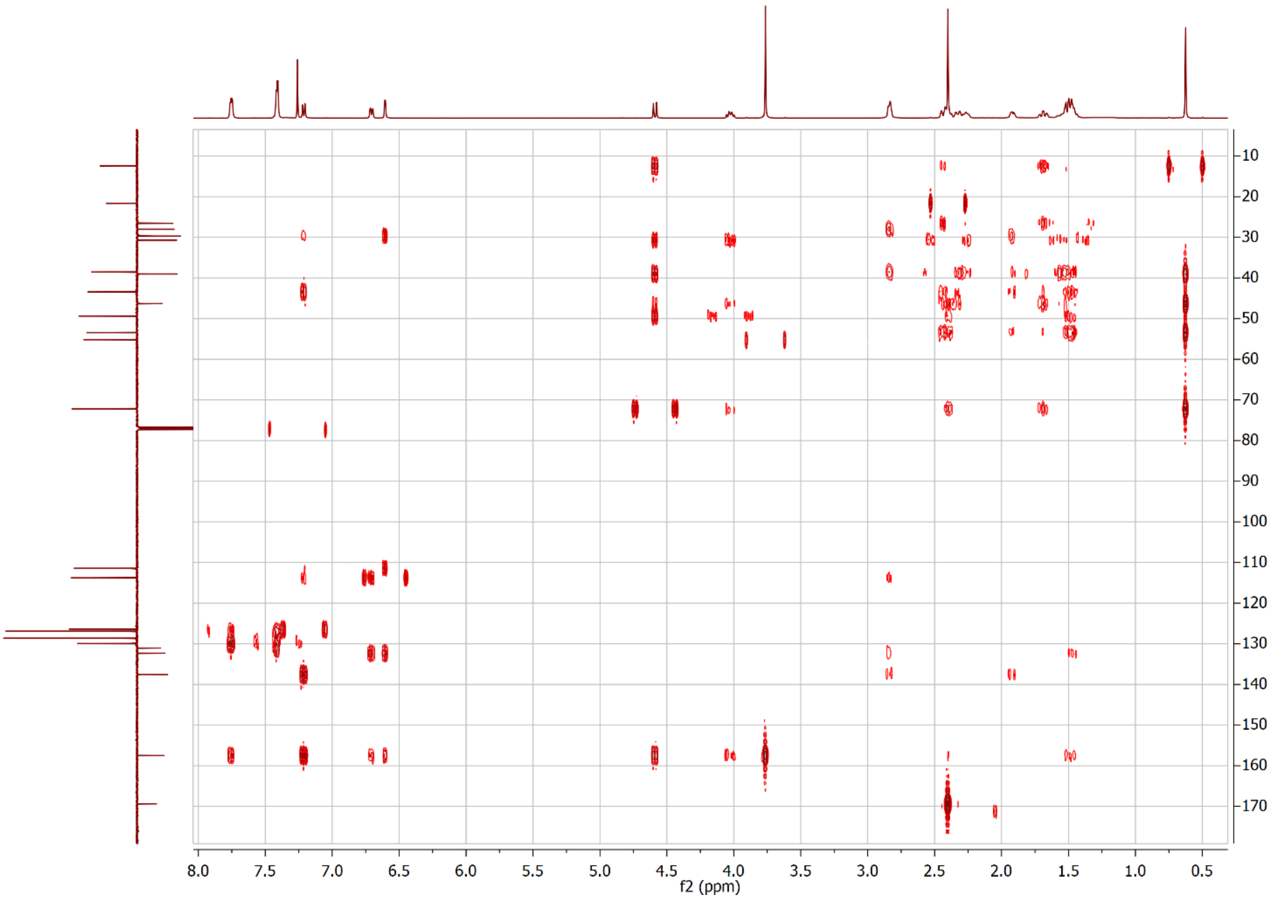

NOESY spectrum of **6a**

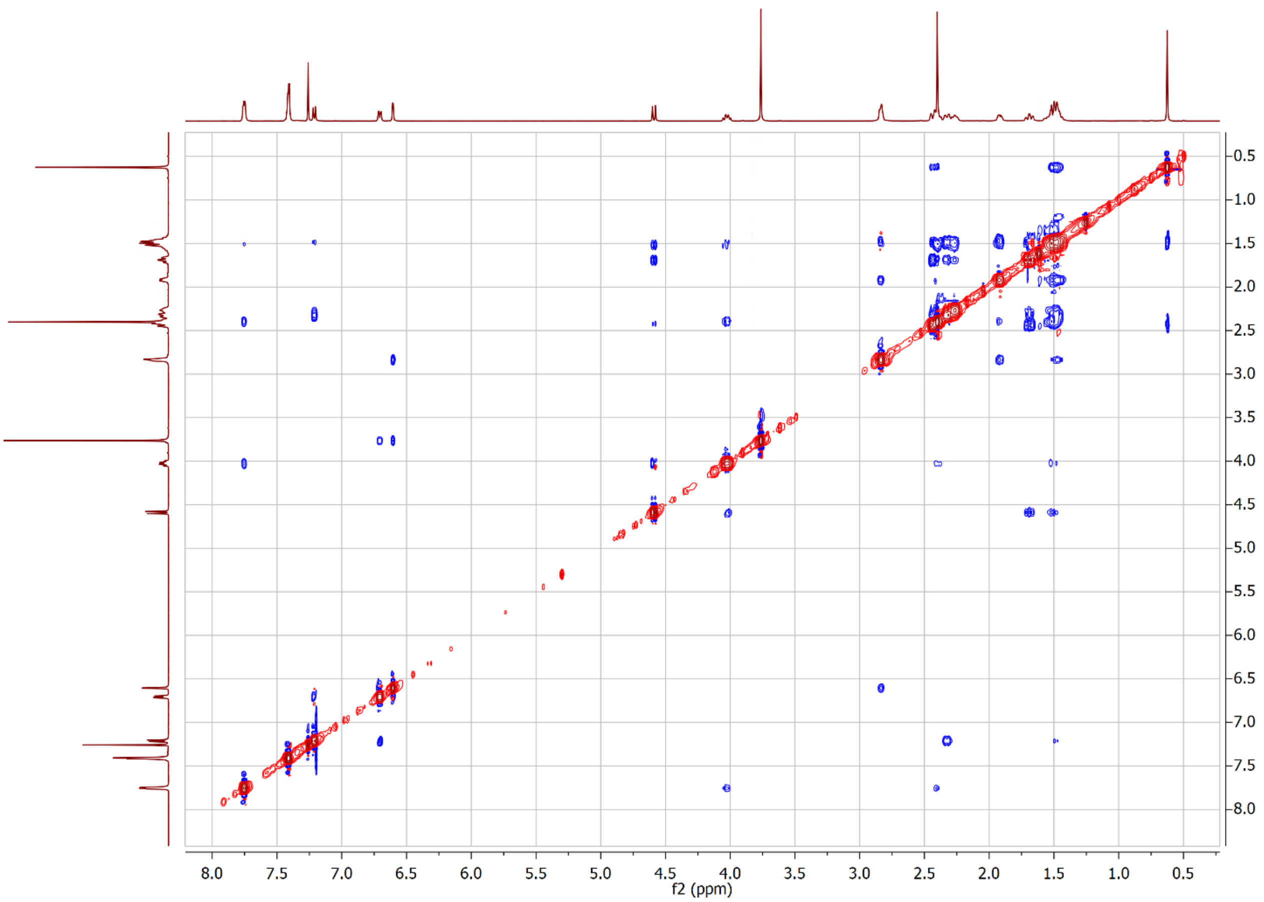

COSY spectrum of **6a**

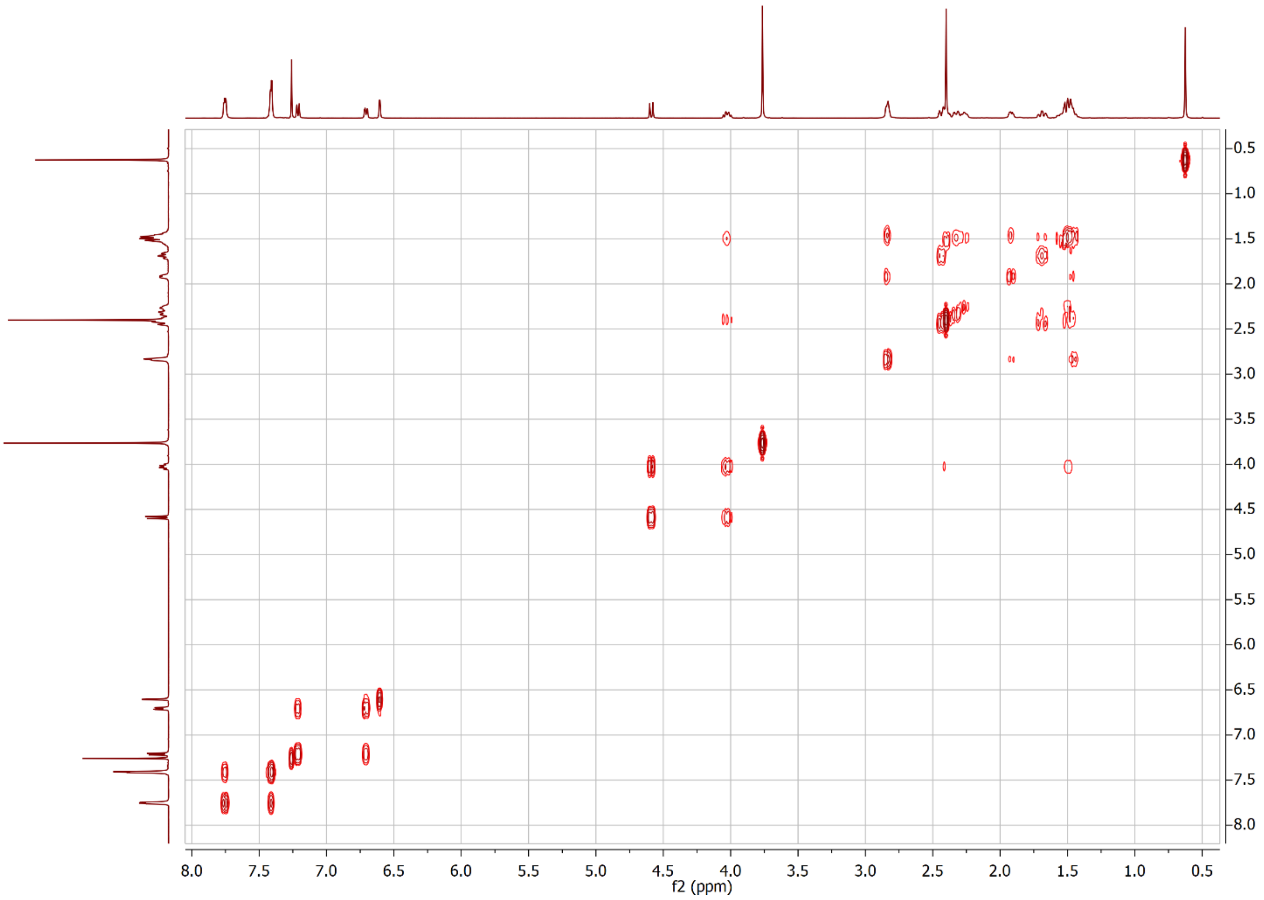

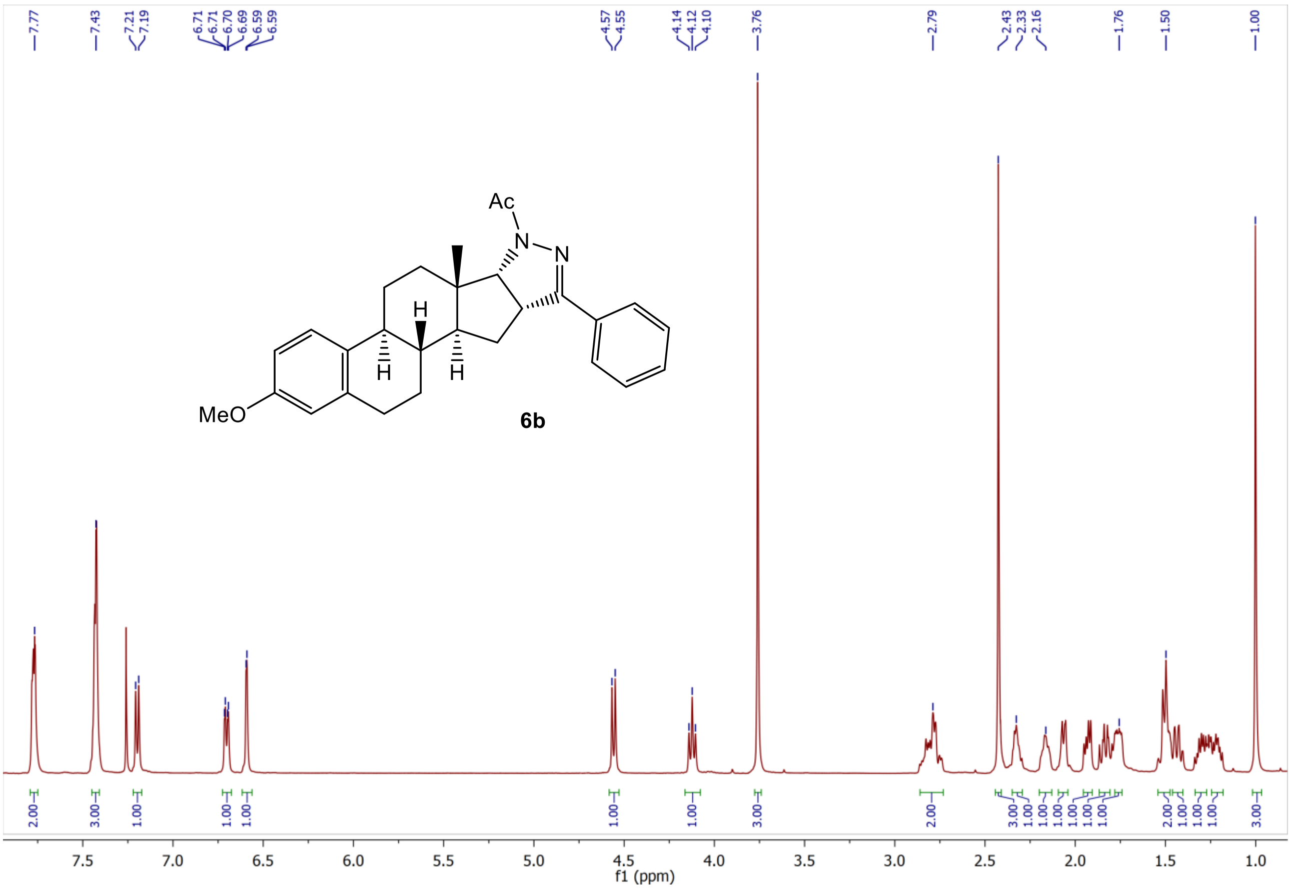

7.77
7.43
7.21
7.19
6.71
6.71
6.70
6.69
6.59
6.59
4.57
4.55
4.14
4.12
4.10
3.76
2.79
2.43
2.33
2.16
1.76
1.50
1.00
Ac
N
N
H
H
H
MeO
f1 (ppm)

6b

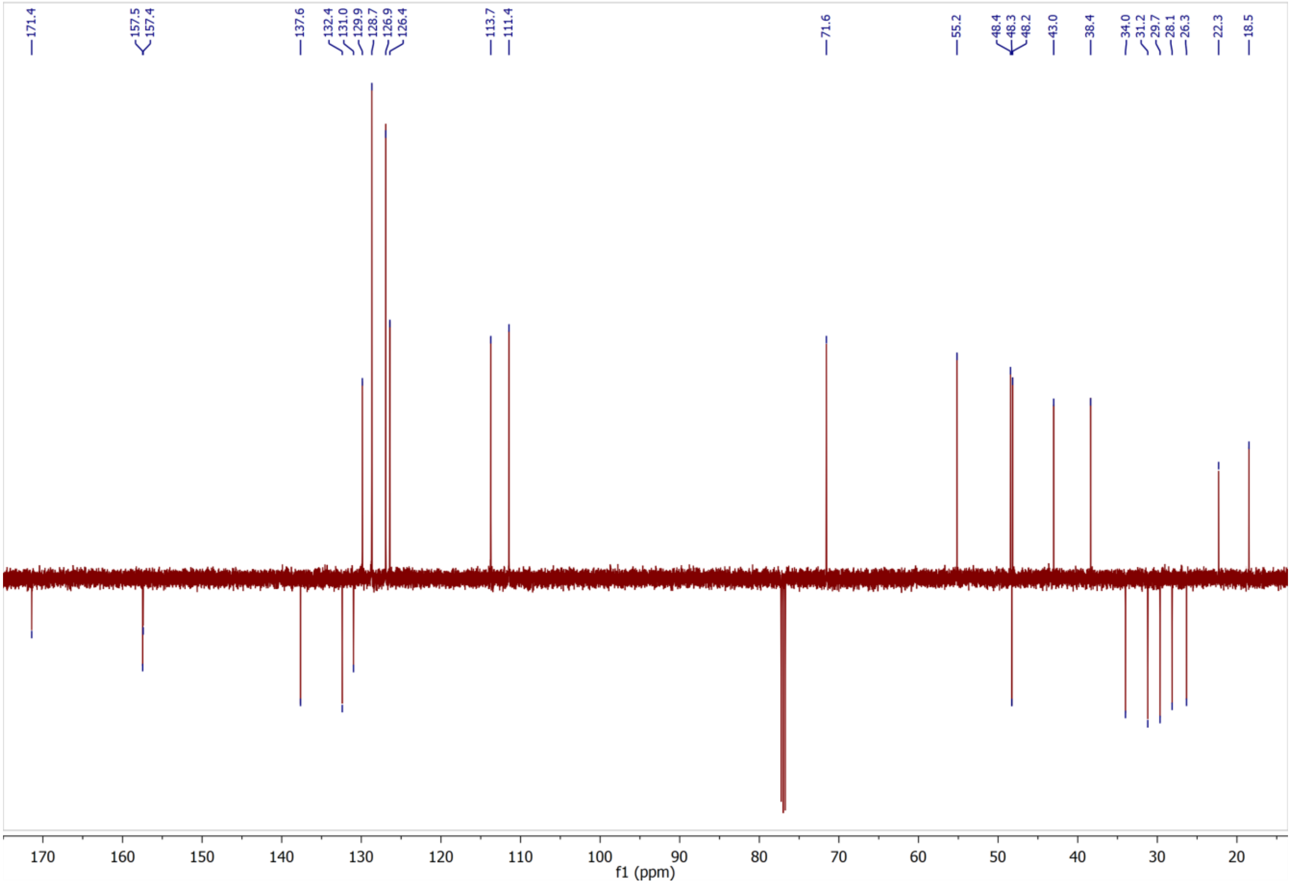

171.4
157.5
157.4
137.6
132.4
131.0
129.9
128.7
126.9
126.4
113.7
111.4
71.6
55.2
48.4
48.3
48.2
43.0
38.4
34.0
31.2
29.7
28.1
26.3
22.3
18.5
f1 (ppm)

HSQC spectrum of **6b**

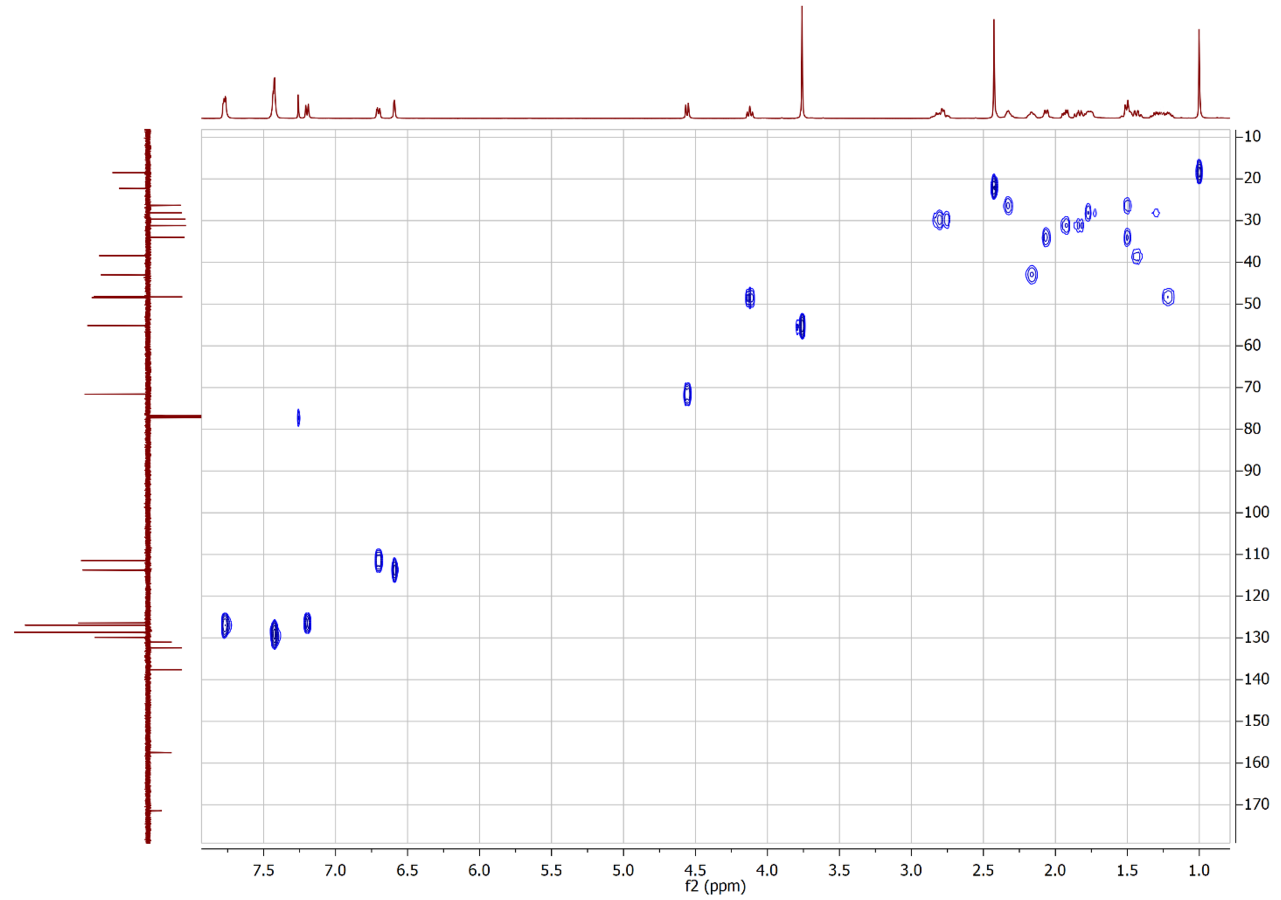

HMBC spectrum of **6b**

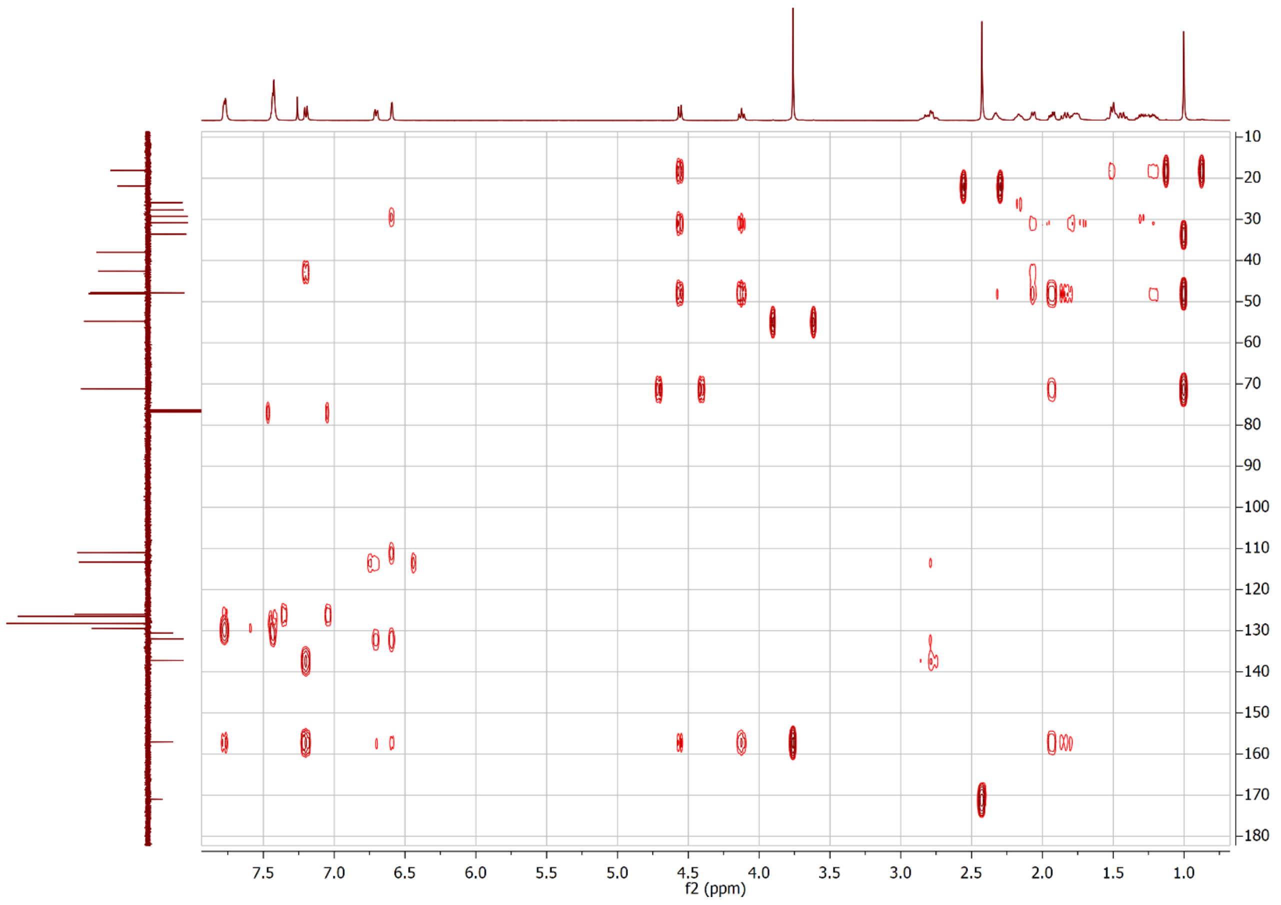

NOESY spectrum of **6b**

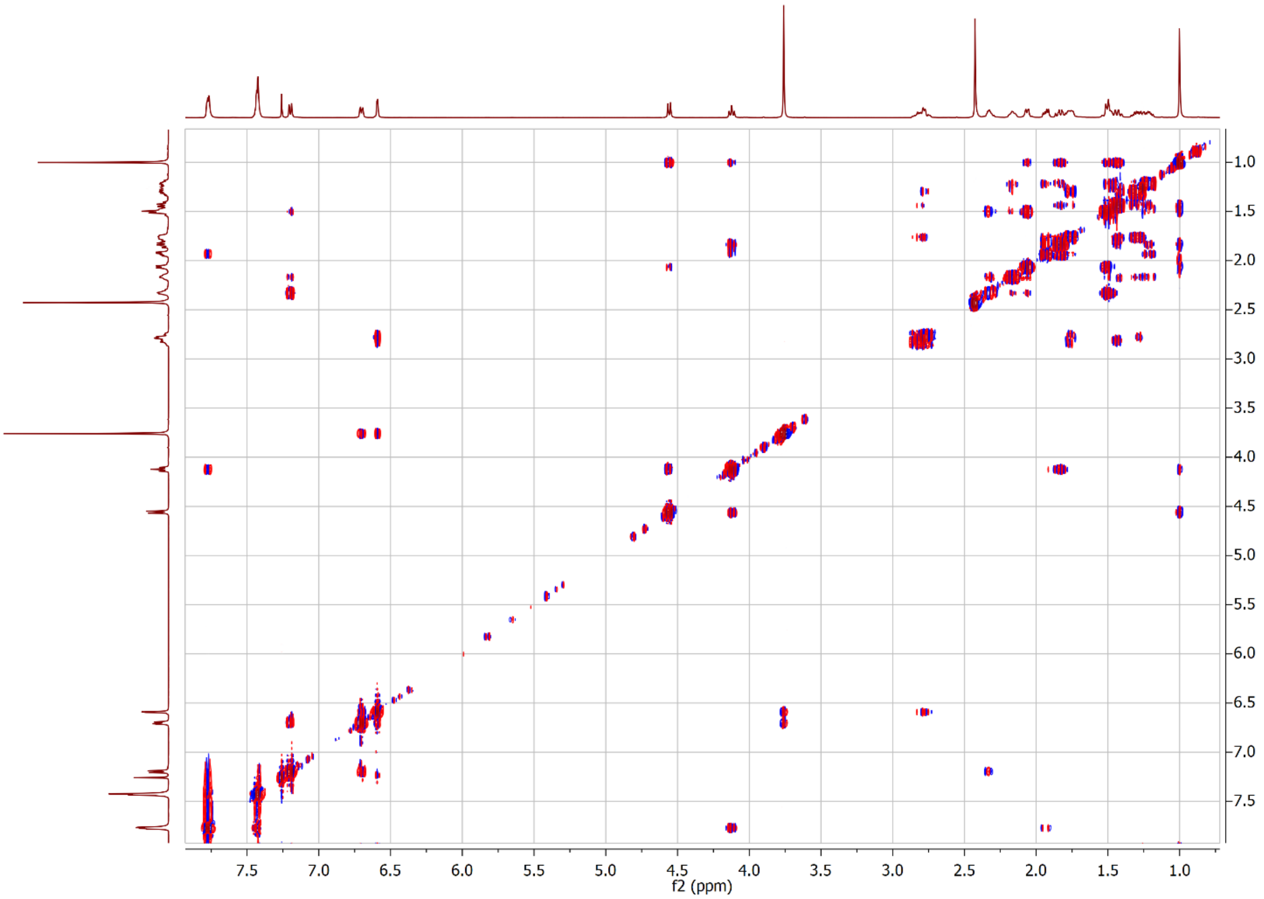

COSY spectrum of **6b**

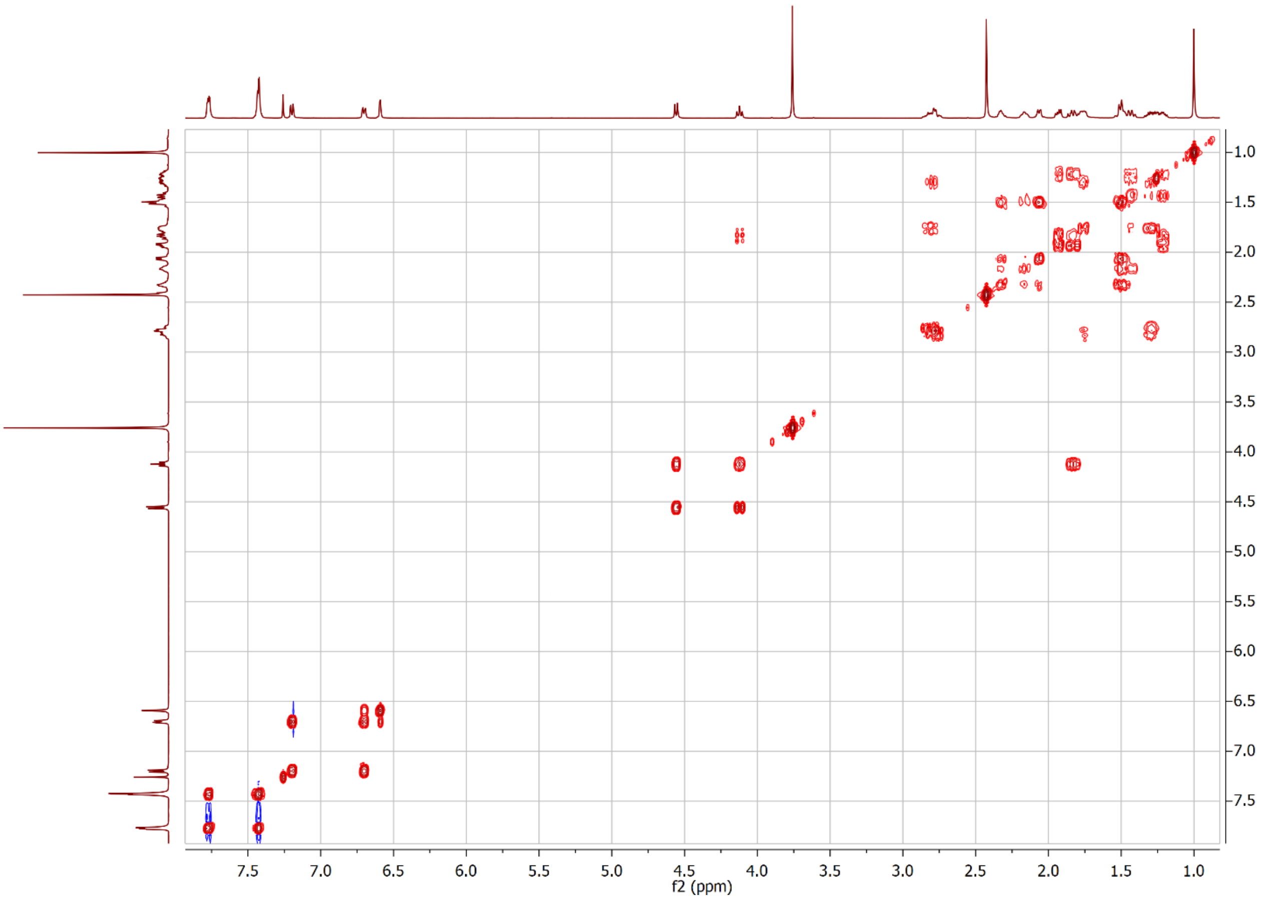

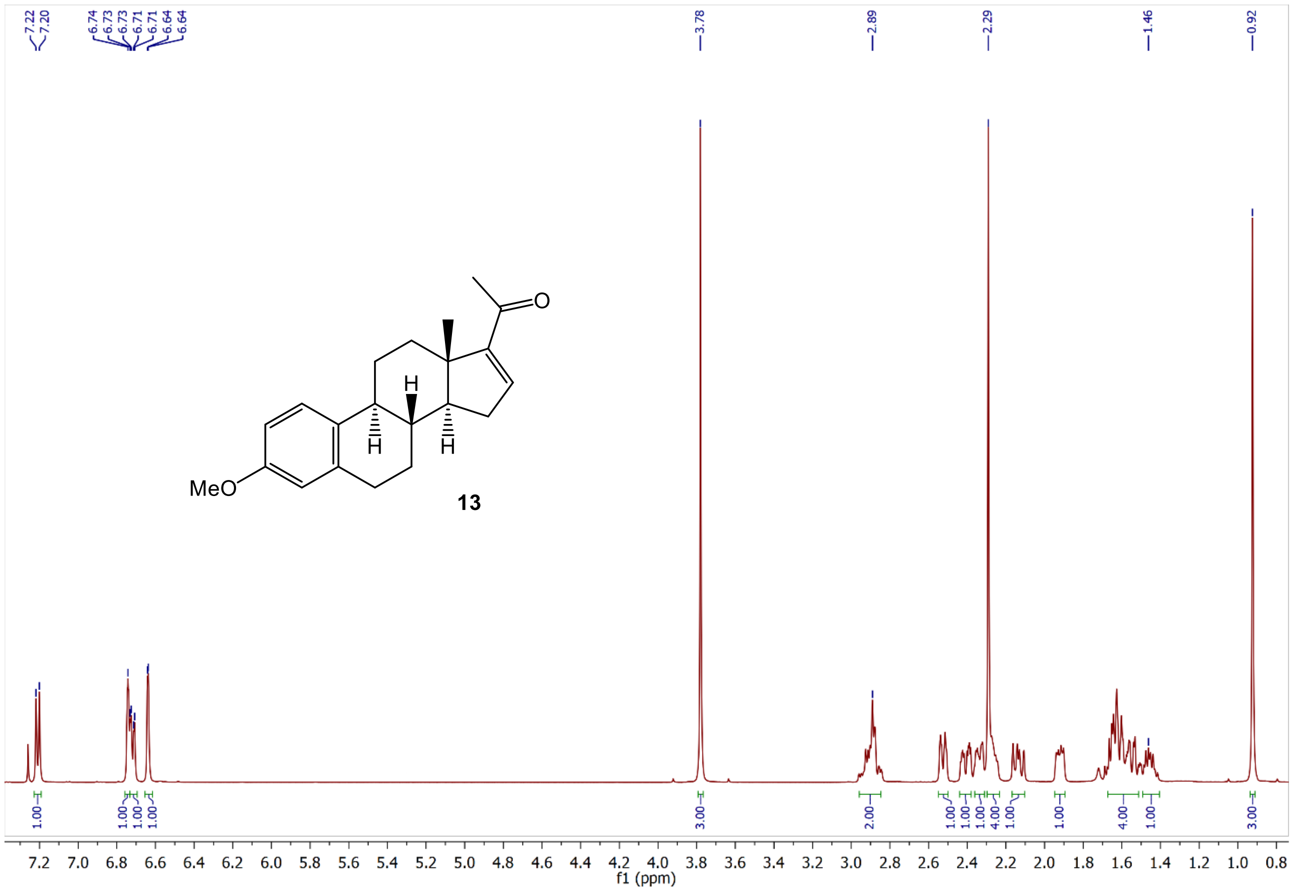
7.22
7.20
6.74
6.73
6.73
6.71
6.71
6.64
6.64
3.78
2.89
2.29
1.46
0.92
MeO
H
H
H
O
13
1.00
1.00
1.00
1.00
3.00
2.00
1.00
1.00
1.00
4.00
1.00
1.00
4.00
1.00
3.00
7.2 7.0 6.8 6.6 6.4 6.2 6.0 5.8 5.6 5.4 5.2 5.0 4.8 4.6 4.4 4.2 4.0 3.8 3.6 3.4 3.2 3.0 2.8 2.6 2.4 2.2 2.0 1.8 1.6 1.4 1.2 1.0 0.8
f1 (ppm)

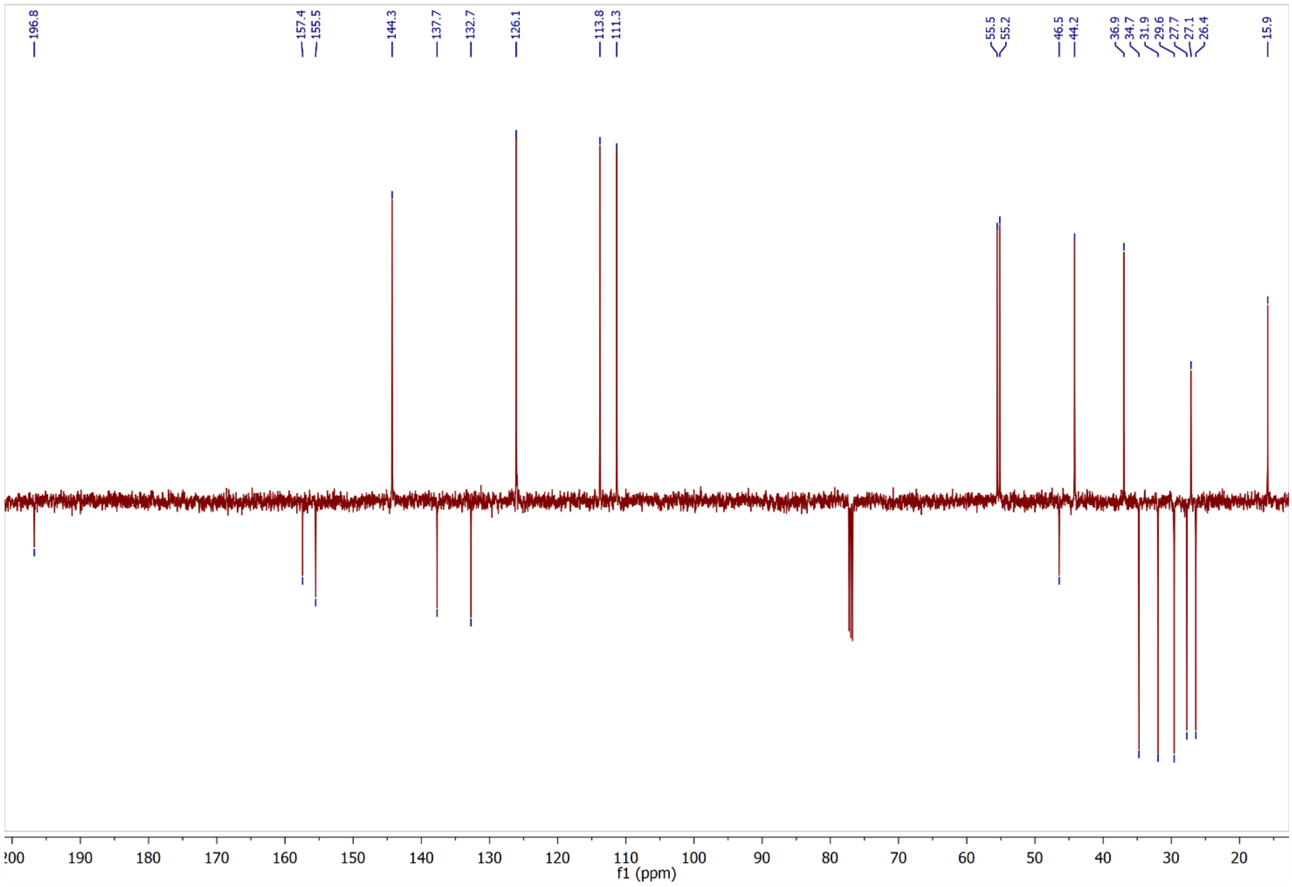
196.8
157.4
155.5
144.3
137.7
132.7
126.1
113.8
111.3
55.5
55.2
46.5
44.2
36.9
34.7
31.9
29.6
27.7
27.1
26.4
15.9
200 190 180 170 160 150 140 130 120 110 100 90 80 70 60 50 40 30 20
f1 (ppm)

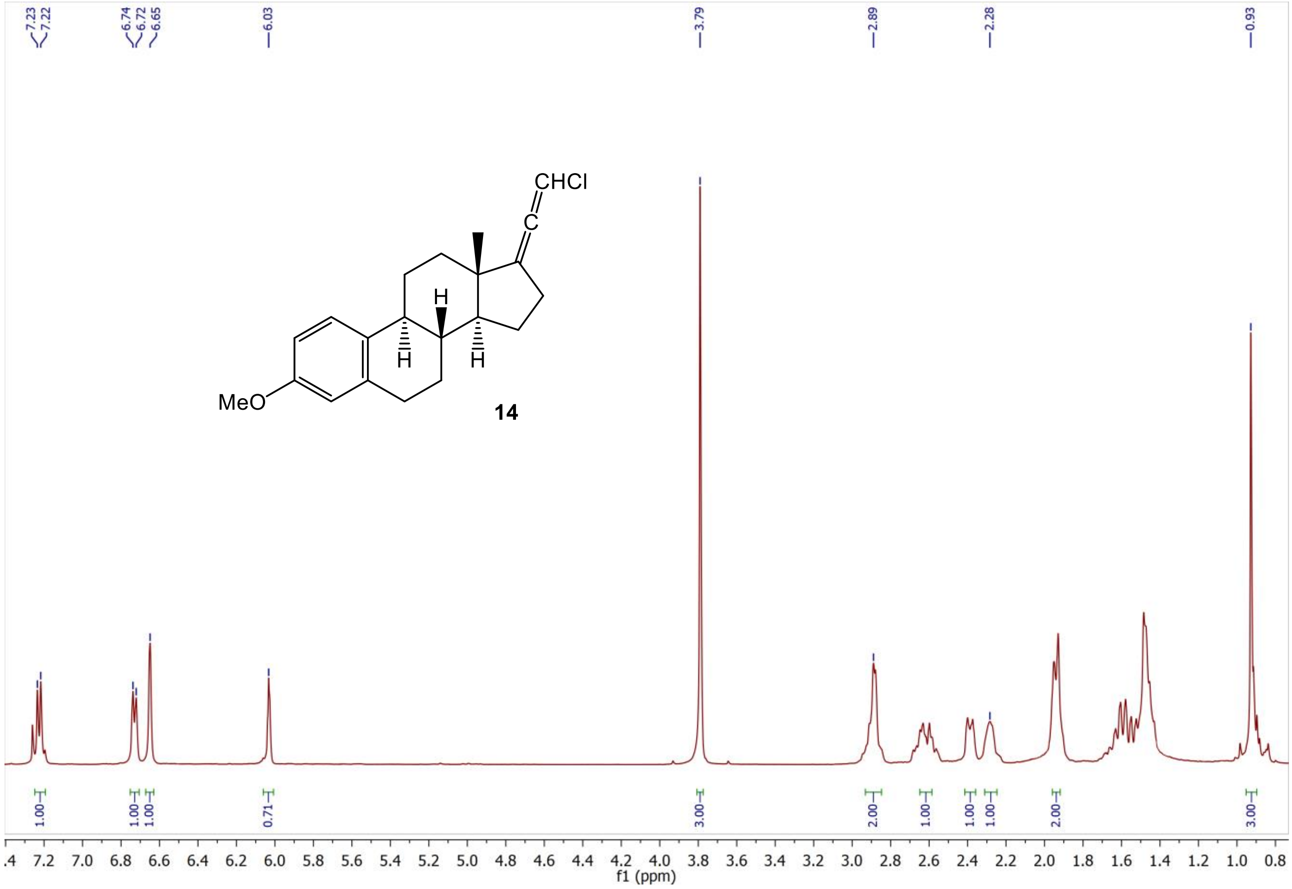
7.23
7.22
6.74
6.72
6.65
6.03
3.79
2.89
2.28
0.93
CHCl
C
H
H
H
MeO
14
1.00
1.00
1.00
0.71
3.00
2.00
1.00
1.00
1.00
2.00
3.00
f1 (ppm)

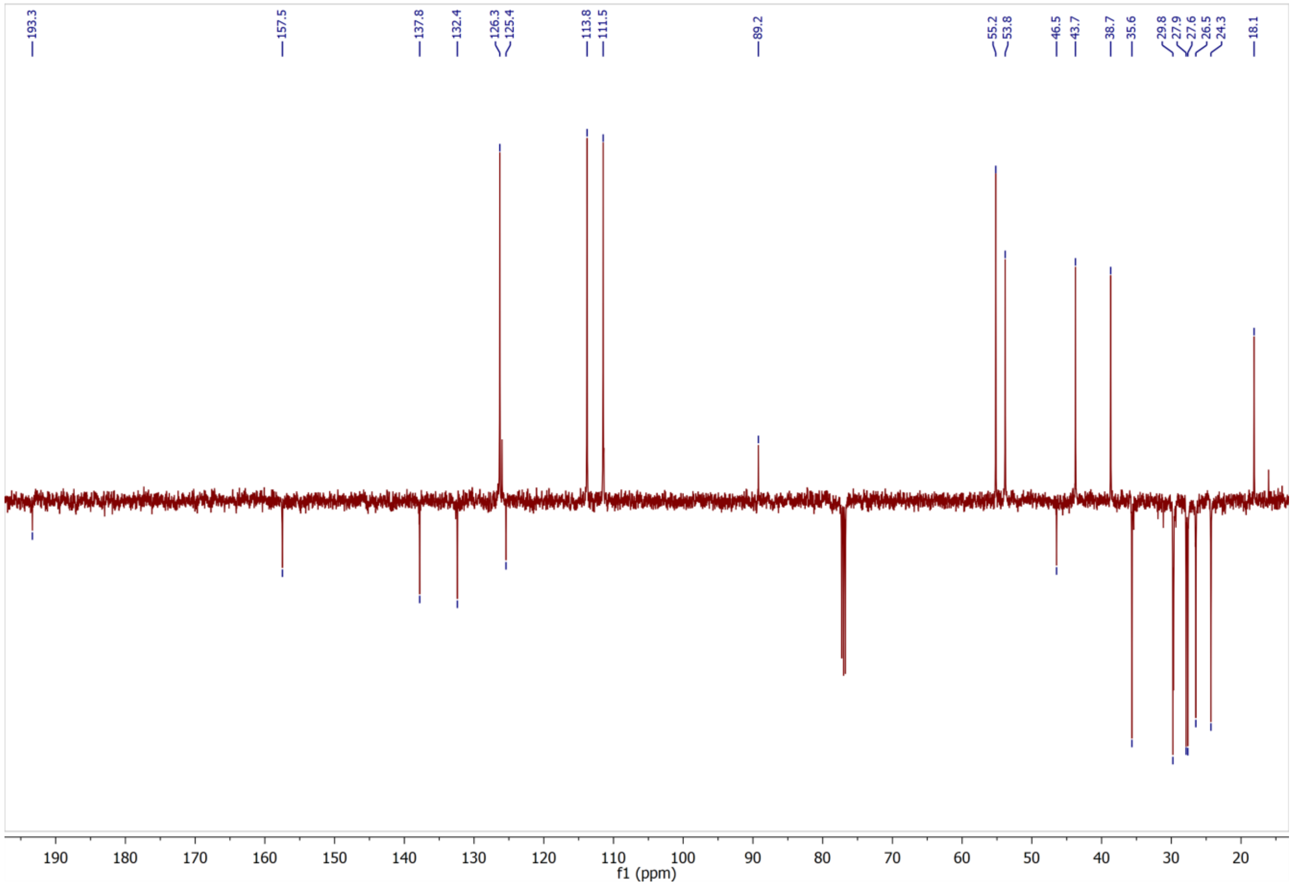
193.3
157.5
137.8
132.4
126.3
125.4
113.8
111.5
89.2
55.2
53.8
46.5
43.7
38.7
35.6
29.8
27.9
27.6
26.5
24.3
18.1
f1 (ppm)

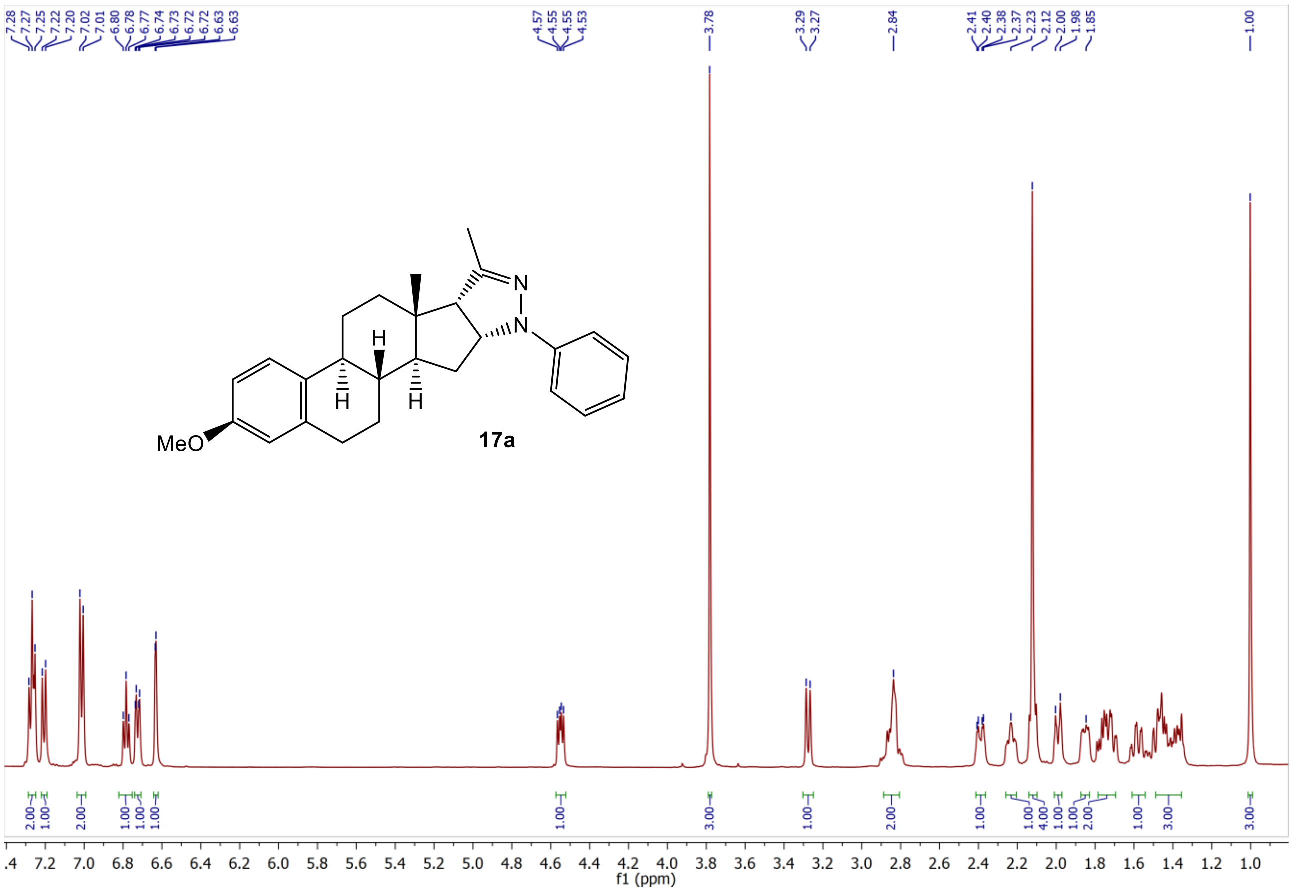
7.28
7.27
7.25
7.22
7.20
7.02
7.01
6.80
6.78
6.77
6.74
6.73
6.72
6.63
6.63
4.57
4.55
4.55
4.53
3.78
3.29
3.27
2.84
2.41
2.40
2.38
2.37
2.23
2.12
2.00
1.98
1.85
1.00
MeO
H
H
H
N
N
17a
2.00
1.00
2.00
1.00
1.00
1.00
1.00
3.00
1.00
2.00
1.00
1.00
4.00
1.00
1.00
2.00
1.00
3.00
3.00
f1 (ppm)

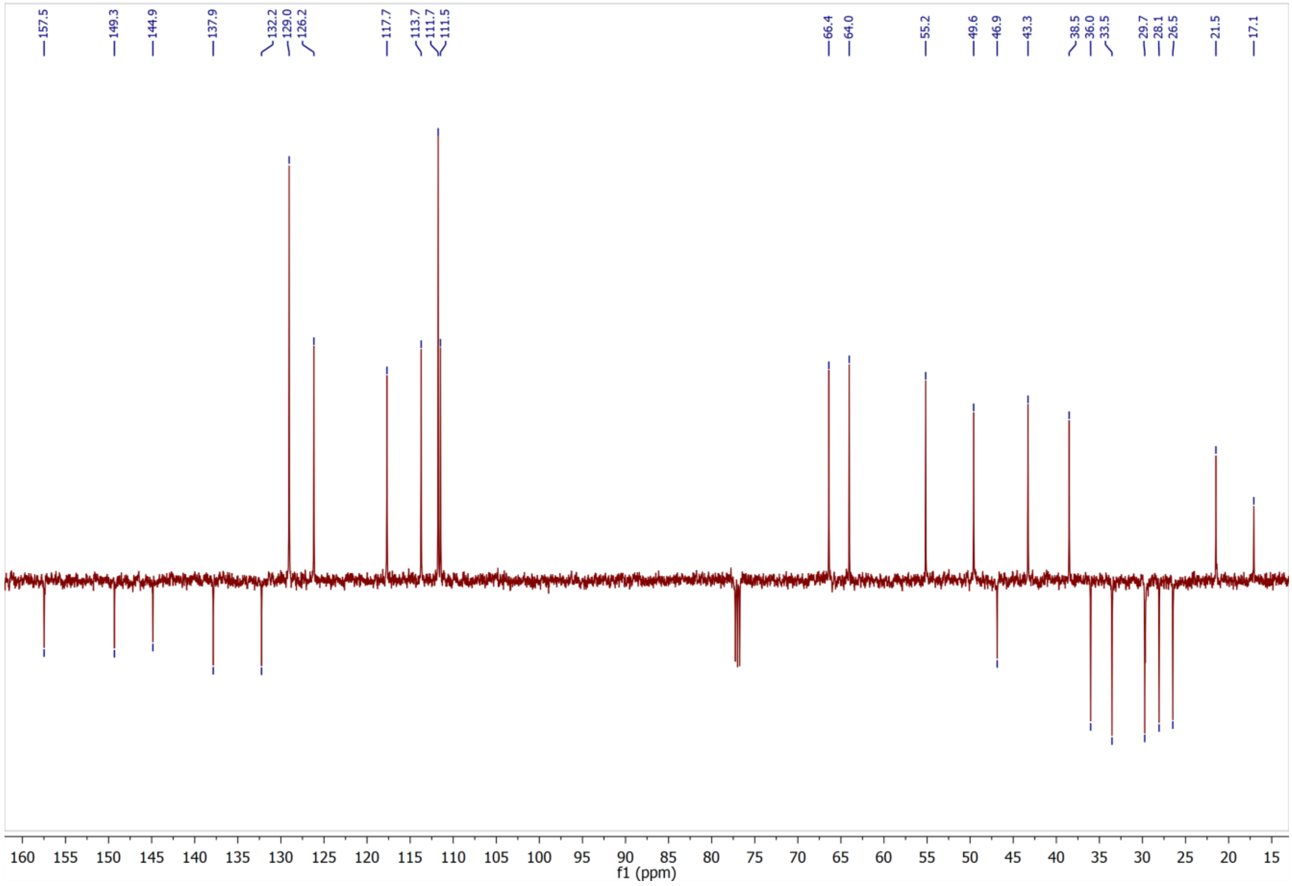
157.5
149.3
144.9
137.9
132.2
129.0
126.2
117.7
113.7
111.7
111.5
66.4
64.0
55.2
49.6
46.9
43.3
38.5
36.0
33.5
29.7
28.1
26.5
21.5
17.1
f1 (ppm)

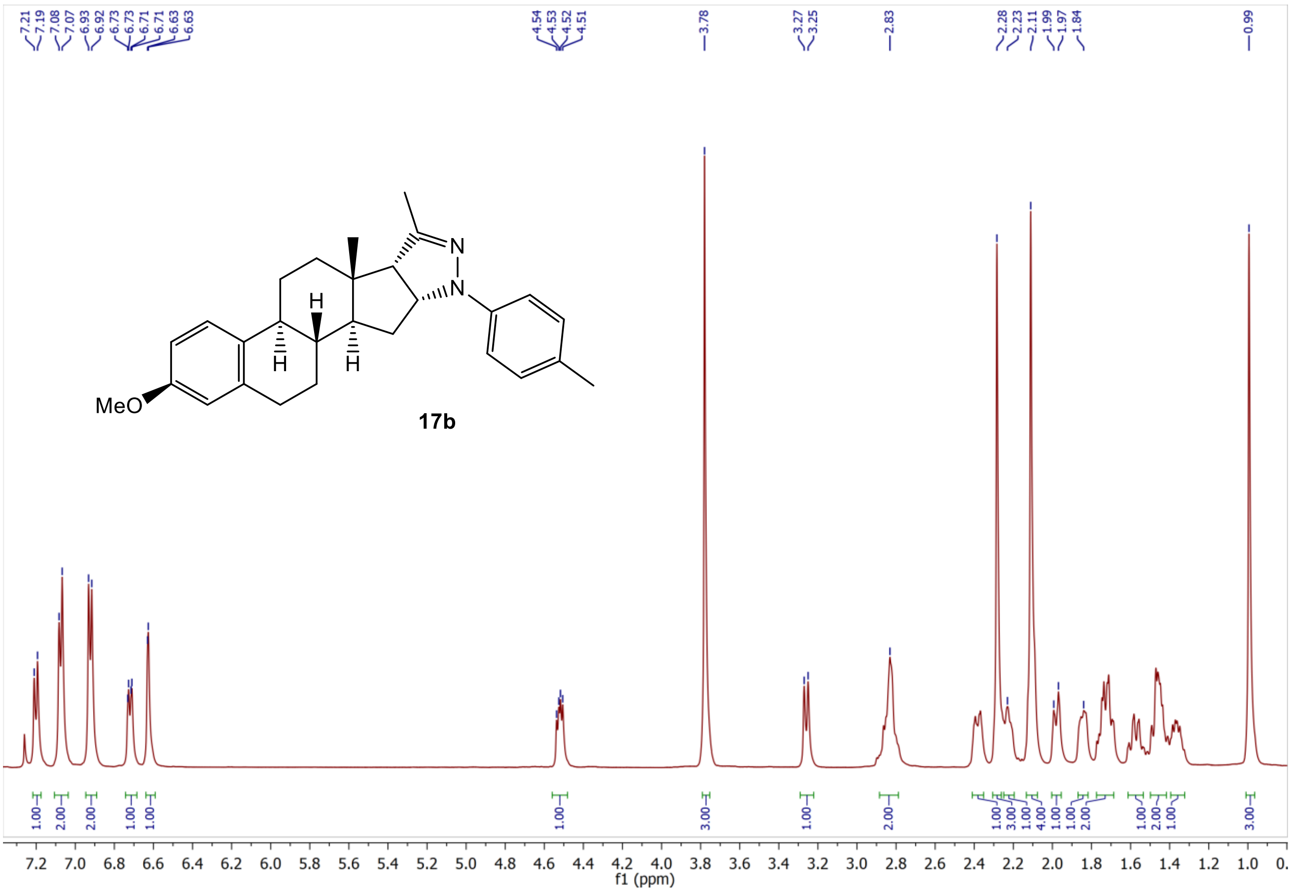
MeO
H
H
H
N
N
17b
f1 (ppm)

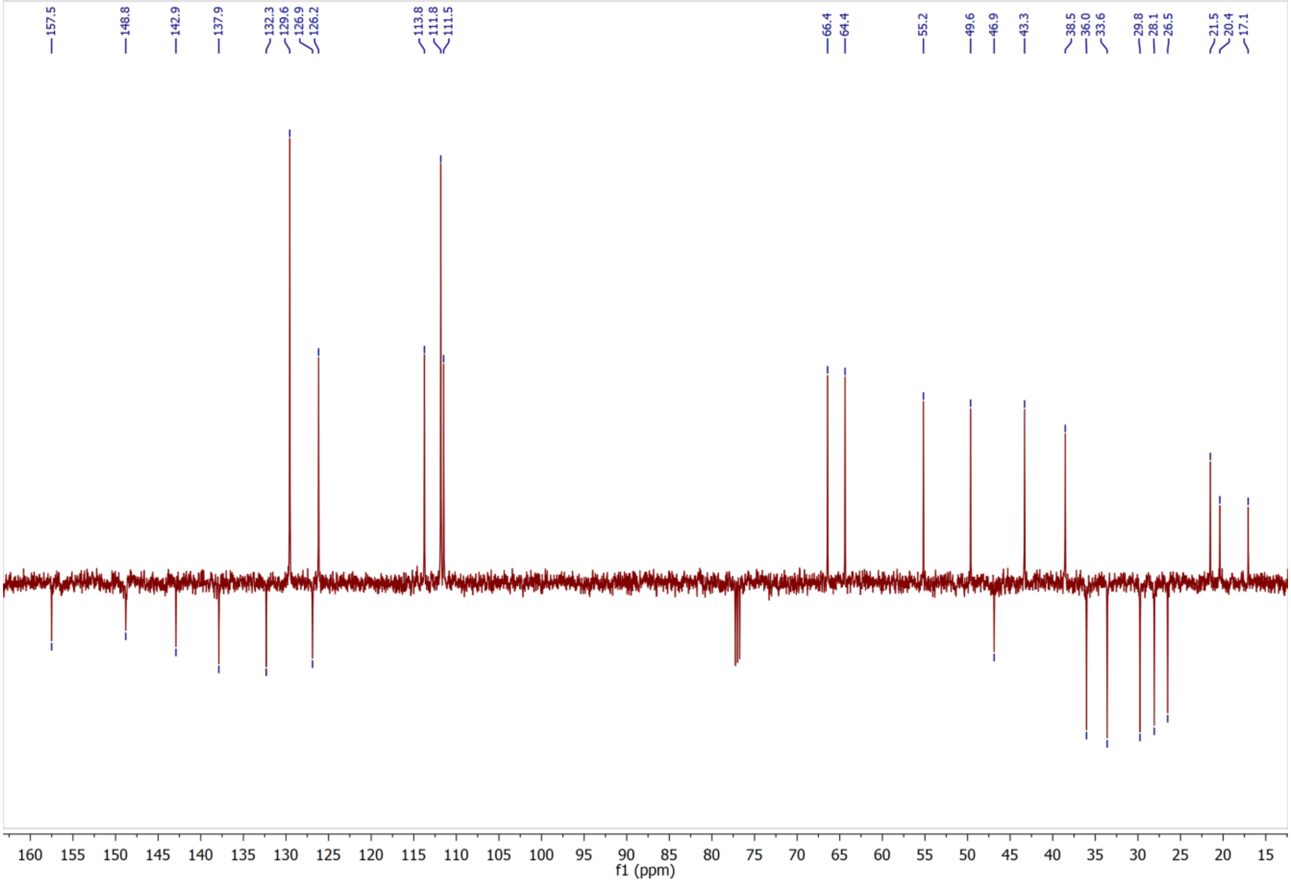
f1 (ppm)

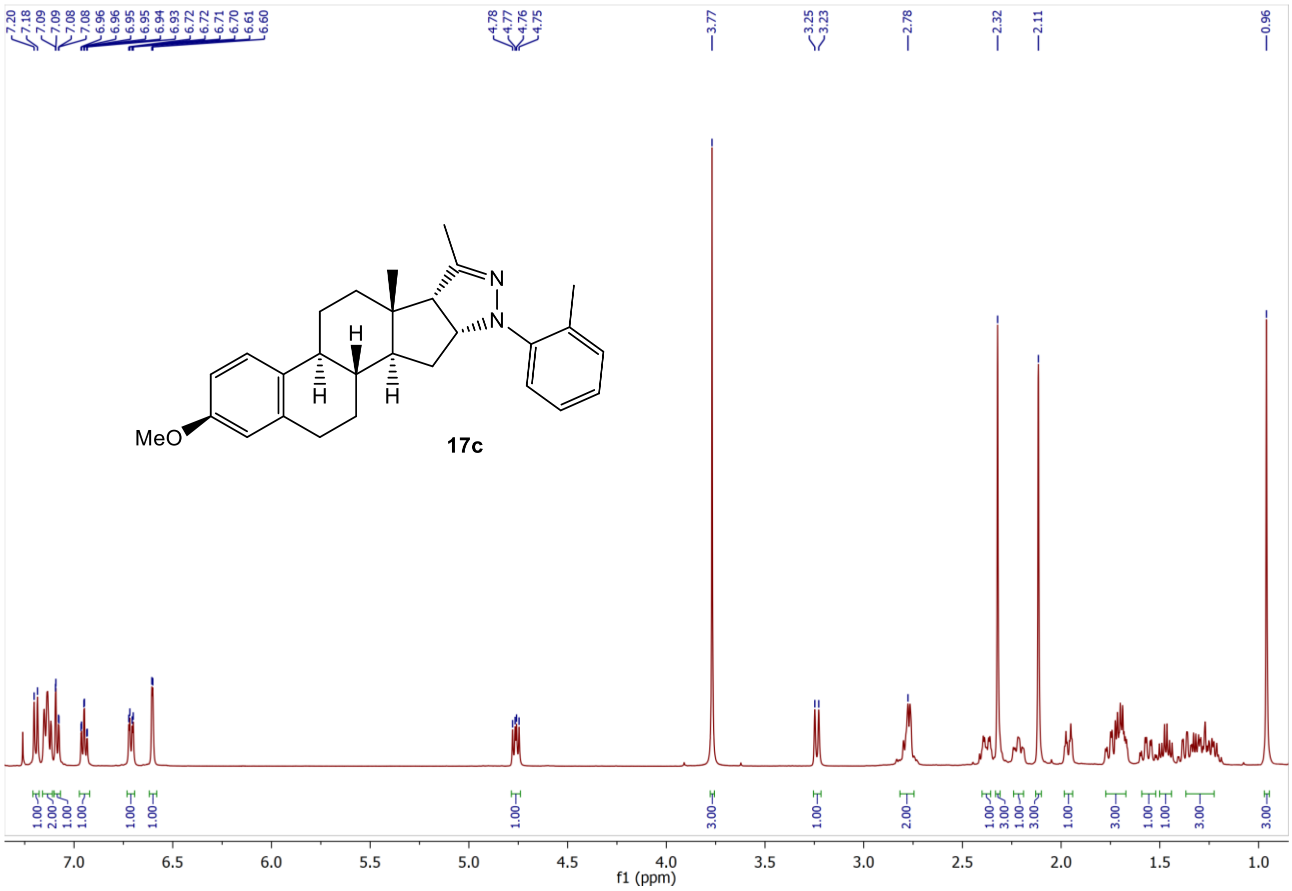
7.20
7.18
7.09
7.09
7.08
7.08
6.96
6.96
6.95
6.95
6.94
6.93
6.72
6.72
6.71
6.70
6.61
6.60
4.78
4.77
4.76
4.75
3.77
3.25
3.23
2.78
2.32
2.11
0.96
N
N
H
H
H
MeO
17c
1.00
2.00
1.00
1.00
1.00
1.00
1.00
3.00
1.00
2.00
1.00
3.00
1.00
3.00
1.00
3.00
1.00
1.00
3.00
3.00
7.0
6.5
6.0
5.5
5.0
4.5
4.0
3.5
3.0
2.5
2.0
1.5
1.0
f1 (ppm)

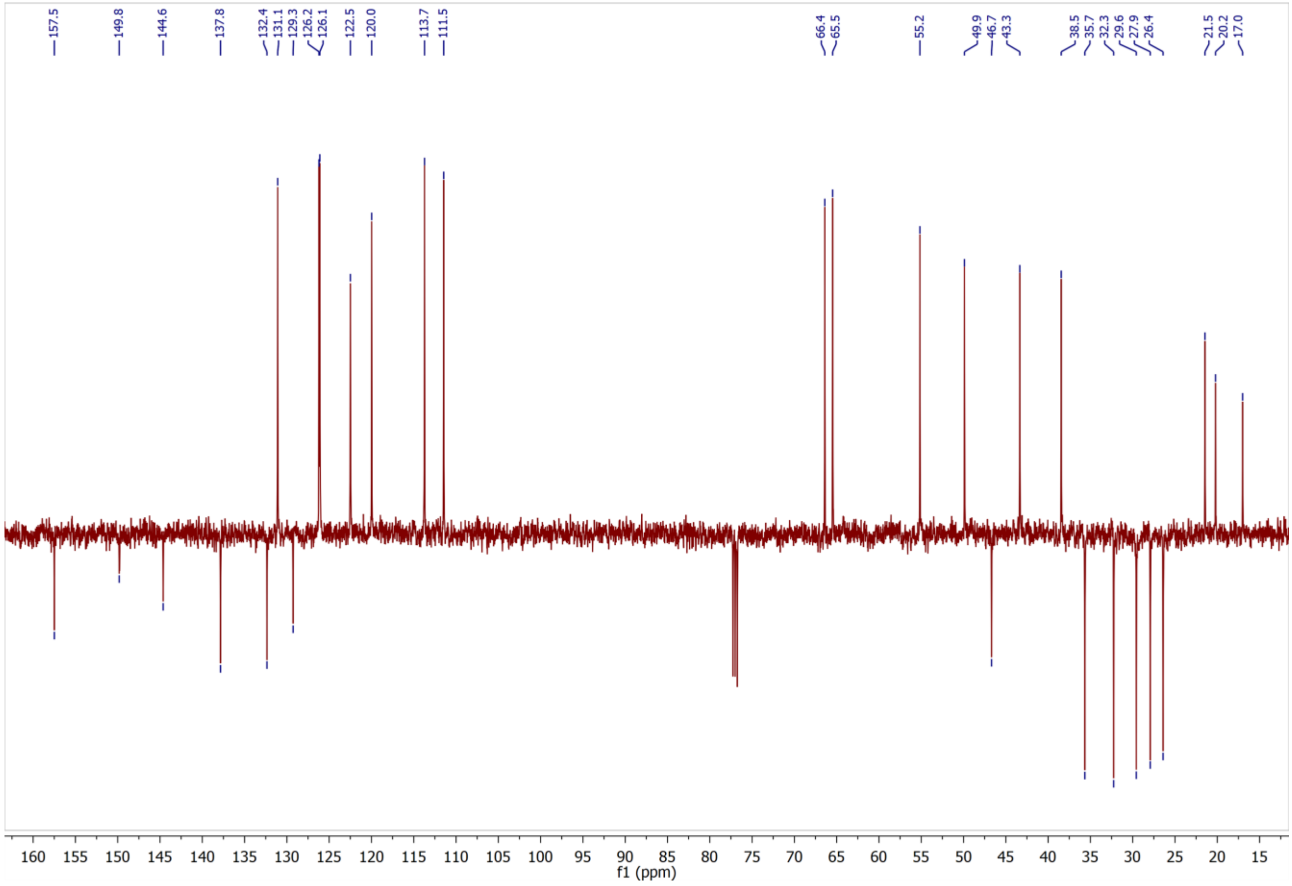
157.5
149.8
144.6
137.8
132.4
131.1
129.3
126.2
126.1
122.5
120.0
113.7
111.5
66.4
65.5
55.2
49.9
46.7
43.3
38.5
35.7
32.3
29.6
27.9
26.4
21.5
20.2
17.0
160
155
150
145
140
135
130
125
120
115
110
105
100
95
90
85
80
75
70
65
60
55
50
45
40
35
30
25
20
15
f1 (ppm)

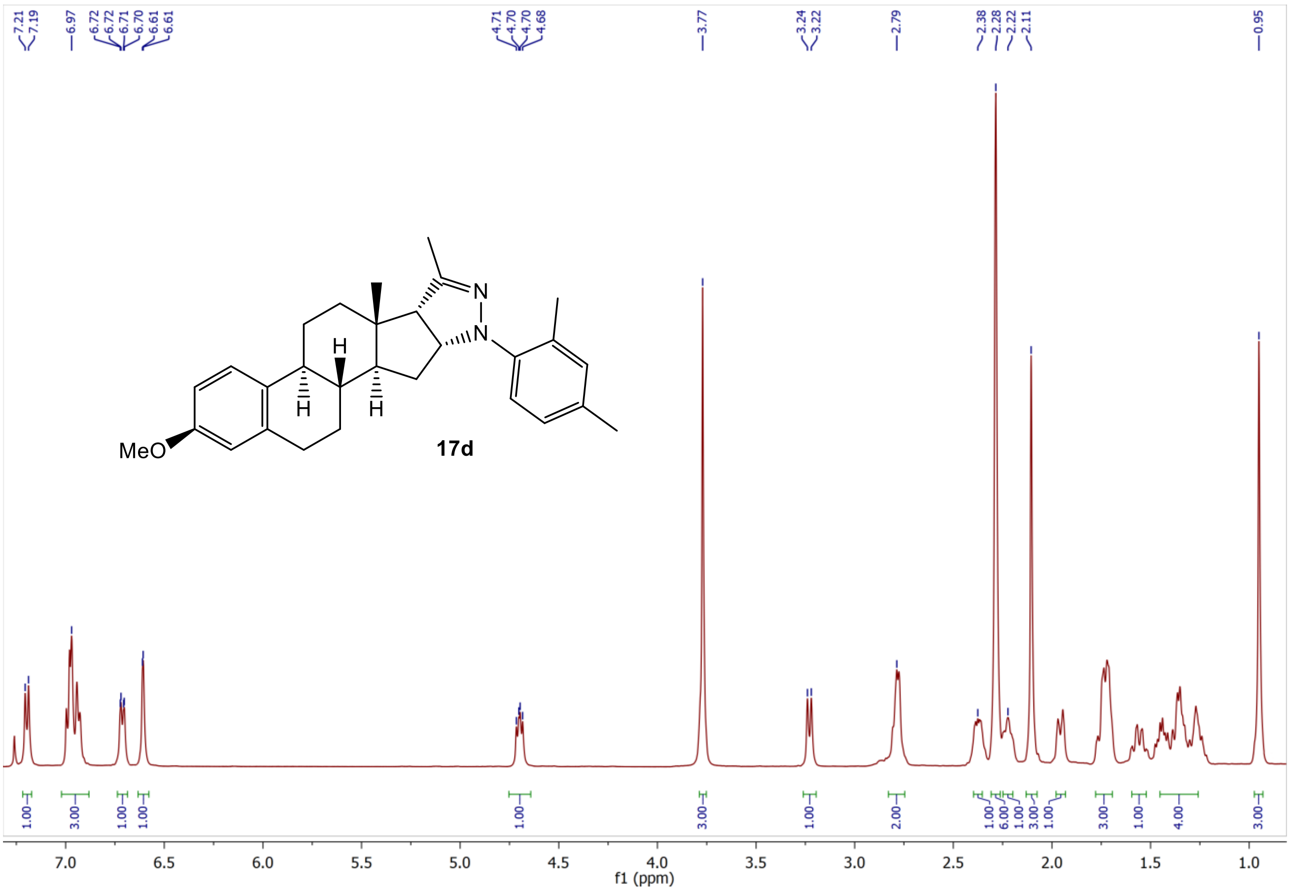
7.21
7.19
6.97
6.72
6.72
6.71
6.70
6.61
6.61
4.71
4.70
4.70
4.68
3.77
3.24
3.22
2.79
2.38
2.28
2.22
2.11
0.95
MeO
H
H
H
N
N
17d
1.00
3.00
1.00
1.00
1.00
3.00
1.00
2.00
1.00
6.00
1.00
3.00
1.00
3.00
1.00
4.00
3.00
7.0
6.5
6.0
5.5
5.0
4.5
4.0
3.5
3.0
2.5
2.0
1.5
1.0
f1 (ppm)

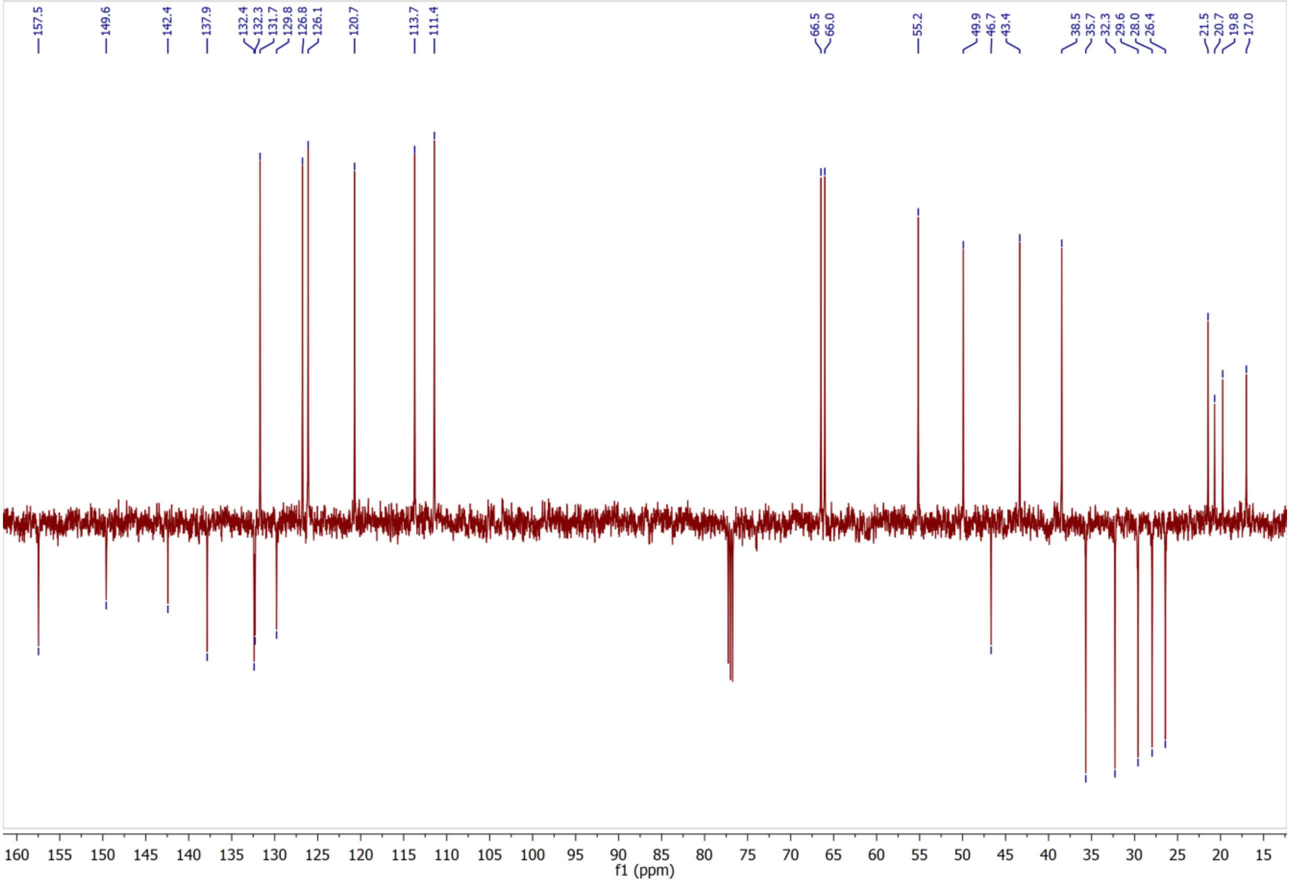
157.5
149.6
142.4
137.9
132.4
132.3
131.7
129.8
126.8
126.1
120.7
113.7
111.4
66.5
66.0
55.2
49.9
46.7
43.4
38.5
35.7
32.3
29.6
28.0
26.4
21.5
20.7
19.8
17.0
160
155
150
145
140
135
130
125
120
115
110
105
100
95
90
85
80
75
70
65
60
55
50
45
40
35
30
25
20
15
f1 (ppm)

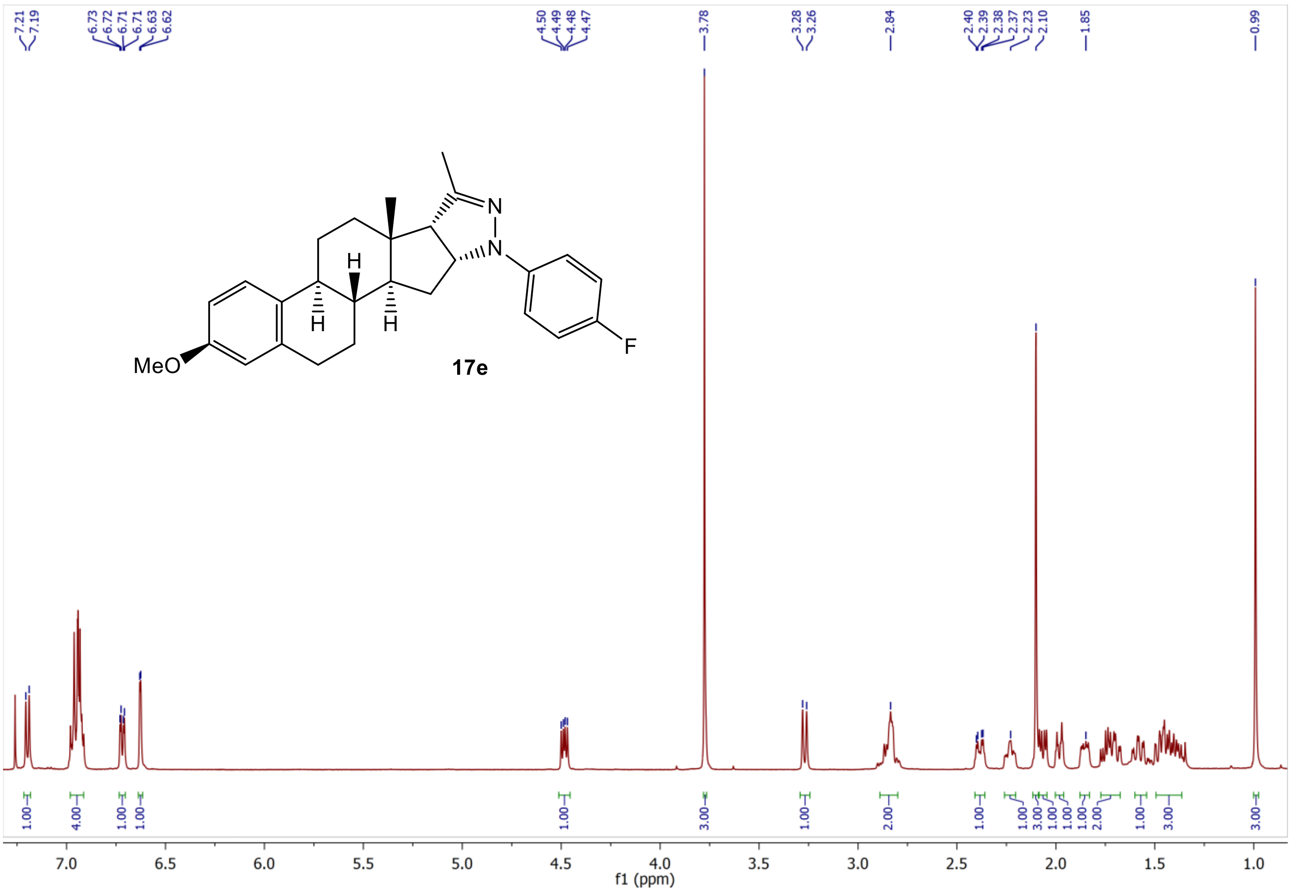

MeO
17e
F
N
H
f1 (ppm)

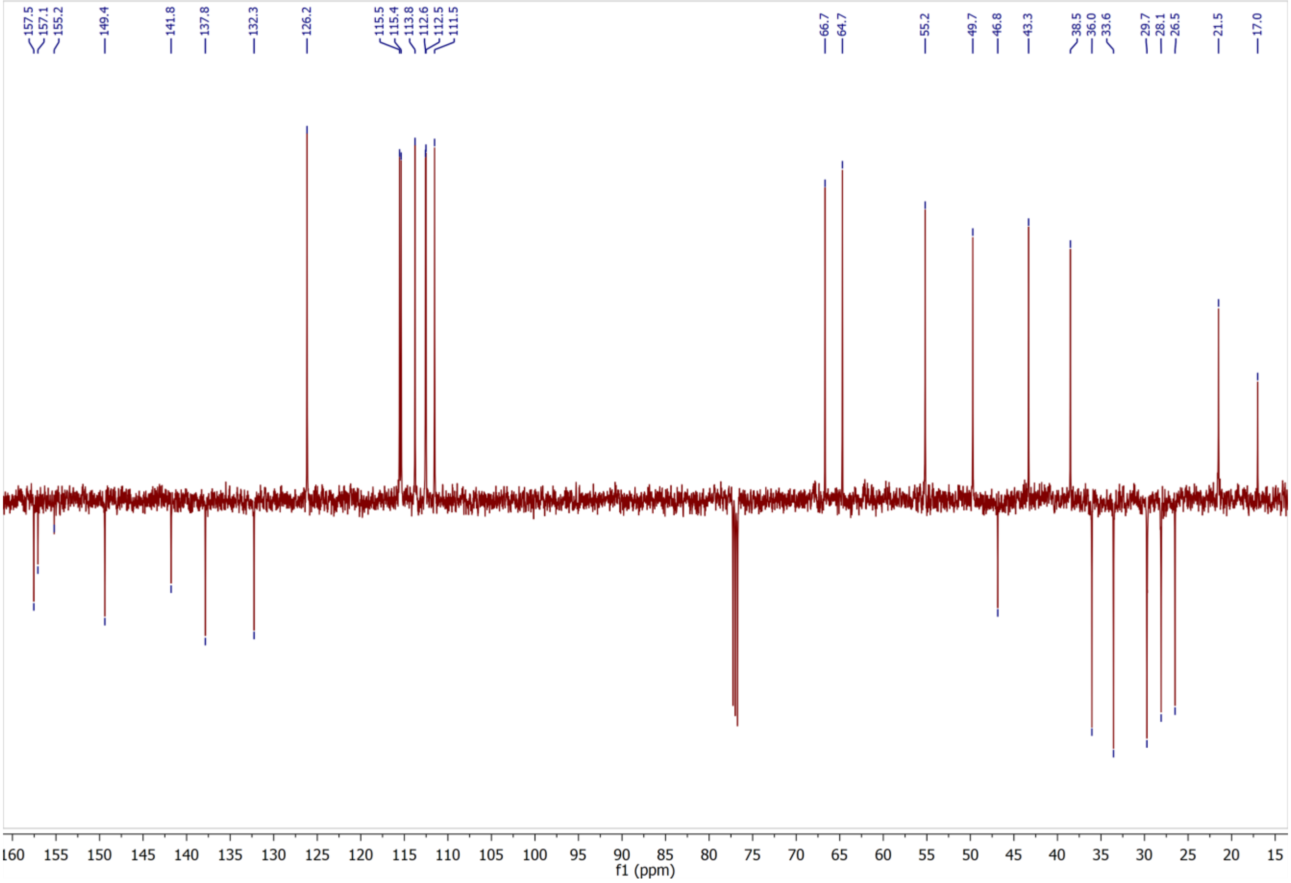

f1 (ppm)

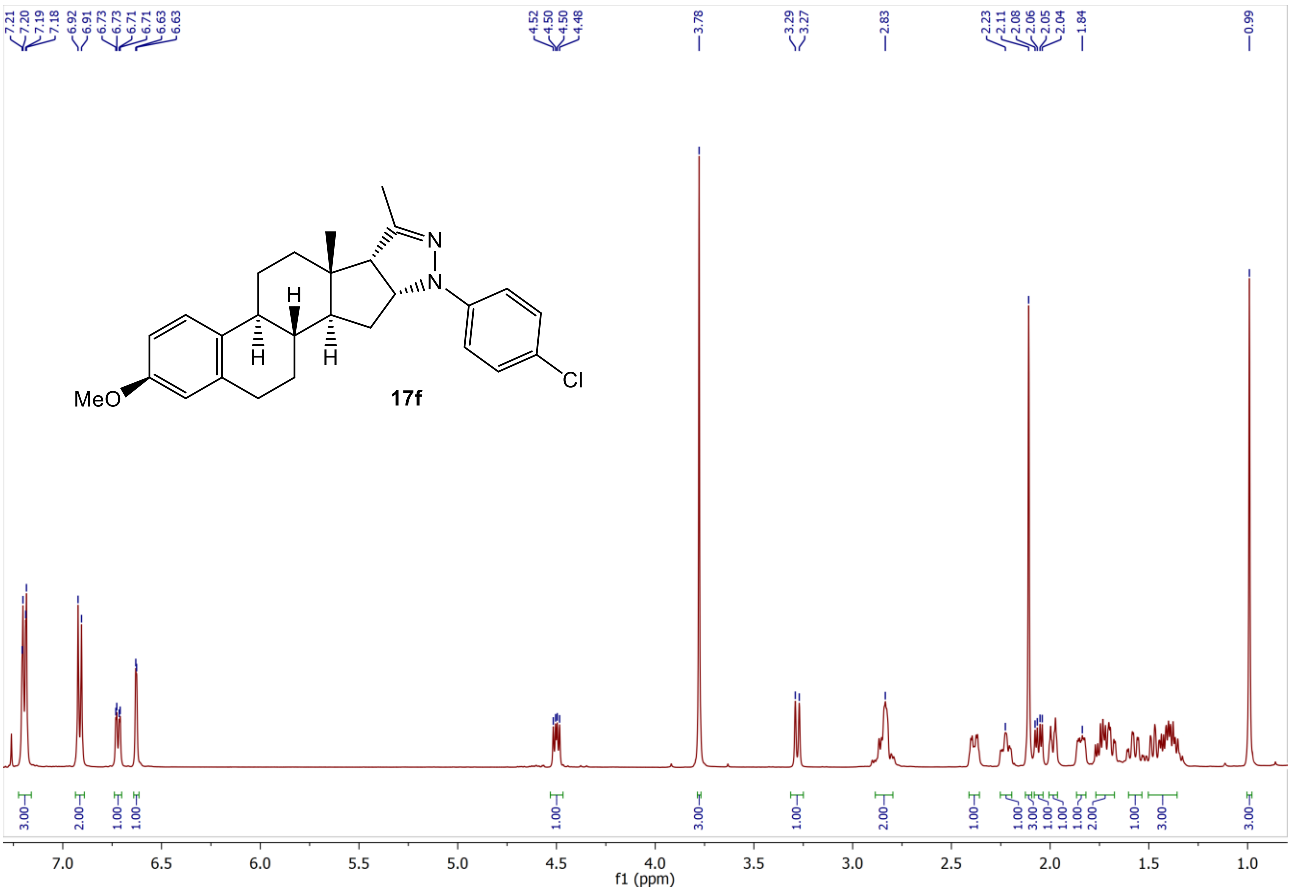
7.21
7.20
7.19
7.18
6.92
6.91
6.73
6.73
6.71
6.71
6.63
6.63
4.52
4.50
4.50
4.48
3.78
3.29
3.27
2.83
2.23
2.11
2.08
2.06
2.05
2.04
1.84
0.99
N
N
H
H
H
MeO
Cl
17f
3.00
2.00
1.00
1.00
1.00
3.00
1.00
2.00
1.00
1.00
3.00
1.00
1.00
1.00
2.00
1.00
3.00
3.00
7.0 6.5 6.0 5.5 5.0 4.5 4.0 3.5 3.0 2.5 2.0 1.5 1.0
f1 (ppm)

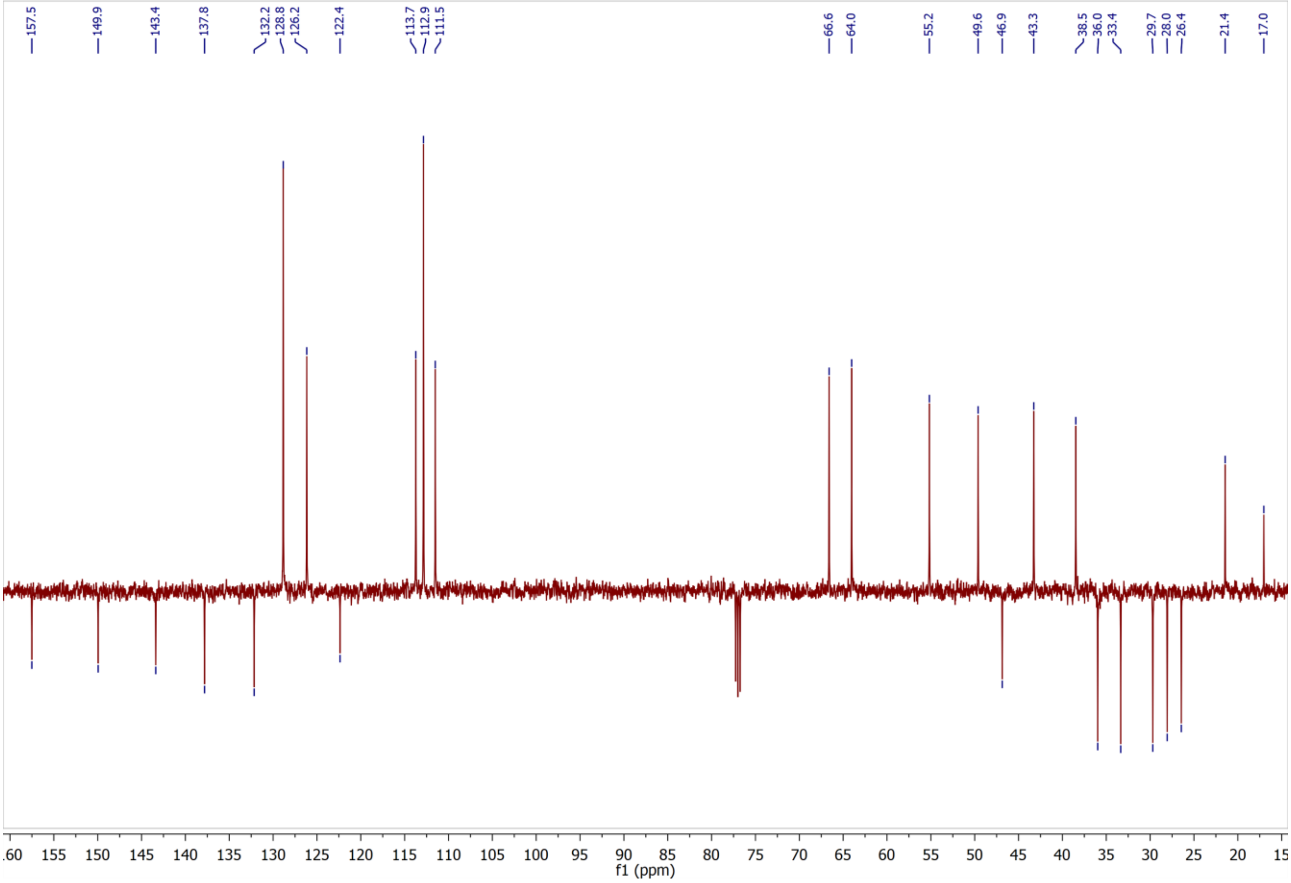
157.5
149.9
143.4
137.8
132.2
128.8
126.2
122.4
113.7
112.9
111.5
66.6
64.0
55.2
49.6
46.9
43.3
38.5
36.0
33.4
29.7
28.0
26.4
21.4
17.0
160 155 150 145 140 135 130 125 120 115 110 105 100 95 90 85 80 75 70 65 60 55 50 45 40 35 30 25 20 15
f1 (ppm)

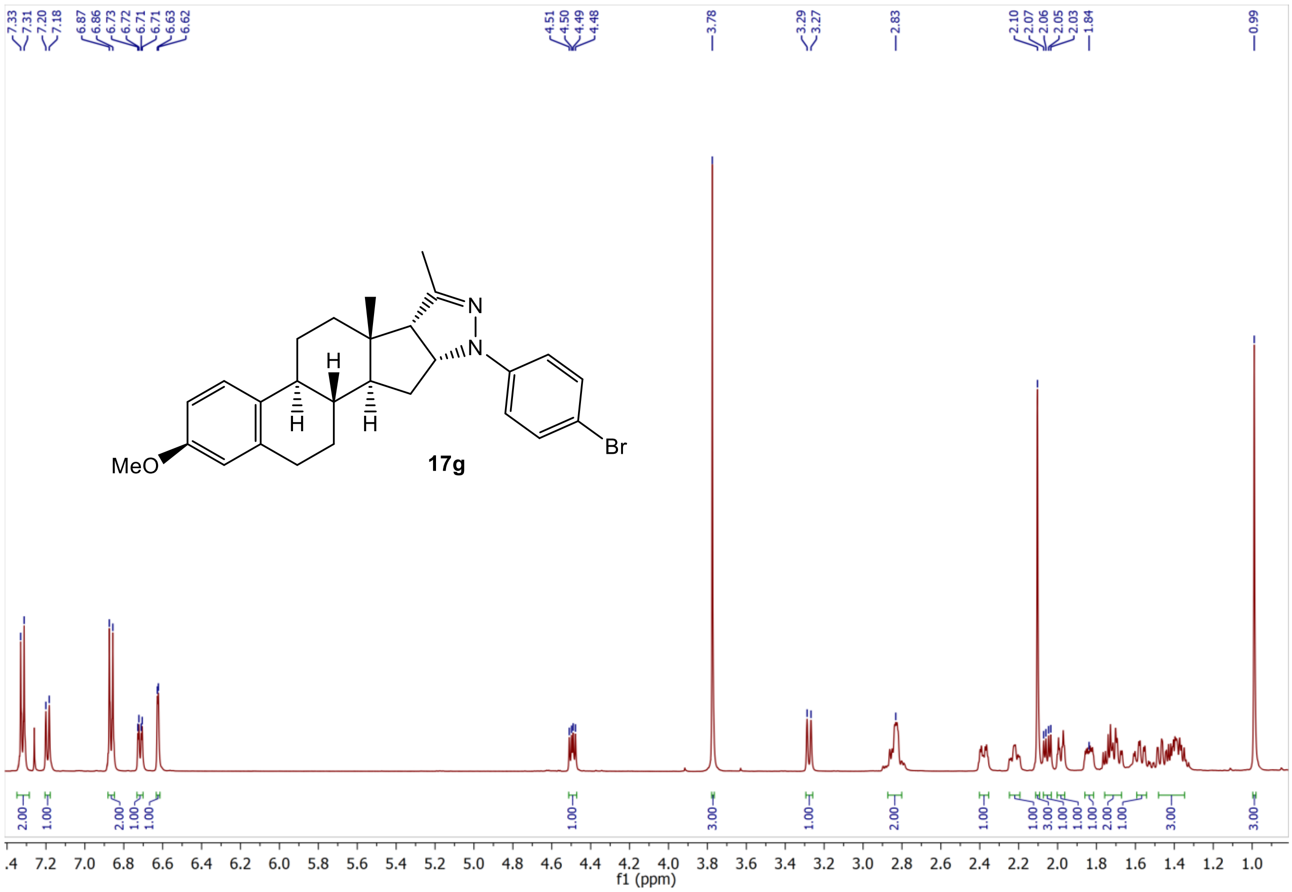
MeO
Br
17g
f1 (ppm)

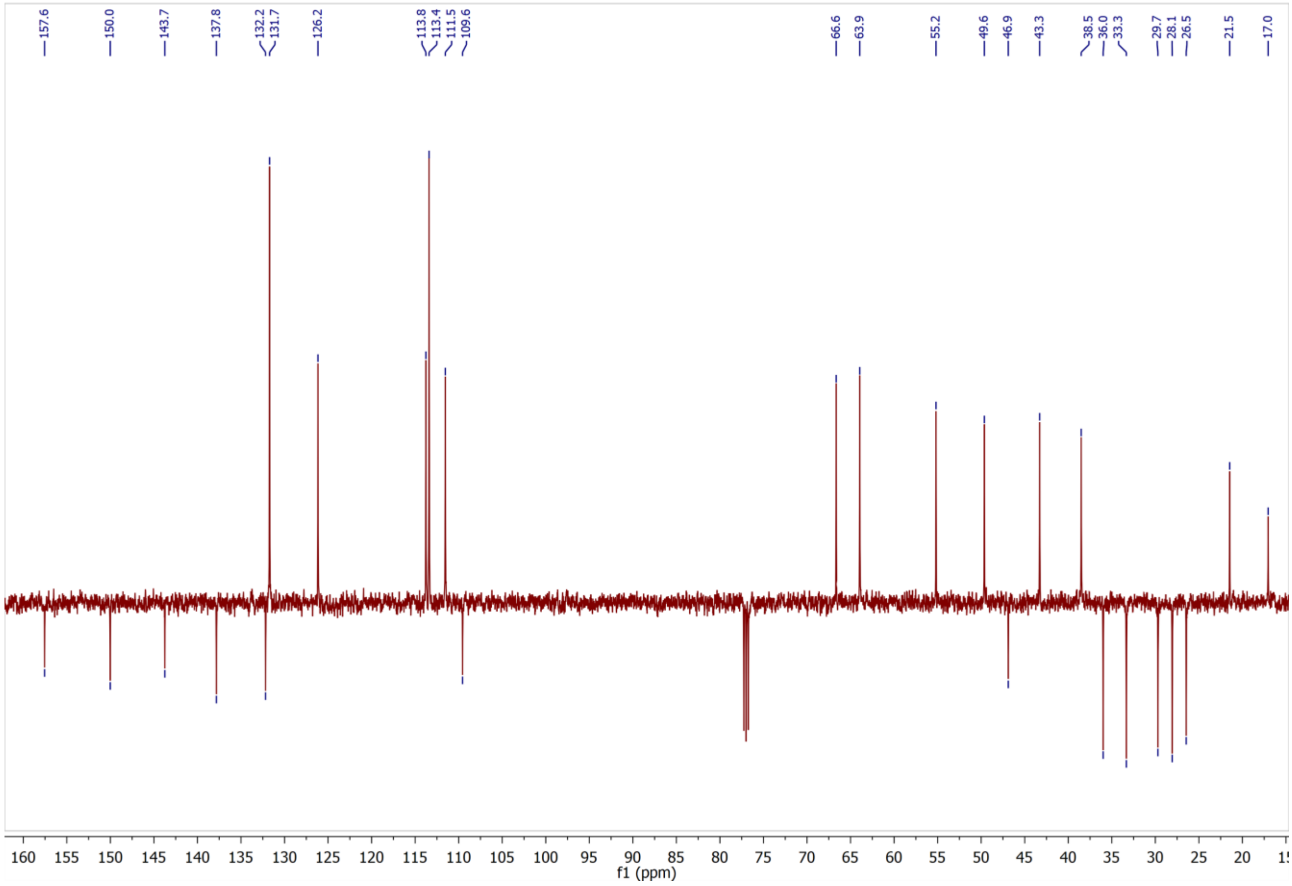
157.6
150.0
143.7
137.8
132.2
131.7
126.2
113.8
113.4
111.5
109.6
66.6
63.9
55.2
49.6
46.9
43.3
38.5
36.0
33.3
29.7
28.1
26.5
21.5
17.0
f1 (ppm)

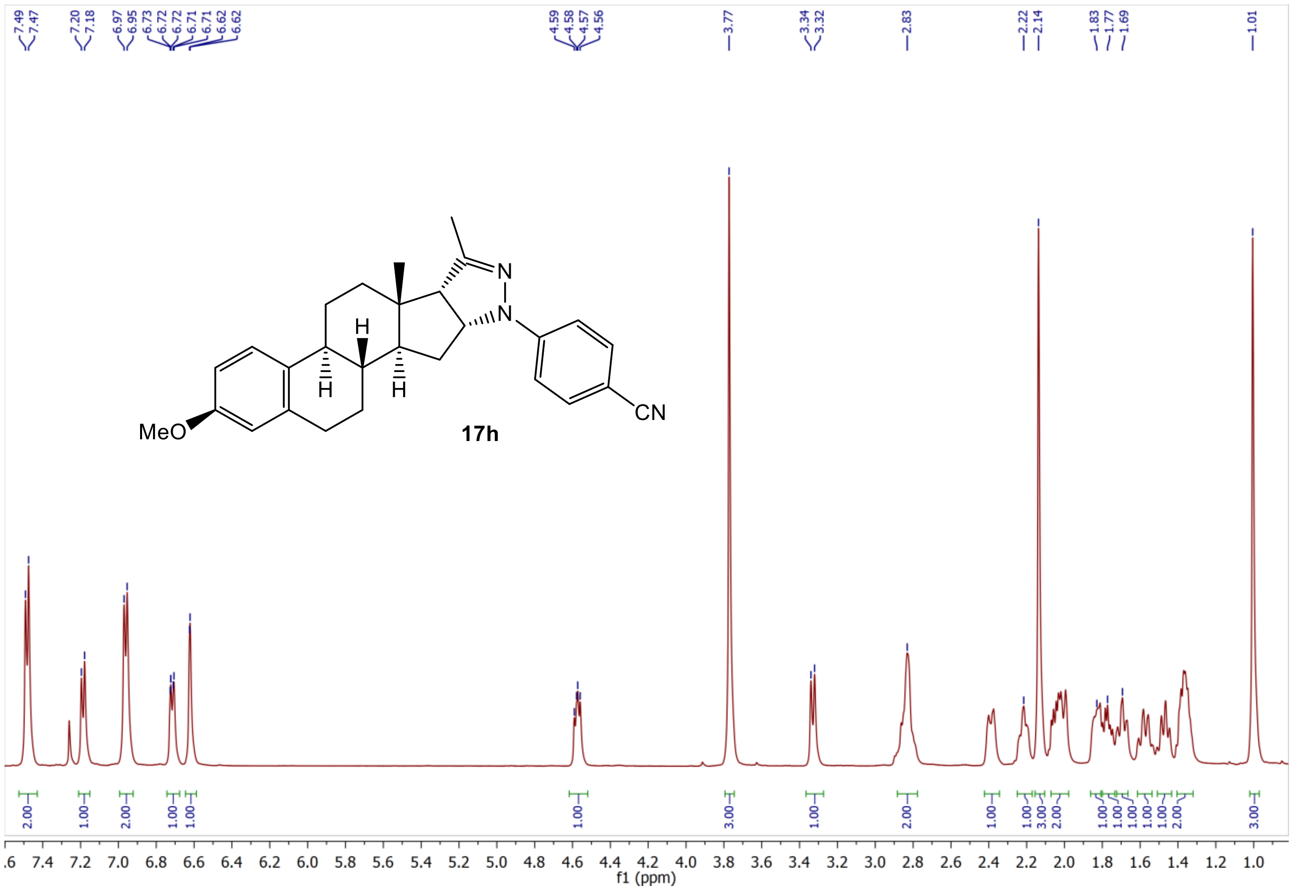
7.49
7.47
7.20
7.18
6.97
6.95
6.73
6.72
6.72
6.71
6.71
6.62
6.62
4.59
4.58
4.57
4.56
3.77
3.34
3.32
2.83
2.22
2.14
1.83
1.77
1.69
1.01
MeO
N
N
H
H
H
CN
17h
2.00
1.00
2.00
1.00
1.00
1.00
3.00
1.00
2.00
1.00
1.00
3.00
2.00
1.00
1.00
1.00
1.00
1.00
2.00
3.00
f1 (ppm)

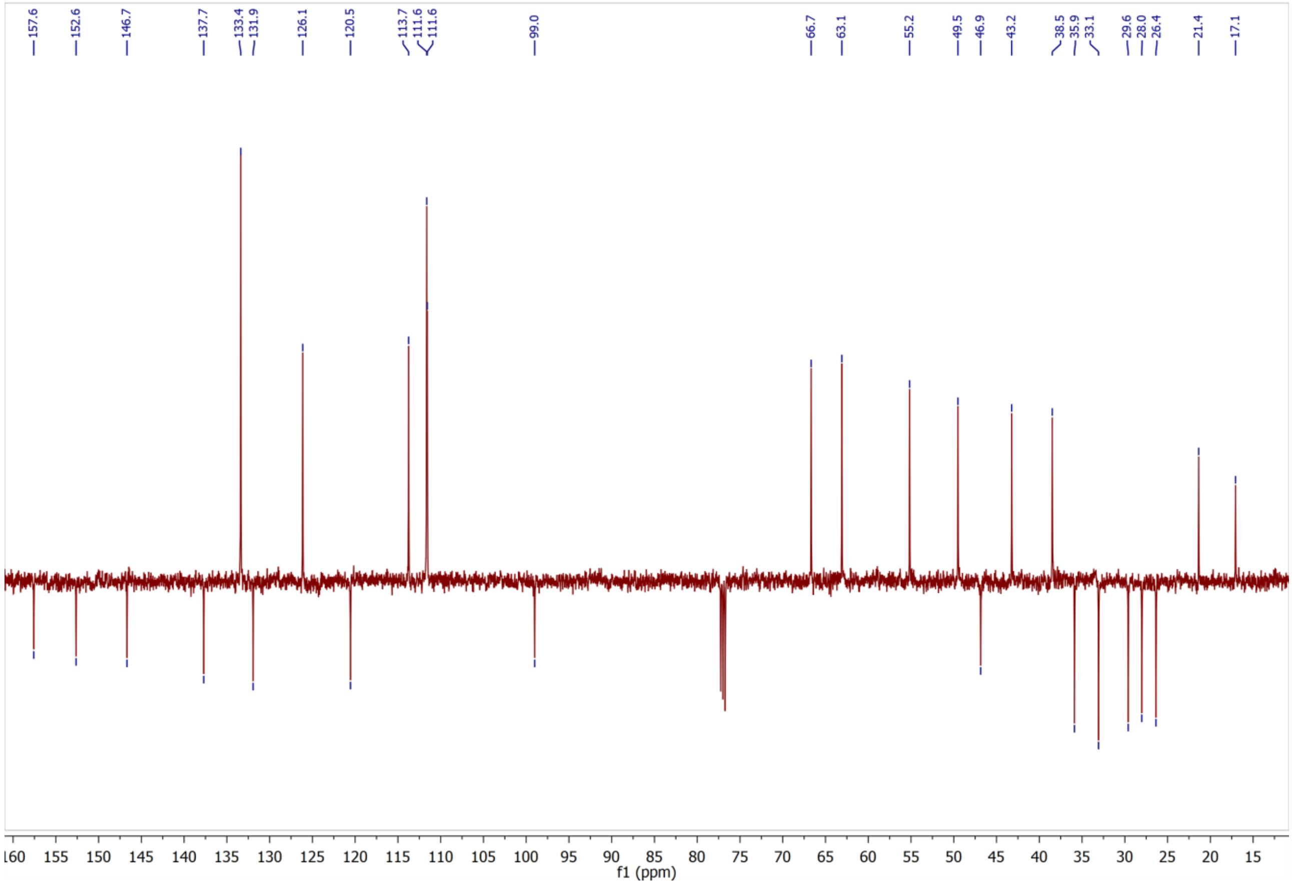
157.6
152.6
146.7
137.7
133.4
131.9
126.1
120.5
113.7
111.6
111.6
99.0
66.7
63.1
55.2
49.5
46.9
43.2
38.5
35.9
33.1
29.6
28.0
26.4
21.4
17.1
f1 (ppm)

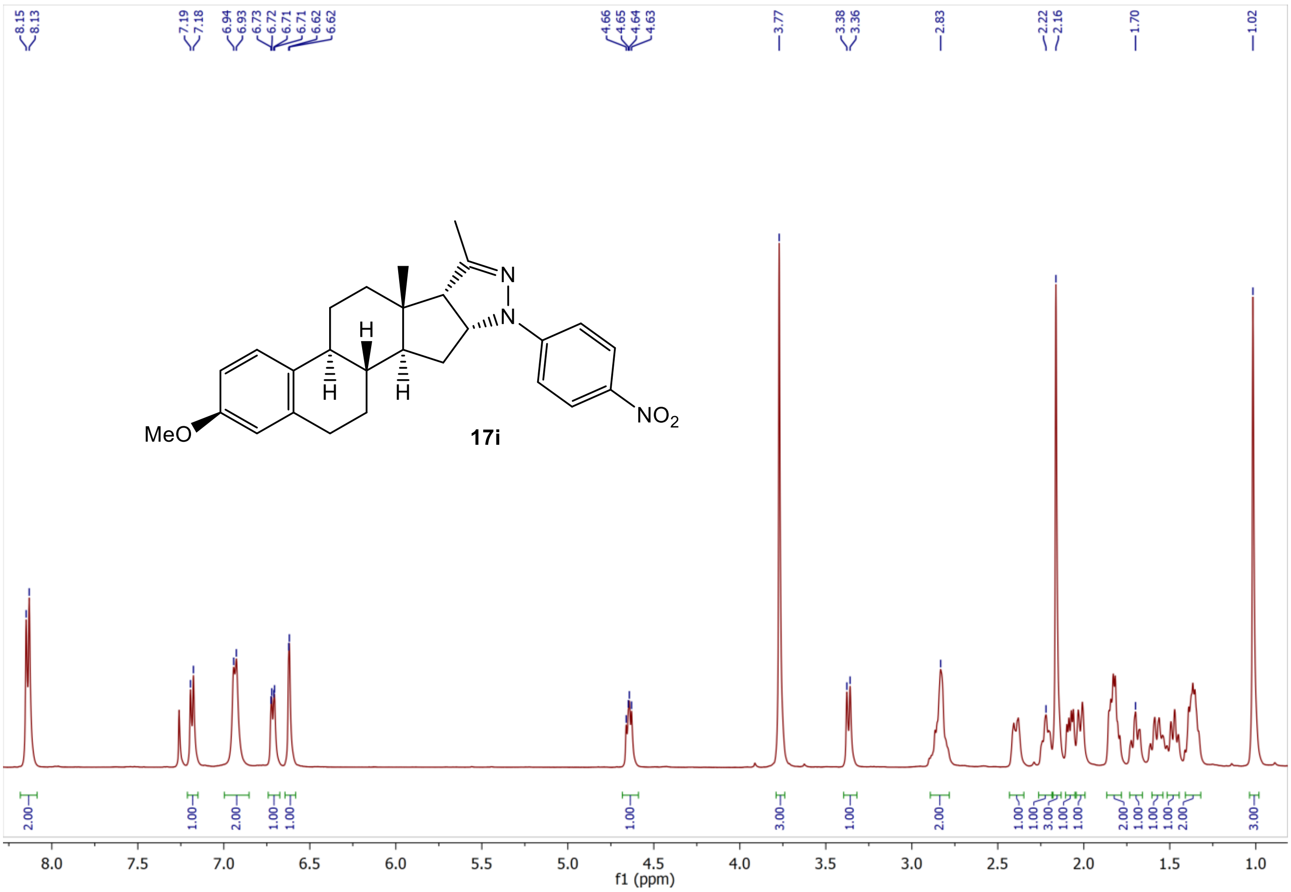
8.15
8.13
7.19
7.18
6.94
6.93
6.73
6.72
6.71
6.71
6.62
6.62
4.66
4.65
4.64
4.63
3.77
3.38
3.36
2.83
2.22
2.16
1.70
1.02
N
N
H
H
H
MeO
NO2
17i
2.00
1.00
2.00
1.00
1.00
1.00
3.00
1.00
2.00
1.00
1.00
3.00
1.00
1.00
2.00
1.00
1.00
1.00
2.00
3.00
8.0
7.5
7.0
6.5
6.0
5.5
5.0
4.5
4.0
3.5
3.0
2.5
2.0
1.5
1.0
f1 (ppm)

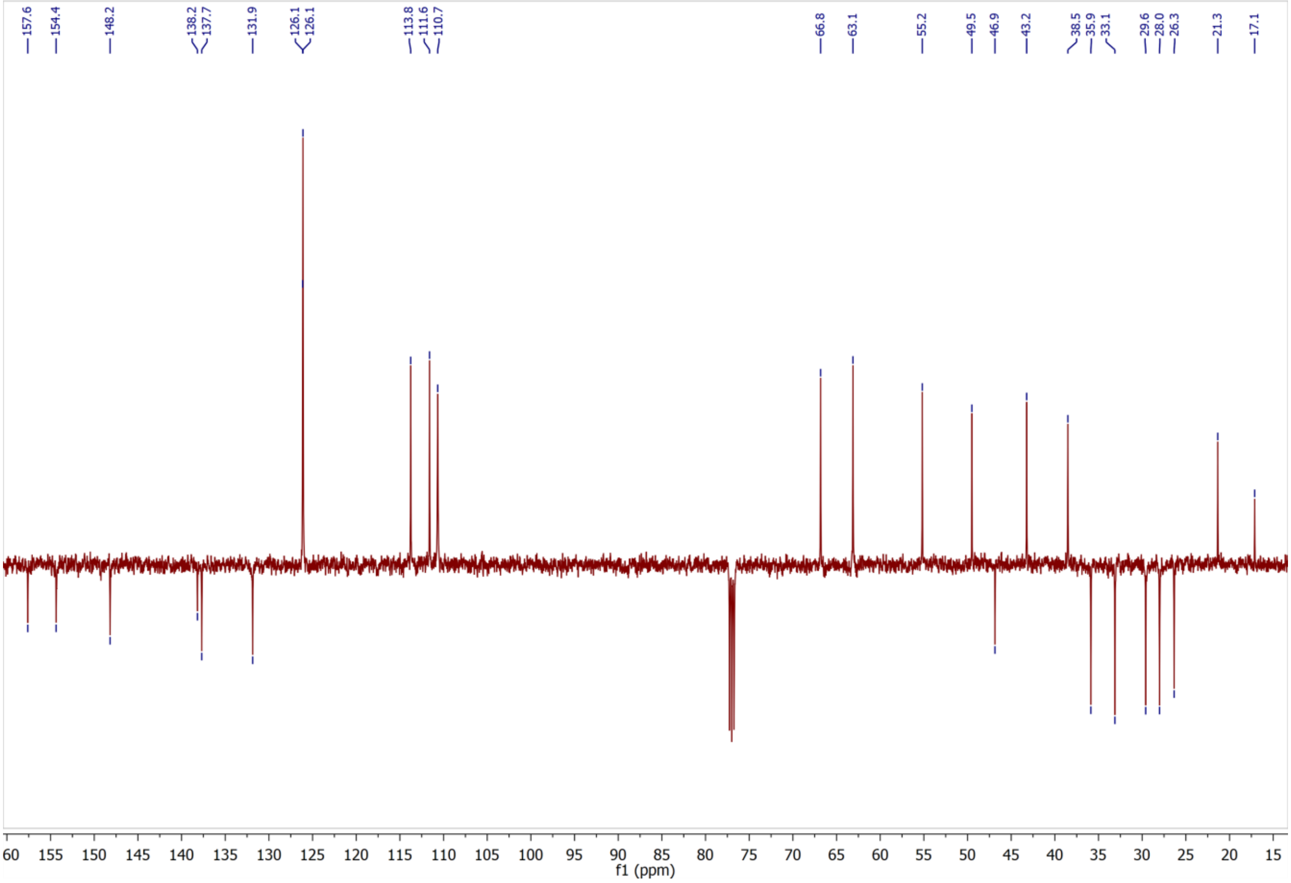
157.6
154.4
148.2
138.2
137.7
131.9
126.1
126.1
113.8
111.6
110.7
66.8
63.1
55.2
49.5
46.9
43.2
38.5
35.9
33.1
29.6
28.0
26.3
21.3
17.1
60
155
150
145
140
135
130
125
120
115
110
105
100
95
90
85
80
75
70
65
60
55
50
45
40
35
30
25
20
15
f1 (ppm)

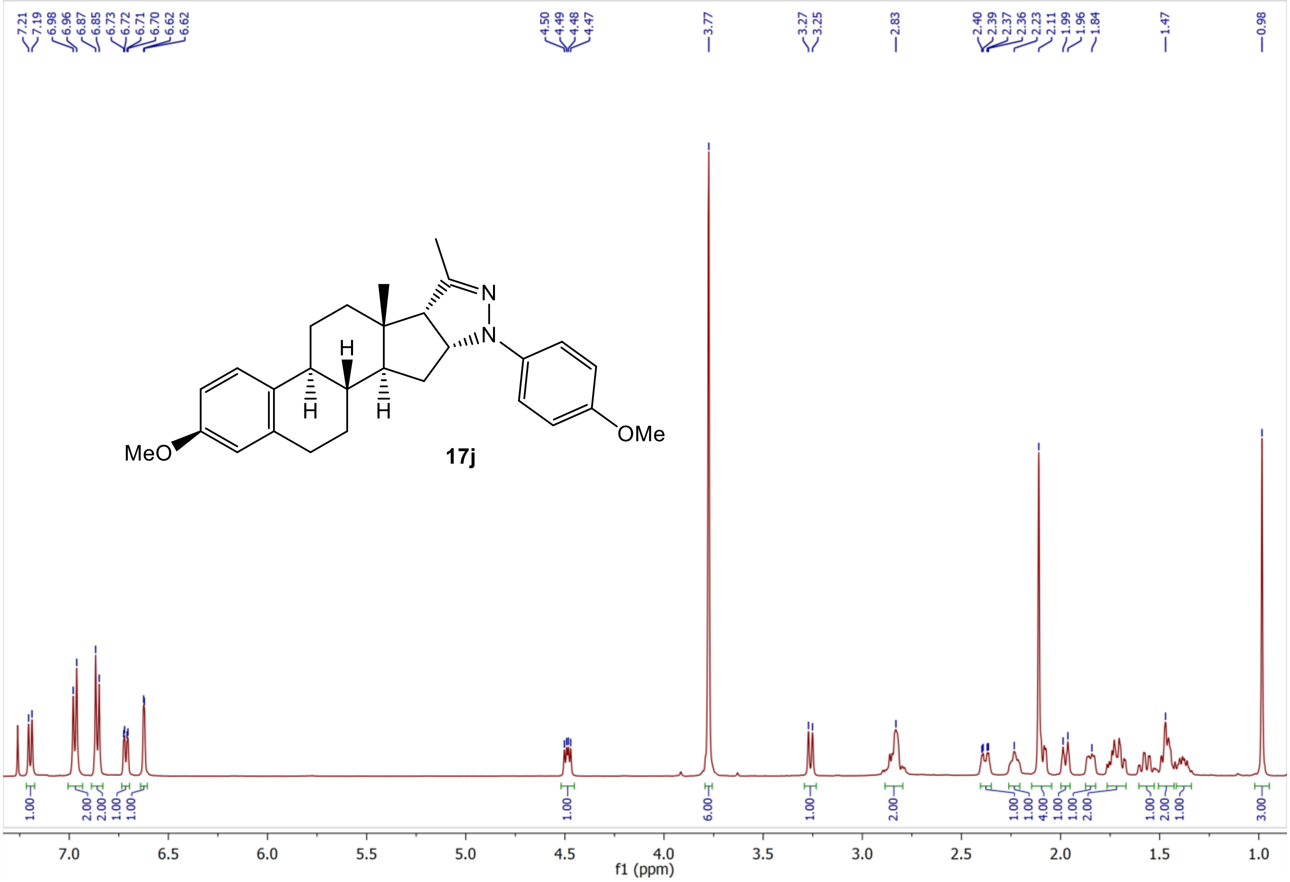

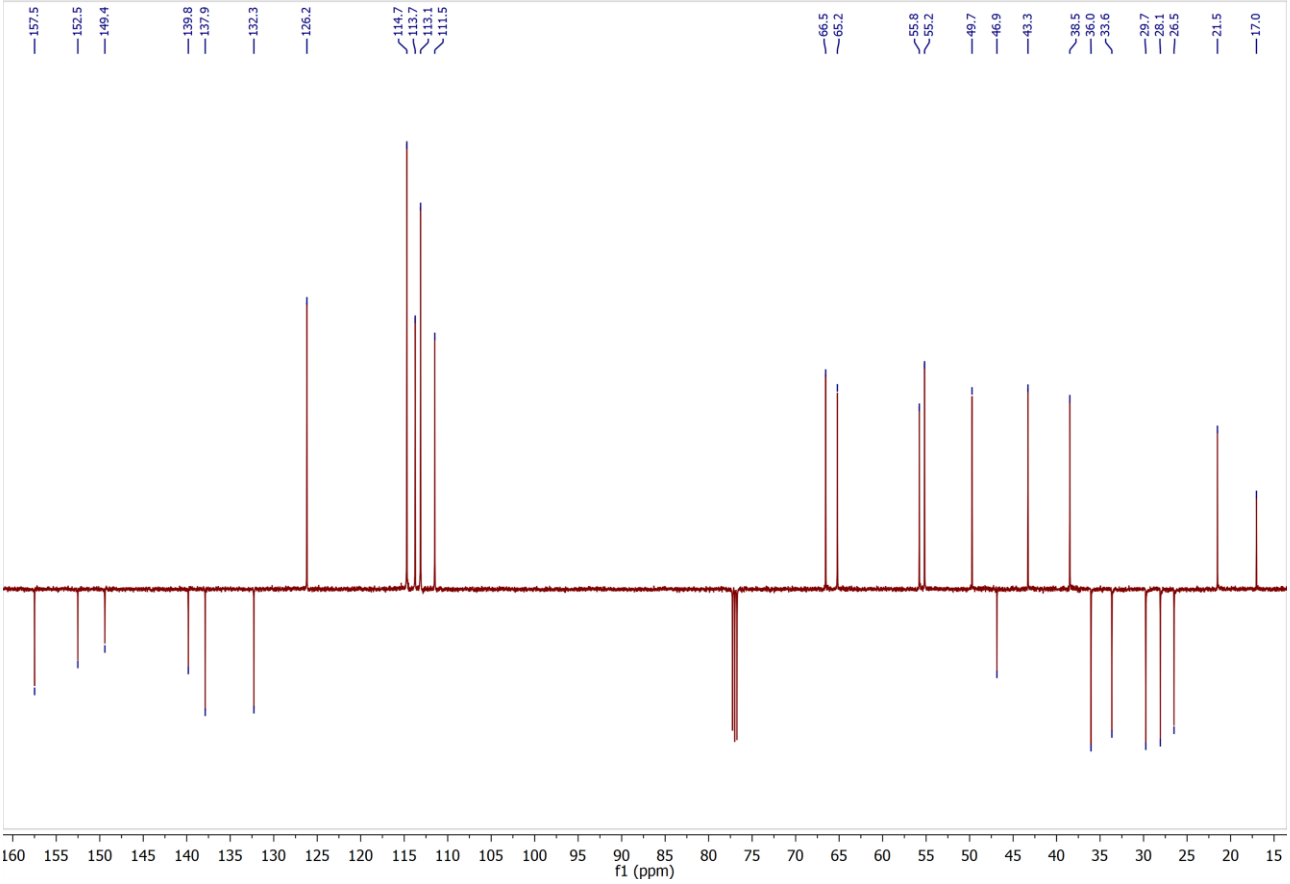

HSQC spectrum of **17j**

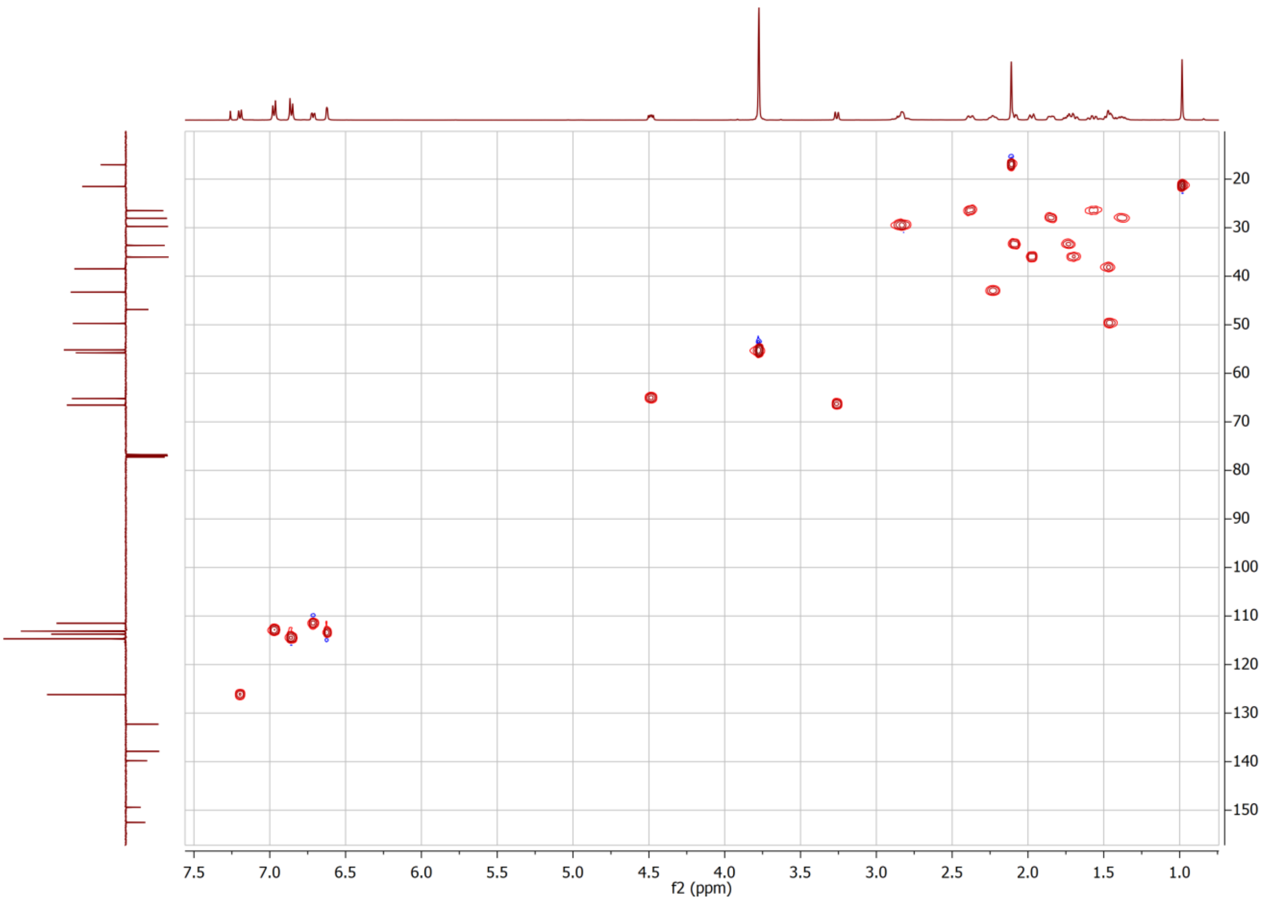

HMBC spectrum of **17j**

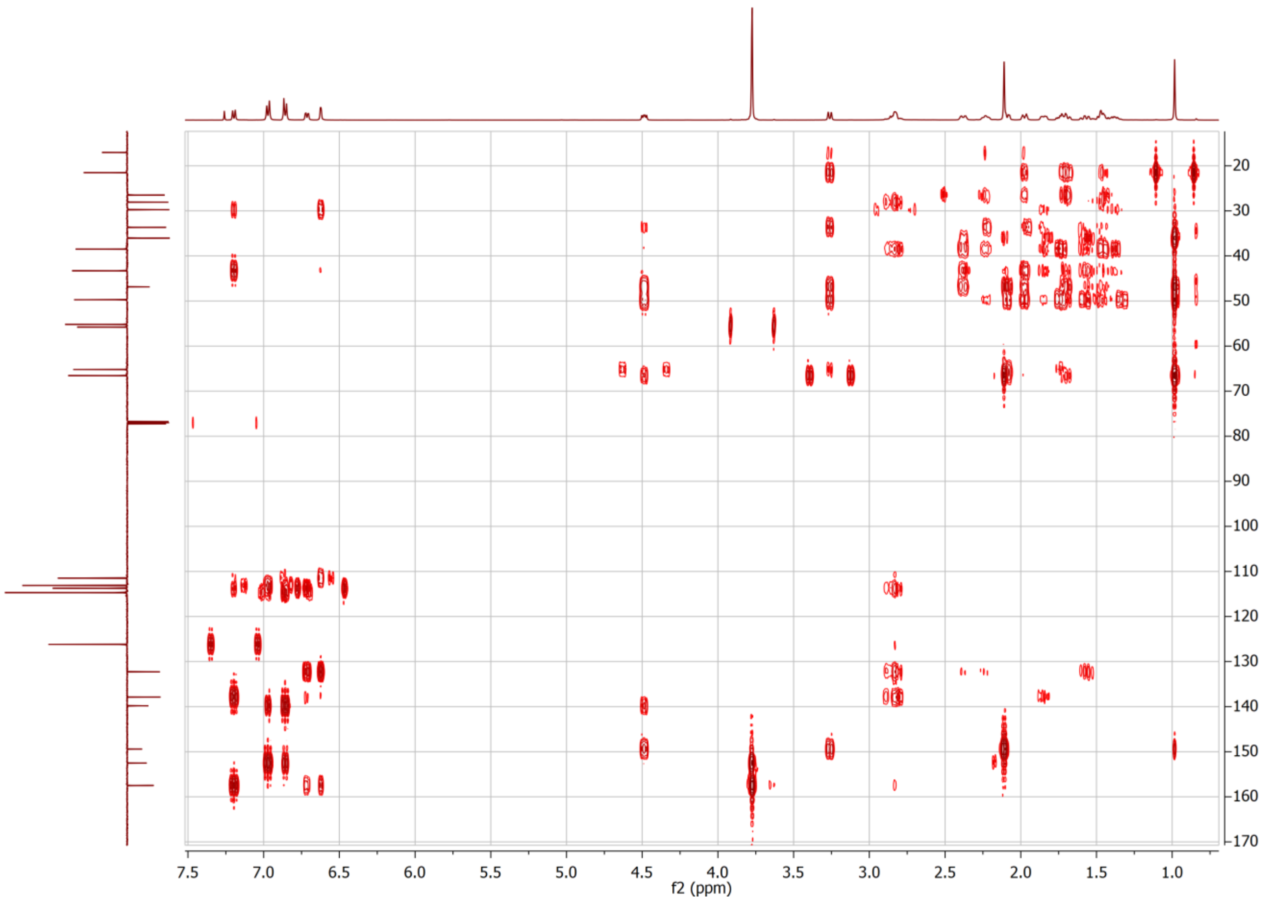

NOESY spectrum of **17j**

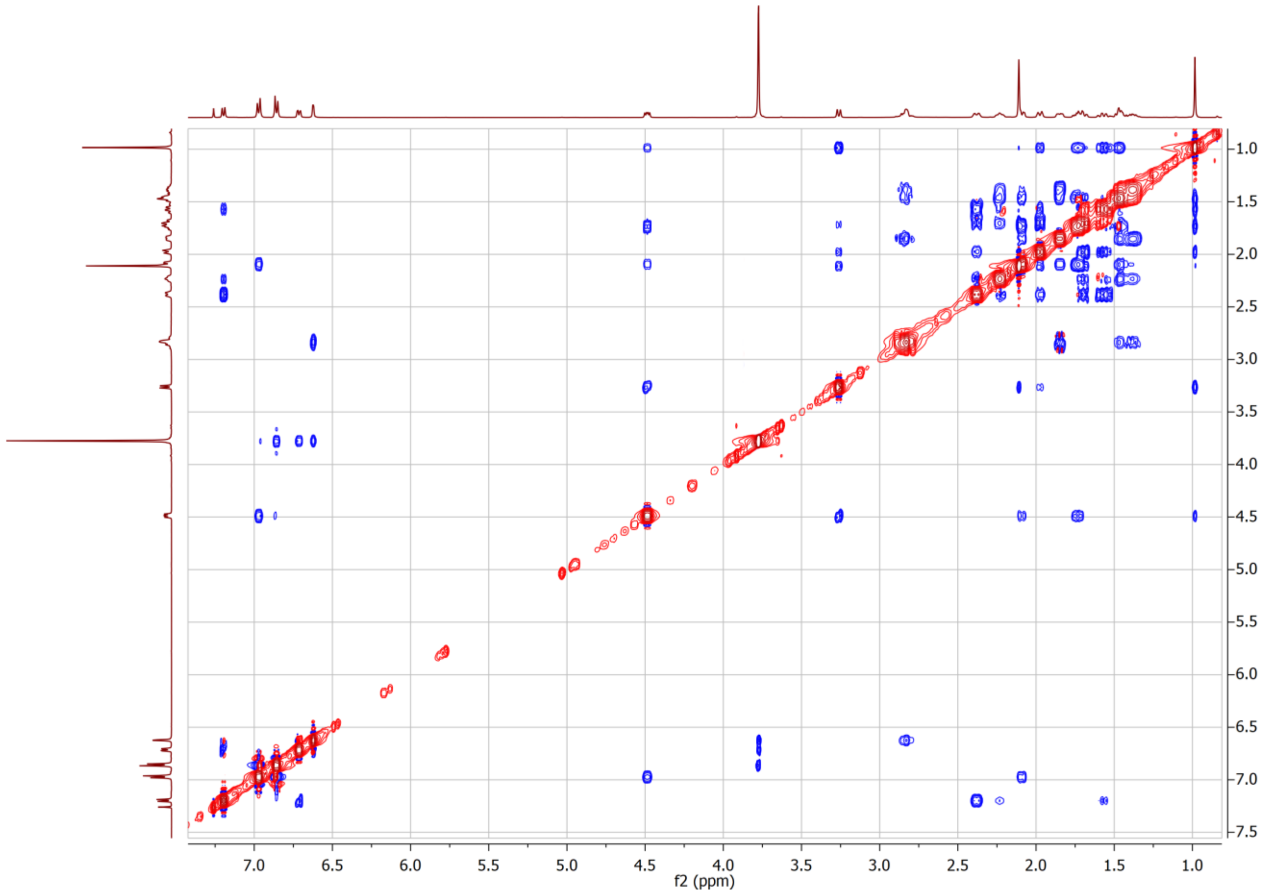

COSY spectrum of **17j**

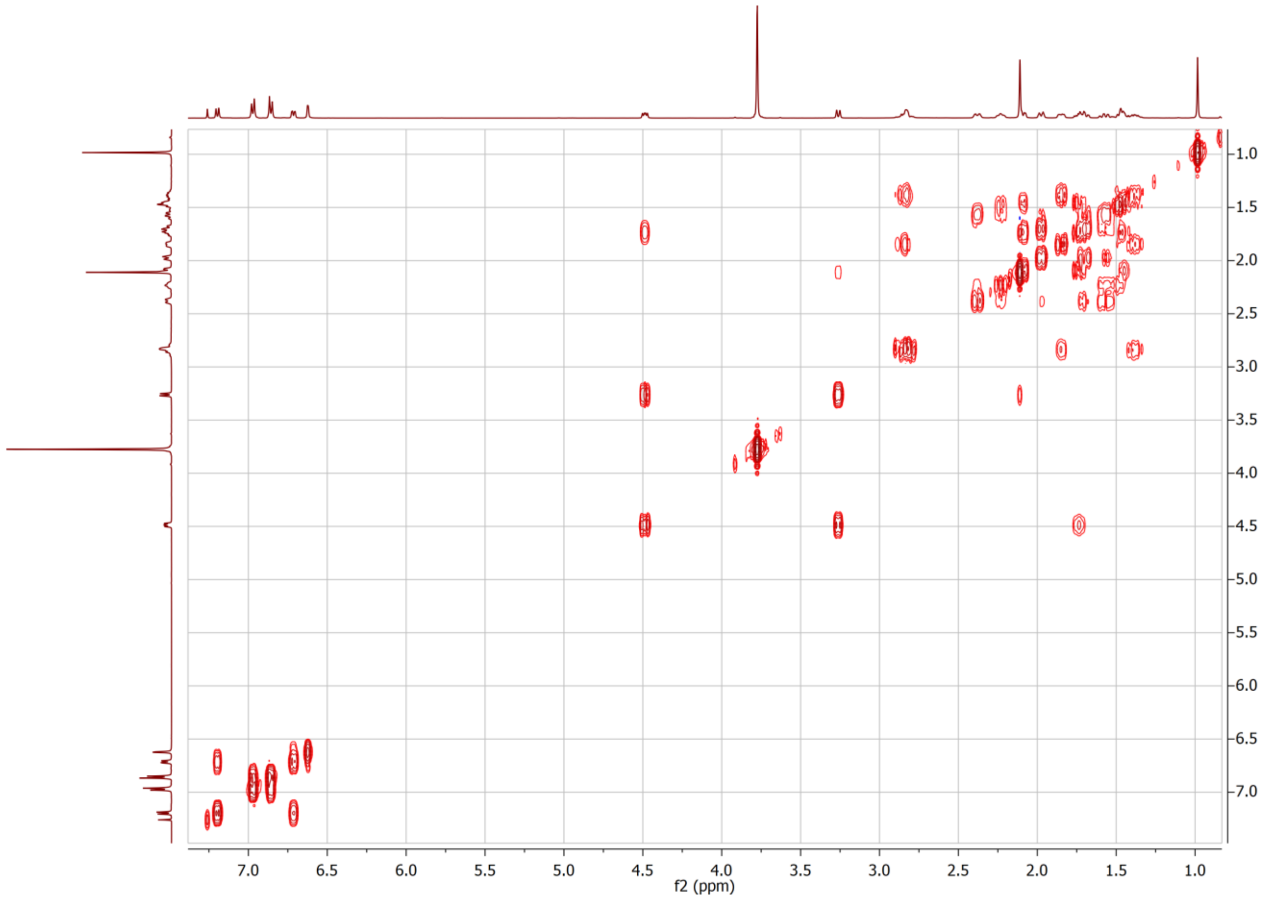

Supplement: Supplementary file 1 [file molecules-24-00569-s001.pdf]
